# Diversification of CpG-Island Promoters Revealed by Comparative Analysis Between Human and Rhesus Monkey Genomes

**DOI:** 10.1007/s00335-020-09844-2

**Published:** 2020-07-09

**Authors:** Saki Aoto, Mayu Fushimi, Kei Yura, Kohji Okamura

**Affiliations:** 1 Medical Genome Center, National Center for Child Health and Development, Tokyo, 157-8535 Japan; 2 Department of Biology, Faculty of Science, Ochanomizu University, Tokyo, 112-8610 Japan; 3 Department of Systems BioMedicine, National Center for Child Health and Development, Tokyo, 157-8535 Japan

## Abstract

While CpG dinucleotides are significantly reduced compared to other dinucleotides in mammalian genomes, they can congregate and form CpG islands, which localize around the 5ʹ regions of genes, where they function as promoters. CpG-island promoters are generally unmethylated and are often found in housekeeping genes. However, their nucleotide sequences and existence per se are not conserved between humans and mice, which may be due to evolutionary gain and loss of the regulatory regions. In this study, human and rhesus monkey genomes, with moderately conserved sequences, were compared at base resolution. Using transcription start site data, we first validated our methods’ ability to identify orthologous promoters and indicated a limitation using the 5ʹ end of curated gene models, such as NCBI RefSeq, as their transcription start sites. We found that, in addition to deamination mutations, insertions and deletions of bases, repeats, and long fragments contributed to the mutations of CpG dinucleotides. We also observed that the G + C contents tended to change in CpG-poor environments, while CpG content was altered in G + C-rich environments. While loss of CpG islands can be caused by gradual decreases in CpG sites, gain of these islands appear to require two distinct nucleotide altering steps. Taken together, our findings provide novel insights into the process of acquisition and diversification of CpG-island promoters in vertebrates.

**Electronic supplementary material:**

The online version of this article (10.1007/s00335-020-09844-2) contains supplementary material, which is available to authorized users.

## Introduction

In vertebrate genomic sequences, the content of CpG dinucleotides is significantly lower than expected based on the nucleotide composition (Bird 1980), which is likely due to DNA methylation. Methylated CpG sites are readily mutated to TpG, or its complementary dinucleotide CpA, as spontaneous deamination of methylated cytosine residues cannot be recognized by the DNA repair system (Duncan and Miller 1980). However, a limited number of unmethylated CpG sites congregate in a specific location, forming a CpG island. Many islands are located near the 5ʹ region of gene loci and are implicated in gene expression regulation (Antequera 2003). Invertebrate organisms also retain the DNA methylation system that depends on the dinucleotide. In contrast to the sparse and localized presence of vertebrate CpG islands, invertebrate CpG sites form a much longer tract that distributes in a mosaic manner (Tweedie et al. 1997). For example, in ascidian, dense, and sparse CpG tracts alternately appear in tens or hundreds of kilobases in the genome (Okamura et al. 2010). Thus, as vertebrates and invertebrates have distinct gene regulatory mechanisms (Zemach and Zilberman 2010), understanding the evolutionary gain of CpG islands will provide greater insight into the human gene regulatory system.

Approximately half of all human protein-coding genes bear a CpG island in the promoter regions, and hence, promoter regions are classified into two distinct types, CpG-island and non-CpG-island promoters (Davuluri et al. 2001). This bimodal typing can be readily applied to mice and other vertebrates as well (Elango and Yi 2008). Alternatively, the invertebrate chordate, ascidians, shows a clear unimodal promoter type that differs from CpG islands (Okamura et al. 2011). It, therefore, remains unclear how the two promoter types in vertebrates evolved.

Considering that housekeeping genes reportedly bear a CpG island in their promoter regions, it is likely that the presence of CpG-islands is conserved among various species (Zhu et al. 2008). However, a study using 3197 human–mouse orthologous promoter pairs revealed divergence in their presence and sequences (Jiang et al. 2007). In particular, less pronounced characteristics and degeneration of mouse CpG-island promoters were observed. Moreover, our group previously sequenced the CpG island of the *Impact* gene for 27 mammalian species (Okamura et al. 2008), in which the island spanning the first exon was lost in nine rodent species. Of the nine, three murids (mouse, rat, and wood mouse) had gained a new CpG island at the 3ʹ portion of the first intron, likely via expansion of CpG-containing repeat elements, such as TCGGC. The genomic sequences for these islands are too divergent to be aligned, even among the rodents. Similarly, a genome-wide analysis of 131 mammals was performed; however, the number of eligible or available promoters in the resulting alignments was small (McLain and Faulk 2018). Thus, species divergence may limit the number of alignments obtained, as well as the subsequent comparisons for the increasing number of genomic sequences.

To conduct comparative genomics at base resolution, nucleotide sequences should be highly conserved. However, if aligned sequences share high similarity, informative output cannot be obtained. Using the human genomic sequence as a control, mouse and chimpanzee non-coding sequences are considered to be too different and too similar, respectively. Thus, the rhesus monkey genome was employed as it exhibits moderate divergence. The macaque diverged from our last common ancestor approximately 25 million years ago, and an average identity of the genomes was reported to be ~ 93% (Gibbs et al. 2007). Further, its genetic, morphological, and physiological similarity to humans makes it an important model animal in biomedical research.

Since promoters are non-coding, sequence alignment is not as straightforward as with protein-coding sequences, which consist of anchored 3-bp frames. Therefore, we employed transcription start sites (TSSs) as an alternative anchor. TSSs can be determined by the Oligo-capping method or Cap Analysis Gene Expression (CAGE), both of which exploit the 5ʹ cap of mRNA molecules (Shiraki et al. 2003). In the former method, the cap structure is treated with tobacco acid pyrophosphatase to allow for its substitution with a specific RNA oligonucleotide without causing undesirable sequence changes (Maruyama and Sugano 1994). As seen in RNA-seq, the number of clones correlated well with transcriptional quantity (Yamashita et al. 2005). For mice and humans, TSS data have been compiled in a database, which was employed in the current study to perform a comparative analysis of human and macaque promoters at base resolution to identify factors driving diversification of gene regulatory sequences in mammals.

## Results

### Employment of Transcription Start Site Data

**Fig. 1:**
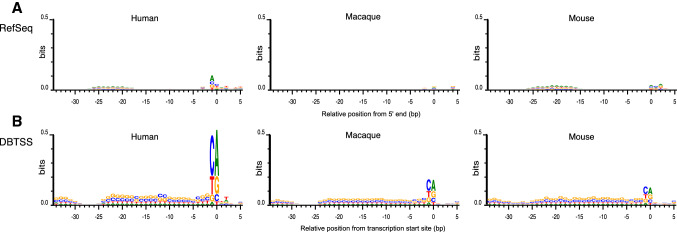
Sequence logos obtained from 40-nt promoter sequences. Human, macaque, and mouse logos are displayed from left to right. Transcription start sites (TSSs) are located at position 0, which corresponds to the traditional + 1 start site. **a** TSSs were inferred from 5ʹ end of RefSeq entries. Promoter elements are not clear in all the three species. **b** Human representative TSSs were obtained from DBTSS. The Inr or YR consensus dinucleotide is evident. Disappearance of G and C nucleotides around position -27 suggests presence of TATA boxes. Macaque and mouse TSSs were inferred from sequence alignments against human TSSs

Although CpG islands are often associated with gene promoters (Bird 1987), more than half are likely located in non-promoter regions in the human genome (Takai and Jones 2003). Using data for transcription start sites (TSSs), we first identified CpG islands that functioned as promoters. We assumed that CpG-island promoters bear at least one TSS in their genomic sequences. TSS of each gene can be readily obtained as the first position of curated cDNA sequences registered in NCBI RefSeq (Pruitt et al. 2014). For comparison, we also evaluated DataBase of Transcriptional Start Sites (DBTSS), in which each site was supported by experimentally determined positions of 5ʹ cap structures (Wakaguri et al. 2008). Considering base resolution positions and transcriptional directions, 40-nt promoter sequences, 34 nt upstream and 5 nt downstream flanking regions from a TSS, were excised from the human reference sequence GRCh37. The datasets consisting of 60,120 and 16,911 sequences from RefSeq and DBTSS, respectively, were prepared independent of the presence of CpG islands. In contrast to the sequence logo obtained from the former set, the latter clearly exhibited the initiator (Inr) motif YANW (Fig. 1) (Juven-Gershon et al. 2008). Here, degenerated nucleotides are indicated according to the IUPAC code. The pyrimidine-purine (YR) consensus was particularly prominent when the DBTSS data were used to excise the 40-nt promoter sequences. The A or R nucleotide in the sequence motifs represents the transcription start point. Since the former alignment failed to detect the Inr motif and the dinucleotide consensus, it is unlikely that the 5ʹ ends of many RefSeq entries represent *bona fide* TSSs. Hence, we employed DBTSS to determine positions and directions of promoters, as well as to decipher whether a CpG island functioned as a promoter.

### Divergence of Human and Mouse Promoter Sequences

Generally, one gene locus has many TSSs, which form a cluster around a core promoter. Considering frequency of usage and genomic position, a single TSS is selected as a representative for each core promoter in DBTSS (Wakaguri et al. 2008). For each human representative TSS, the flanking 300-nt sequences from both upstream and downstream regions were excised from the reference sequence, taking into account the transcriptional direction, to form a set of 601-nt promoter sequences, ultimately generating 20,612 promoter sequences. Of these sequences, 16,911 were associated with RefSeq accession prefix NM, *i.e.*, curated model of protein-coding genes. Using these 16,911 promoter sequences as queries, we searched the mouse reference sequence GRCm38 for orthologous promoters that are relatively conserved between the two species. BLAT using default settings listed 8601 hits. We then eliminated the sequences with a mismatch at the YR consensus dinucleotide between humans and mice. Applying this careful screening, 2739 promoter sequences were obtained, which was comparable (3197) to that reported in a previous study (Jiang et al. 2007). Of these, 336 predicted TSSs coincided with the mouse data deposited in DBTSS. This approach missed most mouse promoters, suggesting a high degree of sequence divergence between the human and mouse promoters. Although a small number was anticipated, it was too small to perform comparative genome analyses. As another possible model organism, the rhesus monkey, *Macaca mulatta*, was selected, which is expected to have moderate sequence conservation and moderate divergence compared to the human genome (Yan et al. 2011; Zimin et al. 2014). While 5833 transcripts have been released as UCSC refGene for the macaque, 16,662 hits and 12,598 highly possible promoters were obtained by the BLAT alignment.

In addition to human data, sequence logos of mouse and macaque data were drawn (Fig. 1). For the latter two species, TSSs were inferred from human representative TSSs compiled in DBTSS. Clear YR consensus sequences were obtained, suggesting this method to effectively predict TSSs for organisms without experimentally determined TSSs. Those promoter sequences, however, should be conserved to some extent, *e.g.,* desirably to the degree seen between humans and macaque.

**Fig. 2:**
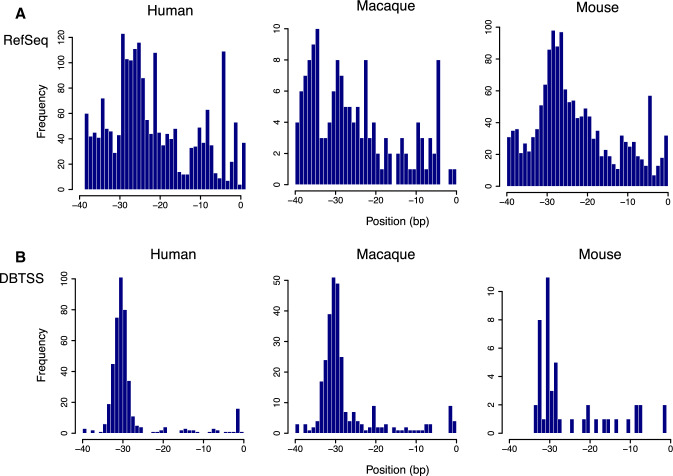
Genomic locations of possible TATA boxes relative to TSSs. **a** Histograms depict distributions of 5′-TATAAA-3′ fragments in promoters. Position 0 corresponds to 5ʹ end of RefSeq entries. **b** When DBTSS was employed, a sharp peak was observed around position − 31 in each species. In mice, the number of inferred start sites was not large due to a high degree of sequence divergence from humans

**Fig. 3:**
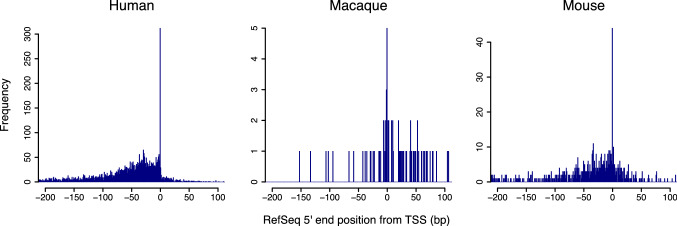
Locational difference of transcription start sites between RefSeq and DBTSS. Histograms indicate relative positions of RefSeq 5ʹ ends compared to TSS data from DBTSS. RefSeq 5′ ends tend to be positioned downstream from TSSs. As for macaque, available RefSeq entries were limited compared to human and mouse

The upstream regions from the TSSs appeared to be G + C-rich for the three species, although CpG-island promoters were not selected in this analysis (Fig. 1). Interestingly, from position − 25 to − 29, enrichment of G and C was lost for all species. Because the position corresponds to that of the TATA box (Juven-Gershon et al. 2008), we searched the upstream regions for its consensus sequence (Fig. 2). To avoid TA dinucleotide repeats, a 6-nt sequence TATAAA was used as a query in this search. In the histogram showing distribution of the consensus sequence, the most frequent position was at position − 31, which was clearly observed when DBTSS data were employed. TSS positional coincidence between RefSeq and DBTSS data was observed in only 312 genes out of 13,772 human transcripts, in which TSS data were available in both databases. In more than 90% of the gene loci, RefSeq start positions were explicitly situated upstream from the corresponding DBTSS data (Fig. 3). These results suggest an instrumental feature for DBTSS to process promoter sequences. Therefore, we employed the DBTSS data for further analyses.

### Definition of a CpG-Island Promoter

Several definitions of a CpG island have been proposed (Gardiner-Garden and Frommer 1987; Takai and Jones 2002; Wu et al. 2010). While the existence of G + C-rich *Alu* sequences in primate genomes tends to complicate the definition, the role of *Alu* in gene expression is generally considered to be insignificant. Accordingly, they were shown to be absent in the vicinity of TSSs (Yamashita et al. 2005). Hence, we used 300-nt flanking sequences to form 601-nt promoter sequences, which fully covered the 500-bp core portion of a CpG island, and defined the sequence as a CpG-island promoter if the G + C content was greater than 0.5, and the CpG score was greater than 0.6. Here, the ratio of observed over expected CpG numbers represented the CpG score, as in the UCSC database. Any 601-nt promoter sequences that failed to meet these criteria were deemed non-CpG-island promoters.

**Fig. 4:**
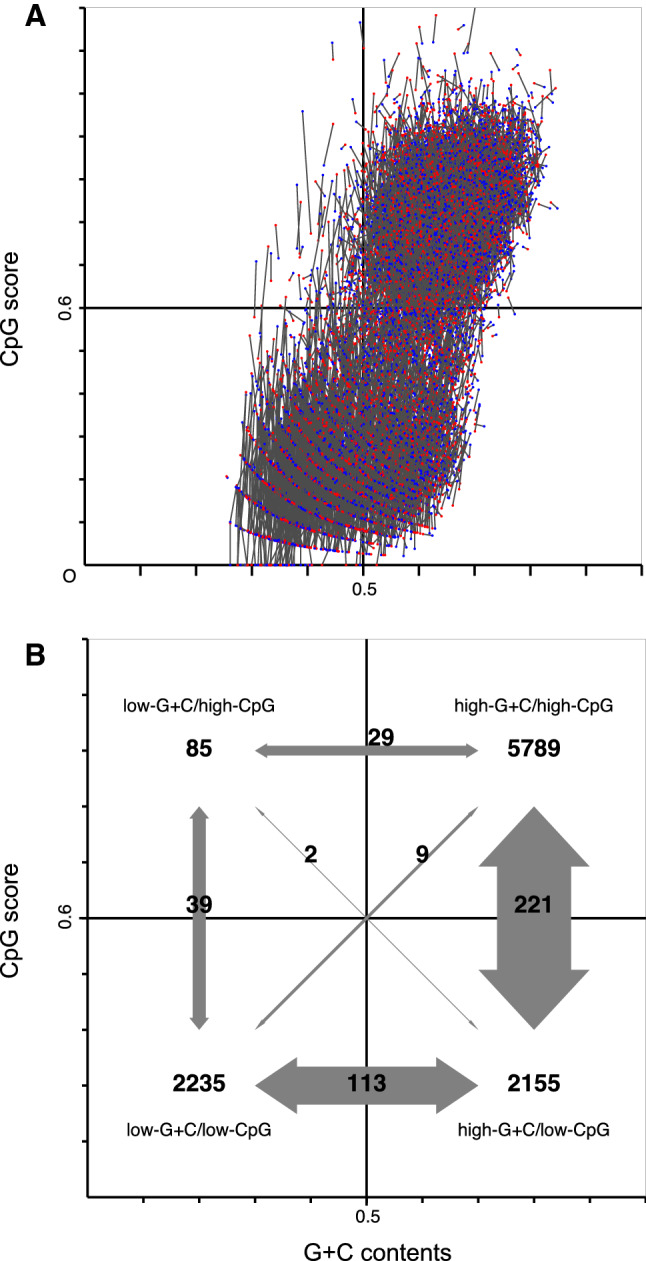
Scatter plot of aligned human and macaque promoter pairs. Horizontal and vertical axes represent G + C content and CpG score, respectively. The vertical 0.5 and horizontal 0.6 lines split the promoters into four groups. The top right group corresponds to CpG-island promoters; the other three groups correspond to non-CpG-island ones. **a** Each line connecting human (blue dot) and macaque (red dot) promoters represents a change in the contents and scores between the two species. Most connecting lines were short, indicating the contents and scores are highly conserved between human and macaque. A scalable version of this scatter plot is also provided (supplementary Fig. S1). **b** Numbers representing change in promoter types between humans and macaque. The numbers were obtained by counting the connecting lines that span the group border and are shown with gray arrows. Width of the gray arrow represents number of changes between two groups. CpG score changes in high-G + C content were predominant. Intriguingly, almost all invertebrate ascidian promoters clustered in the top left group

Changes in G + C content and CpG scores between the human and macaque promoters are illustrated as a scatter plot (Fig. 4, Supplementary Fig. S1). Orthologous counterparts were connected by a single line, while vertical and horizontal lines were drawn to discern the two promoter types. CpG-island promoters formed an apparent cluster in the top right region, in which G + C content and CpG score were more than 0.5 and 0.6, respectively. In addition, the two lines partitioned the plot into four regions, namely, low-G + C/high-CpG (left top), high-G + C/high-CpG (right top), low-G + C/low-CpG (left bottom), and high-G + C/low-CpG (right bottom). The majority of promoter pairs were located in the second region, CpG-island promoters. Others were principally located in the two bottom regions or low-CpG promoters. Appearance of low-G + C/high-CpG promoters was exceptional in humans and macaque. Large numbers of changes were observed between high-G + C/high-CpG and high-G + C/low-CpG (right top–bottom), as well as between low-G + C/low-CpG and high-G + C/low-CpG (right–left bottom). Connecting lines that span a border of two promoter groups were counted and illustrated (Fig. 4). It is likely that G + C contents and CpG scores have drifted evolutionarily in either of the following two ways: changes in G + C content in low-CpG genomic context, or CpG content in high-G + C genomic context. Performing a gene ontology analysis revealed that such liable promoters were preferentially found in genes associated with alternative splicing (150 of 258 hCmN and 99 of 158 hNmC).

**Fig. 5:**
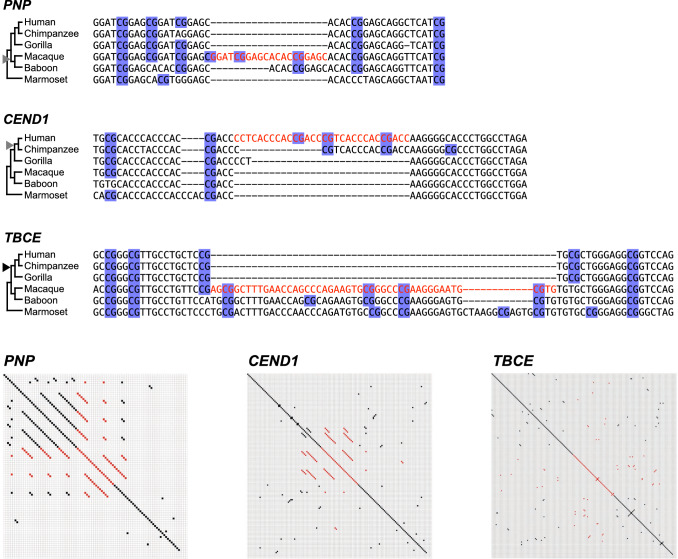
Examples of indels in human and macaque promoters. Sequence alignments are shown for the *PNP*, *CEND1*, and *TBCE* promoters, in which an indel altered the promoter types between the two species. Harr plots were drawn to detect tandem repeats related to insertion or deletion events (rheMac8 chr7:81,686,561–81,686,600 for *PNP*, hg19 chr11:790,324–790,373 for *CEND1*, and rheMac8 chr1:211,651,491–211,651,557 for *TBCE*). Each dot indicates a 5-base perfect match. Indel-related sequences are shown in red. Multiple alignments of the six primate species were drawn to determine the type of mutation event, namely insertion or deletion. The gray and black triangles indicate the occurrence of an insertion and deletion, respectively. CpG islands were gained in macaque *PNP* and human *CEND1* through insertion and lost in hominoid *TBCE* via deletions. All CpG sites are shaded in blue

The number of 601-nt promoter sequences detected in the macaque genome was 12,543, which were then realigned to the corresponding human queries with ClustalW 2.1 to examine base changes between the two species. Pairwise alignments with alignment scores less than 90 were discarded from further analysis. Additionally, those with a mismatch in the YR consensus dinucleotide were also discarded. More than 97% of promoter pairs between human and macaque preserved their promoter types, *i.e.*, CpG-island or non-CpG-island promoters (Table 1). However, the *TBCE* gene represents one of the rare cases that showed a discrepancy of promoter types (Fig. 5). Although the orthologous promoter sequences were highly similar between the two species, a major difference was a 47-bp indel near the 3ʹ end. BLAT search for the 47-bp fragment using chimpanzee, gorilla, baboon, and marmoset reference sequences revealed that a deletion event may have occurred in a common ancestor of gorilla, chimpanzee, and humans. Compared to the macaque sequence, four CpG sites were lost by the deletion in the human *TBCE* promoter, resulting in transition from a CpG-island promoter to a non-CpG-island promoter in hominids, namely great apes.

**Table 1:** Classification of pairwise-aligned promoters

		rheMac8 (macaque)			mm10 (mouse)		
		CpG-island promoter	Non-CpG-island promoter	Subtotal	CpG-island promoter	Non-CpG-island promoter	Subtotal
hg19	CpG-island promoter	6893	259	7152	1557	167	1724
(human)	Non-CpG-island promoter	158	5288	5446	47	968	1015
	Subtotal	7051	5547	12598	1604	1135	2739

### CpG Mutation Spectrum

**Fig. 6:**
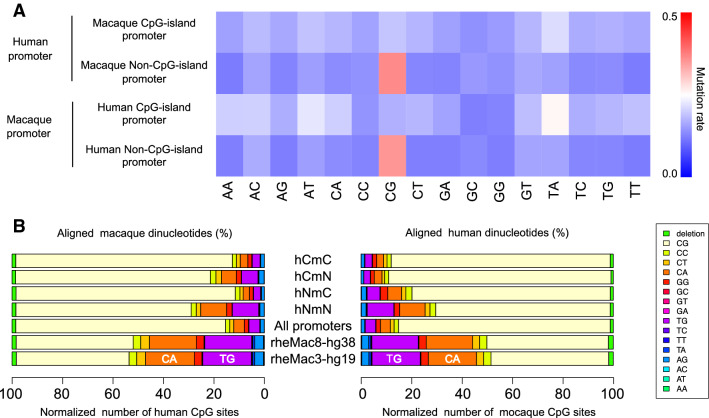
Dinucleotide alterations between human and macaque promoters. **a** Heat map showing alteration rates of each dinucleotide. Increased rates were observed when corresponding dinucleotides were not conserved between the two species. In the top two rows, human promoters were aligned to macaque CpG-island and non-CpG-island promoters, respectively. In the bottom two rows, macaque promoters were aligned to human CpG-island and non-CpG-island promoters, respectively. Both single-nucleotide substitutions and indels were considered for the rate calculation. Frequent alteration of CpG sites was observed when a promoter aligned to a non-CpG-island promoter of the counter species. Alteration of TpA dinucleotide was also high when aligned to a CpG-island promoter of the counter species. **b** Band graphs indicate alignment of dinucleotides to CpG sites. Between human and macaque CpG-island promoters, CpG sites were well aligned to CpG sites in the counter species (top bands). In background genomic sequences, however, many CpG sites were aligned to TpG or CpA sites (bottom bands). *hCmC* human CpG-island and macaque CpG-island promoter pairs, *hNmN* human non-CpG-island and macaque non-CpG-island promoter pairs

In vertebrate genomes, CpG sites are subject to cytosine methylation, often followed by deamination and mutation to TpG (or CpA if deamination occurred in the complementary strand). In the human genome, the frequency of the CpG dinucleotide was extremely low among all 16 dinucleotide sequences. Conversely, frequencies of resultant TpG and CpA were the highest, except for A-rich or T-rich ones, *i.e.*, ApA, ApT, TpA, and TpT (Okamura et al. 2007). We then examined alteration rates of the 16 types of dinucleotides between the two species (Fig. 6) and found the most frequent alteration to be in the CpG dinucleotide aligned to the non-CpG-island promoter of the counter species. In contrast, CpG sites in CpG-island promoters were relatively conserved between the human and macaque genomes. Further, many alterations in TpA dinucleotides were observed if aligned to CpG-island promoters of the counter species.

As for CpG sites in CpG-island promoters, more than 80% of the dinucleotide sequences were conserved between the two species. In particular, 85.7% and 86.7% of the CpG sites in human and macaque CpG-island promoters, respectively, were aligned without mismatch (Fig. 6, Supplementary Table S2). The slight difference in these percentage values arose as the total number of CpG sites between the counterparts was not identical. However, dinucleotide conservation was not observed in non-CpG-island promoters. In type-change promoter pairs, a large number of CpG-to-TpG/CpA transitions were found. The dinucleotide changes were observed 2.8 and 3.5 times more frequently in humans and macaque, respectively, than those in CpG-island promoters. The frequencies were also higher than those in background genomic sequences. Thus, it is likely that deamination mutations contributed to promoter variation.

### Characterization of Inserted and Deleted Sequences in Promoters

**Fig. 7:**
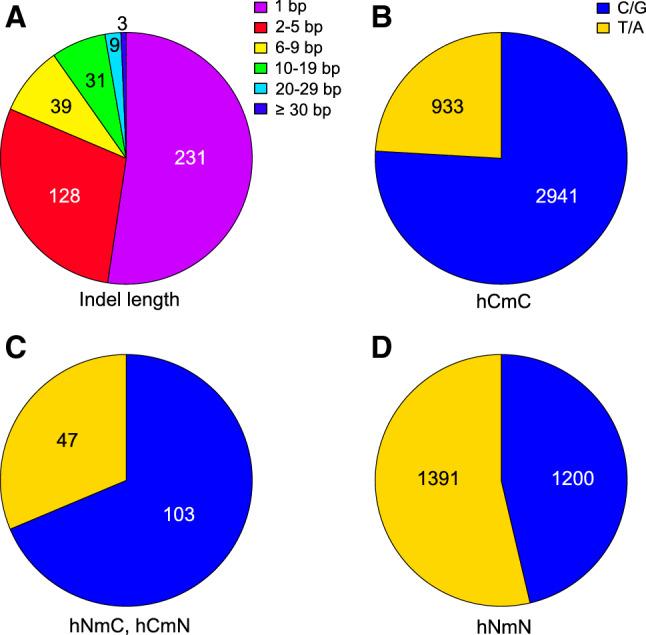
Pie charts showing number of insertions or deletions between human and macaque promoters. **a** Number of detected indel sites is classified by length. More than half were single-nucleotide insertions or deletions. The longest indel detected was 35 bp. **b–d** Types of nucleotides detected in alignments are shown between CpG-island promoters (**b**), CpG-island and non-CpG-island promoters (**c**), and non-CpG-island promoters (**d**). In CpG-island promoters, inserted or deleted nucleotides were biased toward C or G. *hCmC* CpG-island promoter in human and macaque, *hNmC* CpG-island promoter in macaque and not in human, *hCmN* CpG-island promoter in human and not in macaque, and *hNmN* non-CpG-island promoters in the two species

While most of the human–macaque promoter pairs consisted of the same type between the two species, *i.e.*, CpG-island or non-CpG-island type, 417 pairs (158 pairs + 259 pairs) showed discordance (Table 1). In addition to nucleotide substitutions, many insertions and deletions were detected in their sequence alignments, suggesting that such events could have evolutionarily altered the promoter types as shown in the *TBCE* locus. Additionally, multiple sequence alignments of the *PNP* and *CEND1* promoters are provided as examples of insertion by repeat expansion (Fig. 5). From all of the indels, we selected 439 sites whose neighboring 10-bp regions on both sides showed 90%, or greater, similarity between human and macaque genomic sequences. Approximately 10% were 10-bp or longer indels (Fig. 7). Alterations in the numbers of CpG dinucleotides, including in the nearby regions, were observed in 82 indel sites. Among these, only 16 sites showed two or more CpG-site alterations, including six sites harboring a 20-bp, or longer, indel. In CpG-island promoters, long indels often contained several CpG sites. If an inserted or deleted fragment contained CpG sites, the event could contribute to gain or loss of CpG-island-like sequences. For all the detected indel sequences longer than 4 nt, Harr plots were drawn along with neighboring sequences (Fig. 5, Supplementary Table S1, Supplementary Fig. S2). Of all 88 indels, 77 were associated with tandem repeats, including 16 indels with inverted repeats (Supplementary Fig. S2). In total, repetitive sequences were found in 365 out of the 439 indel sites, including shorter indels (Supplementary Table S3). More than 50% of the indels were single-nucleotide insertions or deletions (Fig. 7). In many cases, they could be considered to be an extension or shortening of homopolymeric repeats. Inserted or deleted single nucleotides clearly depended on promoter types; while nucleotide bias was not observed in non-CpG-island promoters, frequent C or G indels were identified in CpG-island promoters.

## Discussion

Although the rhesus monkey is an instrumental model organism for understanding many aspects of humans, the number of documented genes or transcripts in these monkeys is much smaller than in humans and mice. Further, genomic annotation of macaque has been limited. Therefore, curated cDNA sequences, such as datasets provided by NCBI RefSeq, have been used to infer putative promoter regions (Pruitt et al. 2014). In this study, we exploited a TSS database and moderate similarity between human and macaque genomic sequences to compile a list of putative promoter regions in the macaque genome and performed a comparative analysis to assess factors underlying promoter diversity.

We found that 5ʹ ends of curated cDNA sequences tend to be positioned far upstream of TSSs determined by the Oligo-capping method (Maruyama and Sugano 1994). Moreover, the 5ʹ end positions of the RefSeq entries were not supported by widely studied promoter elements such as TATA box and Inr, which may be due to extending the 5ʹ end of cDNA fragments further upstream to obtain full lengths of the corresponding transcript (Harrow et al. 2006). In general, TSSs are dispersed throughout a promoter region, at least in mammalian genomes (Okamura and Nakai 2008). The longer the transcript that is being detected, the more rarely its 5ʹ end will be used as a start site. Since DBTSS places focus on the frequency of use of each starting site rather than the position in a gene locus, more plausible promoter positions would be obtained in the macaque genome and applied to genomes of other organisms. Using affluent human annotation data, we compiled a significant number of macaque promoters. Further, our method was validated in the mouse genome, for which DBTSS is available. The obtained number of mouse promoters was smaller than that of macaque, suggesting that the method is suitable for species that show relatively high sequence similarity. However, considerations should be made regarding trimming of promoter regions using a transcript dataset. These results suggest that transcript curators should consider modifying their policy regarding 5ʹ ends.

The two representative mammalian species show high sequence conservation in protein-coding regions. However, it is quite difficult to align non-coding sequences, including regulatory regions such as promoters. Furthermore, the presence of CpG-island promoters is not conserved for many human–mouse gene loci (Jiang et al. 2007), possibly due to active gain and loss in mammals. To elucidate the mechanism, human and macaque promoters were compared. We found that G + C contents tended to change in low-CpG sequence environments, and CpG scores under high-G + C environments. Many such promoters were found in gene loci associated with alternative splicing, which is a molecular process that generates variation in the transcriptome. Additionally, few primate promoters were grouped into low-G + C/high-CpG (left top), although nearly all promoters were classified as this type in the invertebrate, ascidian (Okamura et al. 2011). This suggests that the advent of vertebrate CpG-island promoters cannot be simply explained by examining diversification of mammalian promoters.

Despite moderate sequence divergence, changes were detected in 417 promoters, most of which were C-to-T transitions. Using outgroup species, multiple studies reported loss of CpG as much more frequent than changes of other dinucleotides to CpG (Jiang et al. 2007). However, without gain of CpG islands, they would have disappeared from mammalian genomes. In this study, multiple observations supported possible causes for this gain. First, many indel sequences, including CpG, were detected in promoter alignments. Because repetitive sequences were also detected around them, expansion of CpG-containing repeats could contribute to gain of CpG-islands, as in the cases of the *PNP* and *CEND1* promoters (Fig. 5). Second, compared to single-nucleotide substitutions, insertions and deletions could significantly impact divergence of genomic sequences. Further, many single-nucleotide indels were detected in promoter sequences. While protein-coding sequences are restricted to 3-nt reading frames, promoters can tolerate insertions and deletions. Therefore, non-CpG-island promoters might increase their G + C content through insertions or deletions, subsequently increasing the CpG score to become a CpG-island promoter. Base resolution comparison of other species can provide further insights into the molecular evolution.

Certain limitations were noted in the current study. For instance, this study was designed under the presumption that the human and macaque genomic sequences can be readily aligned at base resolution; therefore, a bias may have occurred toward highly conserved promoters. Although up to 35-bp indels were identified in our alignment settings, it is difficult to detect much larger structural alterations, such as deletion, segmental duplication, retrotransposition, or gene conversion, within the promoter regions. These alternate mutational events could also provide opportunities for gain or loss of a CpG island. By using orthologous gene pairs, highly diverged promoters may also be compared. Nonetheless, our study presents distinctive insights into the evolution of CpG density, which likely contributed to the fine-tuning of gene expression from early vertebrates to primates. Understanding this process may contribute to the development of platforms capable of altering gene regulation at our own convenience in the future.

## Methods

### Determination of Macaque Promoter Regions

Human promoter sequences were used to locate macaque promoter regions at their orthologous regions. Using DBTSS (https://dbtss.hgc.jp/) ver. 6, each human promoter sequence was prepared as a 601-bp fragment bearing the transcription start site (TSS) at the center and aligned to the UCSC rheMac8 assembly (Baylor College of Medicine Genome Sequencing Center Mmul_8.0.1) considering the transcriptional orientation. Meanwhile, the UCSC hg19 (GRCh37) assembly was used for the human reference genome. BLAT ver. 36, using rheMac8 as the reference, was employed for identification. Soft-masked bases in rheMac8 were hard-masked or replaced with Ns as primate genomes contain G + C-rich *Alu* sequences. Macaque sequence hits with ≥ 90% identity of over 200 bp were adopted as promoter candidates. When several fragments were obtained, the longest was selected. If a YR consensus dinucleotide was present corresponding to humans, the candidate fragment was extended to 601 bp centering the dinucleotide, and the macaque promoter underwent further analyses. Approximately 10% of candidates were eliminated due to lack of a YR dinucleotide. Fragments that could not be extended to the fixed length due to contig gaps were also eliminated. Mouse promoter sequences were located using the above-mentioned approach with the UCSC mm10 (GRCm38) assembly sequences.

### Graphical Representation of Promoter Elements

To confirm the existence and positions of promoter elements, including the Inr and TATA boxes, aligned genomic sequences were graphically represented. Sequence logos spanning 35 bp upstream and 5 bp downstream regions from TSSs were drawn using WebLogo 3.5.0 (Crooks et al. 2004). The Inr and TATA boxes were recognized by the YR consensus dinucleotide and lack of G and C nucleotides, respectively. To examine the positional distribution of TATA boxes in the promoter regions, we counted the 5ʹ-end position of only TATAAA sequences identified within the 40-bp upstream regions from TSSs.

### Inspection of 5ʹ-End Positions of RefSeq Entries

TSS positions can be inferred from 5ʹ-end positions of gene model sequences such as NCBI RefSeq. For comparison to DBTSS data, separated distances were calculated and represented in histograms. The distance was obtained by subtracting the DBTSS genomic position from that of RefSeq. Minus distance values indicated a RefSeq position upstream of the DBTSS position. Since genes curated in RefSeq have many transcript variants with different 5ʹ-end positions, only the nearest 5ʹ-end position to the corresponding DBTSS position was selected for each gene. As for DBTSS positions, TSSs supported by less than three clones were eliminated.

### Definition of a CpG-Island Promoter

A CpG-island promoter was defined as a genomic region based on the following criteria: (1) CpG score calculated as observed number of CpGs in sequence length of *N* divided by the expected number of CpG of 0.6 or more and (2) G + C content calculated as the number of C or G divided by sequence length *N* of 0.5 or more. The conventional definition was applied (Gardiner-Garden and Frommer 1987), with slight modification (length *N* was fixed as 601 bp in this study).

Orthologous pairs of promoters were grouped into the following four groups: CpG-island promoter in human and macaque (hCmC); CpG-island promoter in human and not in macaque (hCmN); CpG-island promoter in macaque and not in human (hNmC); and non-CpG-island promoters in the two species (hNmN). We used DAVID 6.8 to perform the gene ontology analysis (Huang et al. 2009).

### Sequence Alignments to Detect Nucleotide Changes

After BLAT search, putative orthologous promoter sequences were meticulously aligned using ClustalW 2.1 with gap extension penalty 0.2 (Thompson et al. 1994). Promoter pairs with alignment scores less than 90 were discarded. For alteration rates of dinucleotides, inserted or deleted nucleotides, and unidentified nucleotides denoted as N, or others in the references were counted as altered sites. Pre-aligned data, UCSC rheMac3.hg19.net.axt.gz and rheMac8.hg38.net.axt.gz, were used to calculate the alteration rates in background genomic sequences. Alignment score criterion was decreased to 80 for indel detection. However, candidate pairs were eliminated if neighboring 10-bp sequences on both sides did not show 8 bp or more matches.

### Multiple Sequence Alignment of Primate PNP, CEND1, and TBCE Promoters

Using the human and macaque *PNP*, *CEND1*, and *TBCE* promoter sequences as BLAT queries, orthologous sequences of other primates were obtained. Subject reference assemblies were UCSC panTro6, gorGor5, papAnu4, and calJac3 for chimpanzee, gorilla, baboon, and marmoset, respectively. Output sequences by ClustalW were visually inspected and manually realigned, if necessary.

### Detection of Repetitive Sequences in Promoter Regions

To detect repetitive sequences in promoter regions, Harr plots were drawn with our custom scripts. We used a 5-bp sliding window with a 1-bp stride. One dot indicated a 5-bp perfect match. Except for the *PNP*, *CEND1*, and *TBCE* promoters, Harr plots are provided as supplementary material (supplementary fig. S2).

## Electronic supplementary material

Below is the link to the electronic supplementary material.

Supplementary file1 (PDF 1398 kb)

Supplementary file2 (PDF 1539 kb)

Supplementary file3 (PDF 185 kb)

## Data Availability

All data are available upon request. Custom scripts, which were written in Python, are available upon request.
