# Supplementary figures and images for: Diversification of CpG-Island Promoters Revealed by Comparative Analysis Between Human and Rhesus Monkey Genomes
Source: Mamm Genome. 2020 Jul 9;31(7):240–51. doi: 10.1007/s00335-020-09844-2 (PMC7496026; doi:10.1007/s00335-020-09844-2)

Supplementary Fig. S1

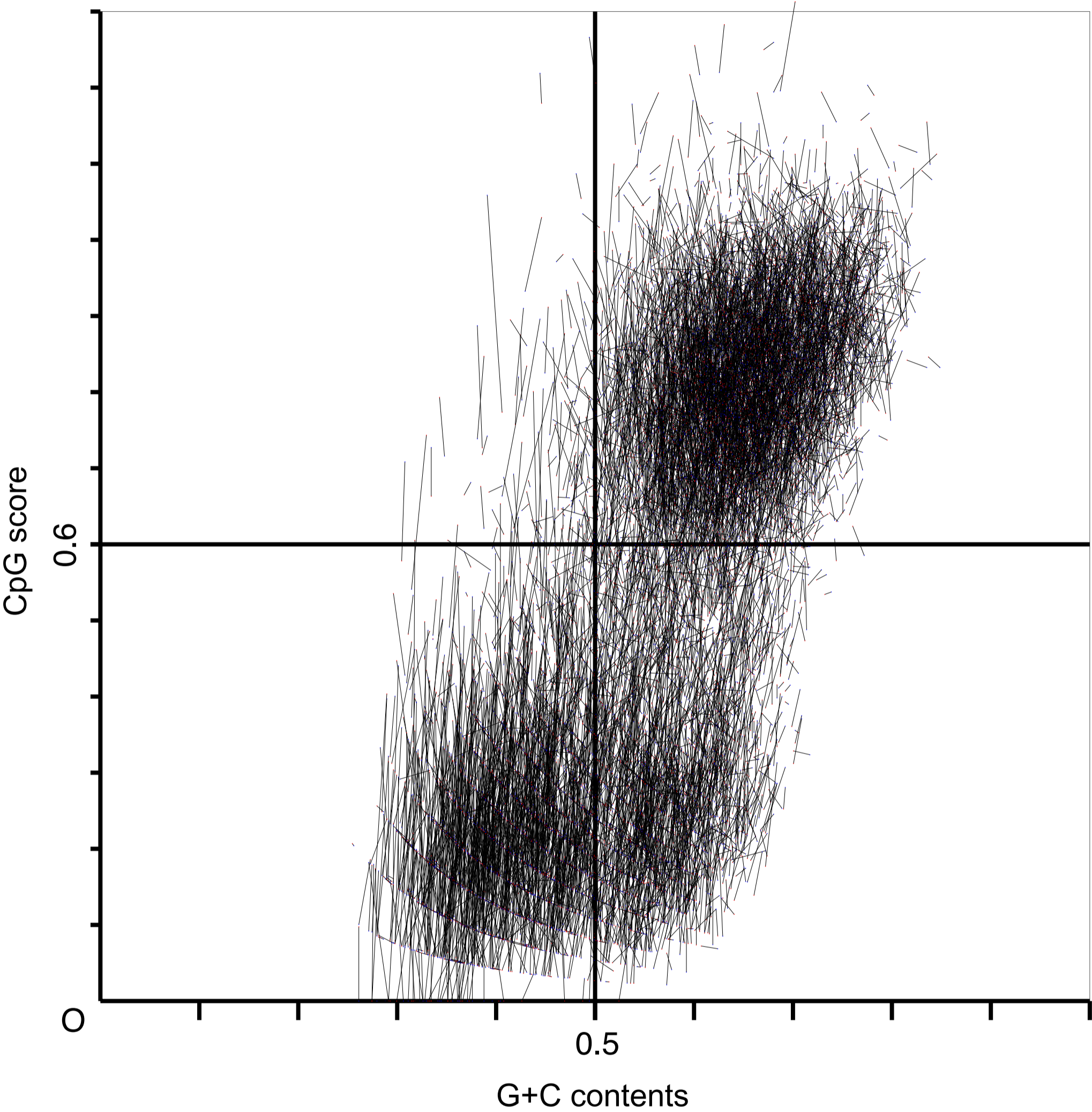

Supplement: Supplementary file 1 — Supplementary file1 (PDF 1398 kb) [file 335_2020_9844_MOESM1_ESM.pdf]
