# Supplementary material for: Diversification of CpG-Island Promoters Revealed by Comparative Analysis Between Human and Rhesus Monkey Genomes
Source: Mamm Genome. 2020 Jul 9;31(7):240–51. doi: 10.1007/s00335-020-09844-2 (PMC7496026; doi:10.1007/s00335-020-09844-2)
Supplement: Supplementary file 2 — Supplementary file2 (PDF 1539 kb) [file 335_2020_9844_MOESM2_ESM.pdf]

HP01

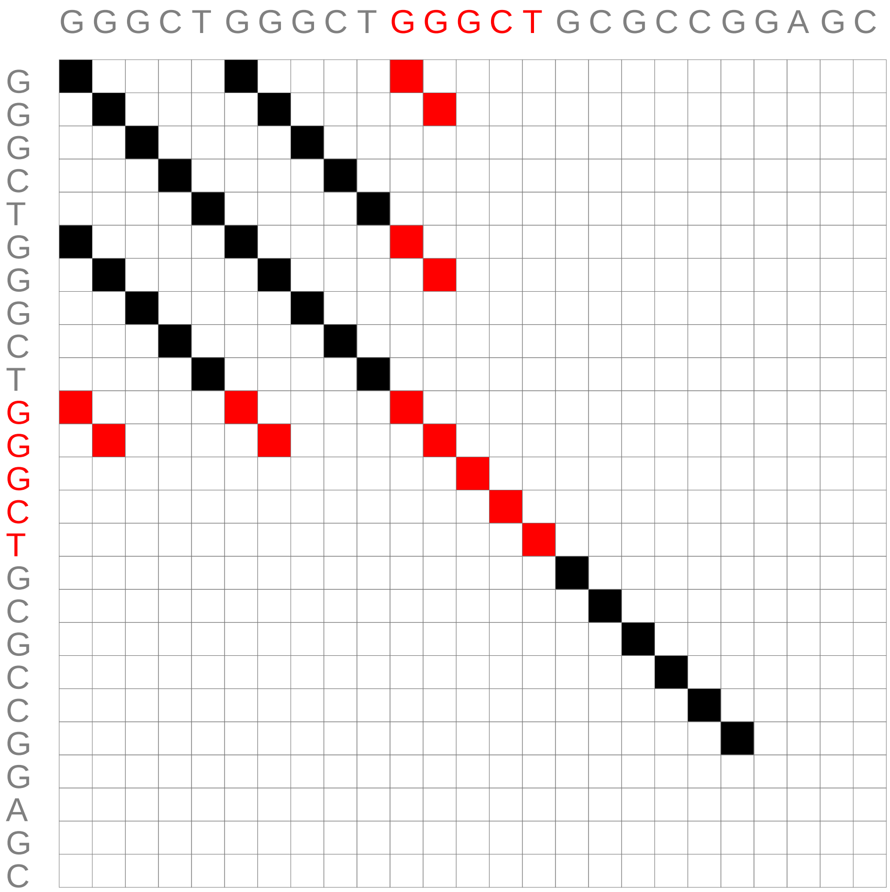
G G G C T G G G C T G G G C T G C G C C G G A G C
G G G C T G G G C T G G G C T G C G C C G G A G C

HP02

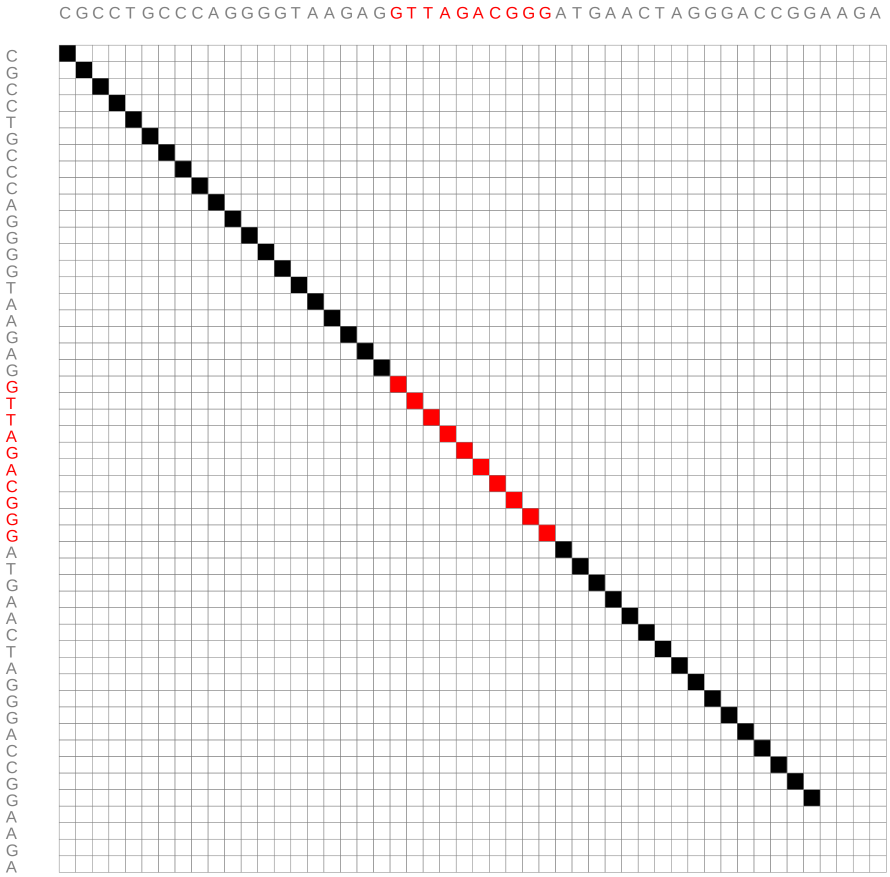
C G C C T G C C C A G G G G T A A G A G G T T A G A C G G G A T G A A C T A G G G A C C G G A A G A
C G C C T G C C C A G G G G T A A G A G G T T A G A C G G G A T G A A C T A G G G A C C G G A A G A

HP03

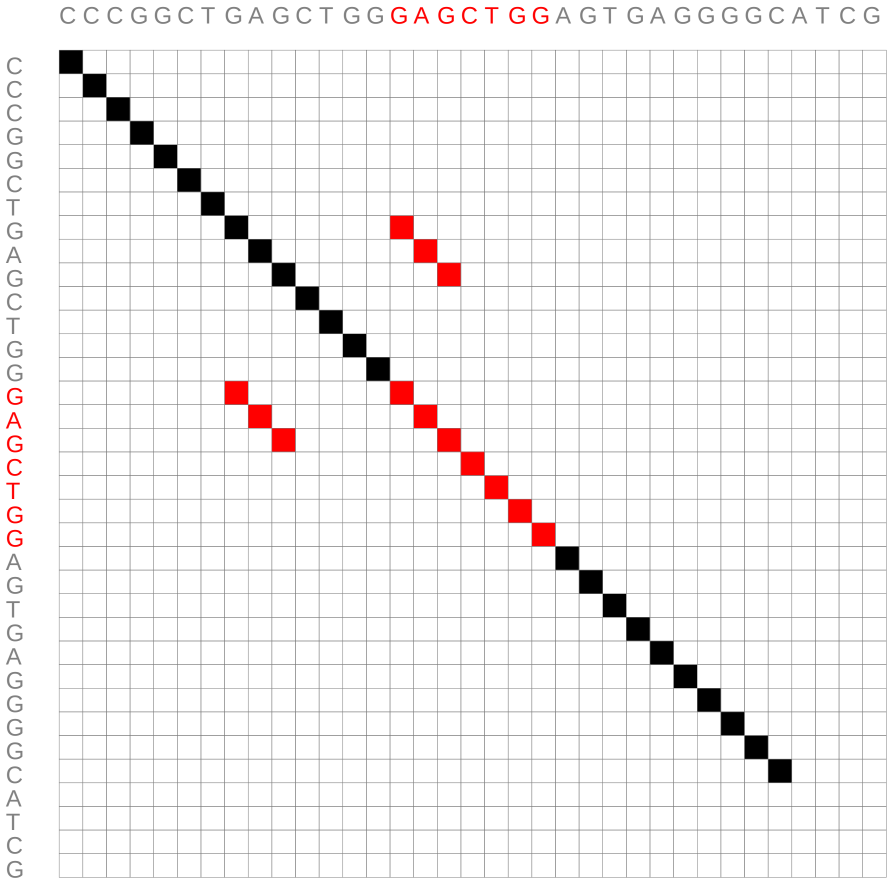
C C C G G C T G A G C T G G G A G C T G G A G T G A G G G G C A T C G
C C C G G C T G A G C T G G G A G C T G G A G T G A G G G G C A T C G

HP04

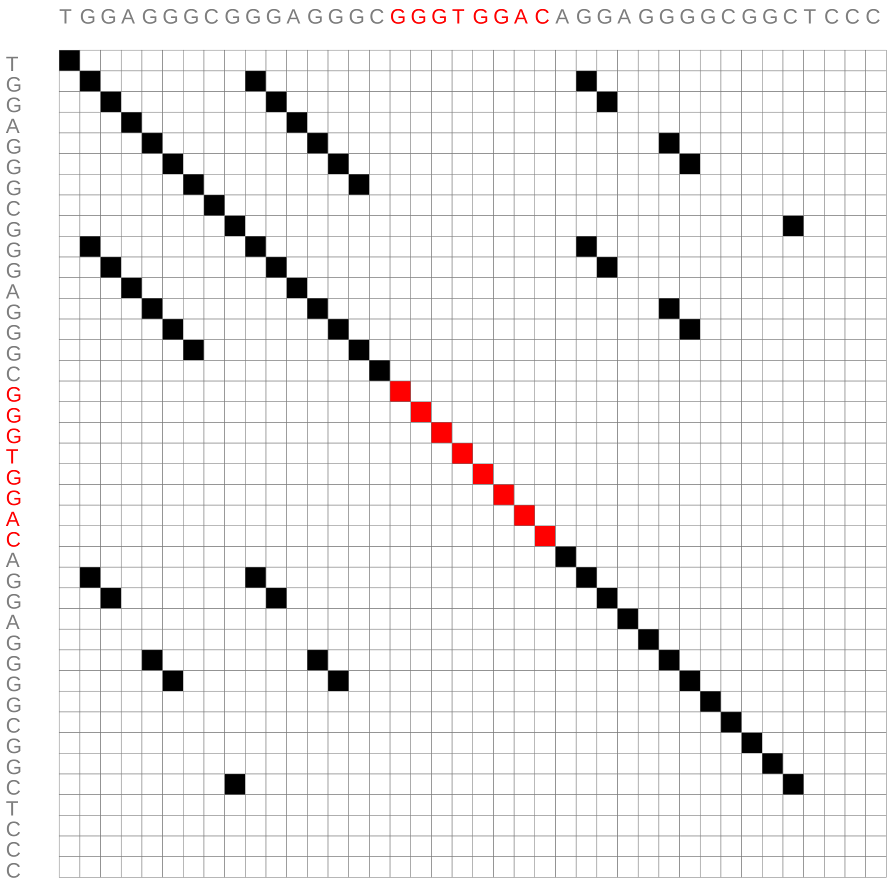

T G G A G G G C G G G A G G G C G G G T G G A C A G G A G G G G C G G C T C C C
T G G A G G G C G G G A G G G C G G G T G G A C A G G A G G G G C G G C T C C C

HP05

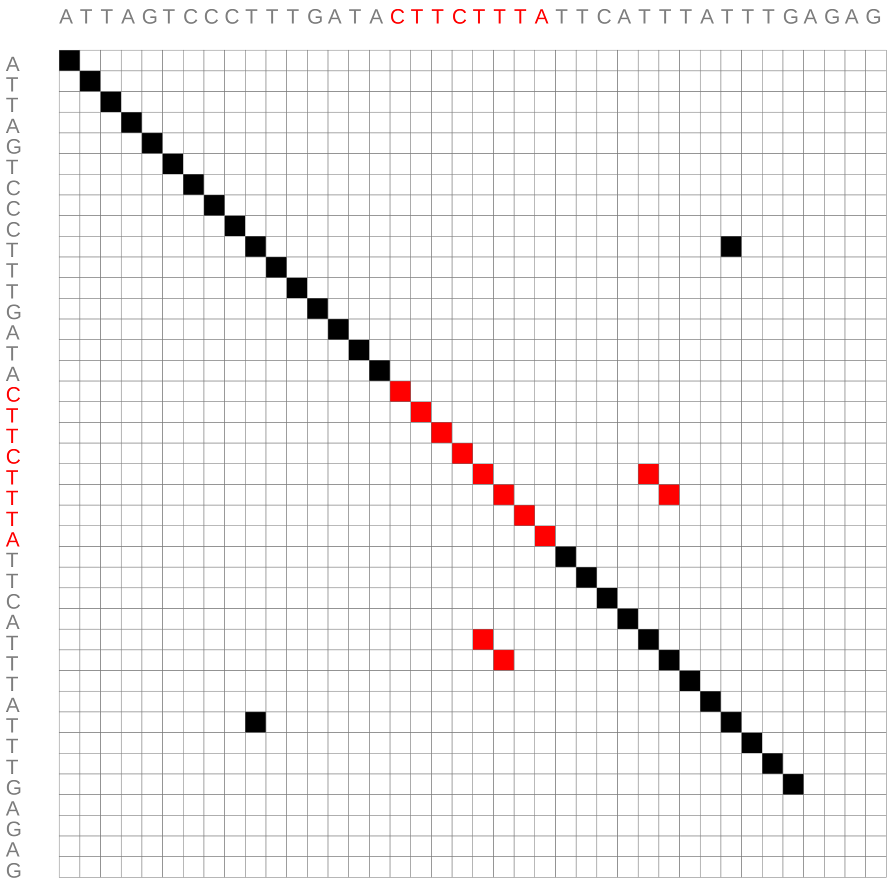
A T T A G T C C C T T T G A T A C T T C T T T A T T C A T T T A T T T G A G A G
A T T A G T C C C T T T G A T A C T T C T T T A T T C A T T T A T T T G A G A G

HP06

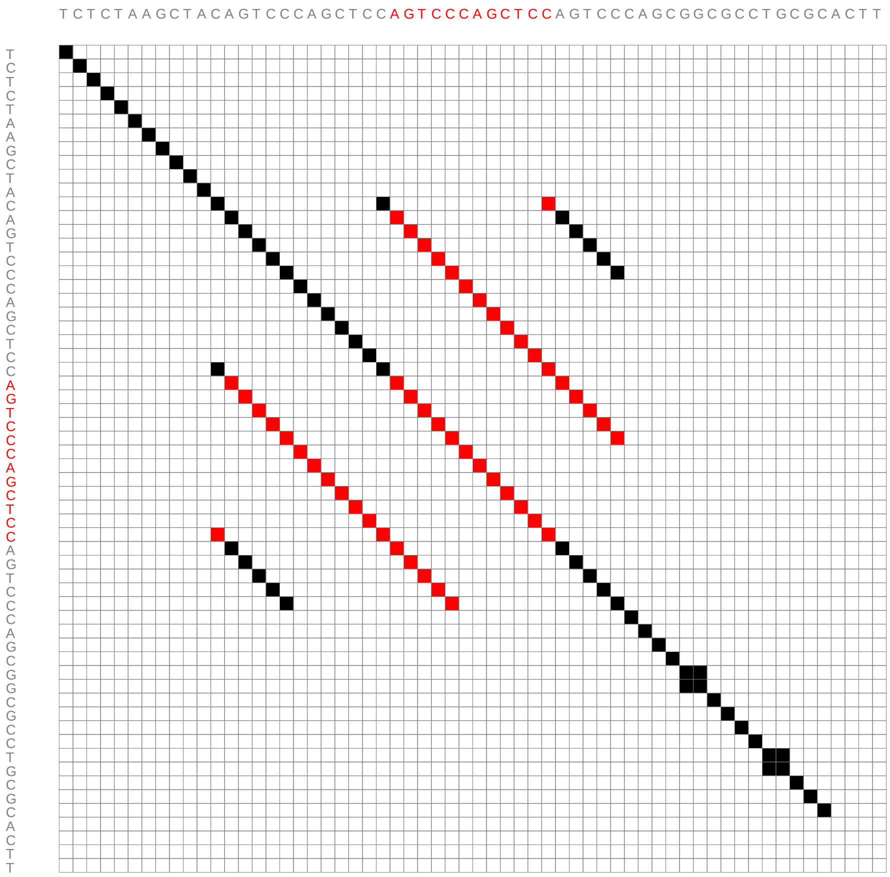
T C T C T A A G C T A C A G T C C C A G C T C C A G T C C C A G C T C C A G T C C C A G C G G C G C C T G C G C A C T T

HP07

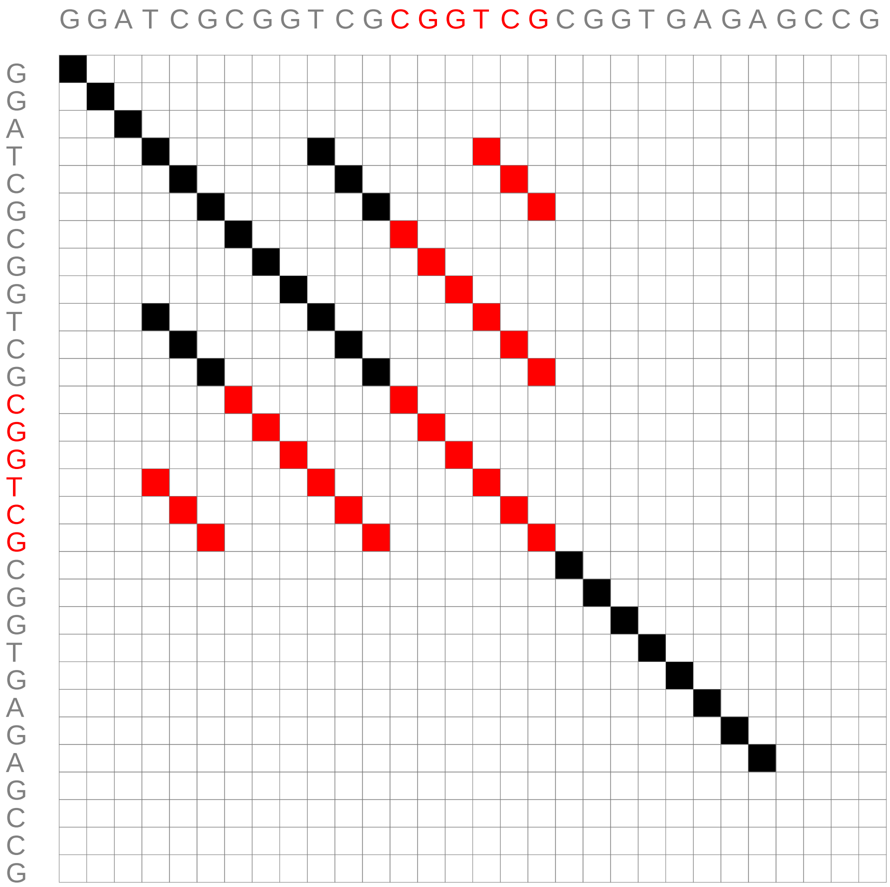
G G A T C G C G G T C G C G G T C G C G G T G A G A G C C G
G G A T C G C G G T C G C G G T C G C G G T G A G A G C C G

HP08

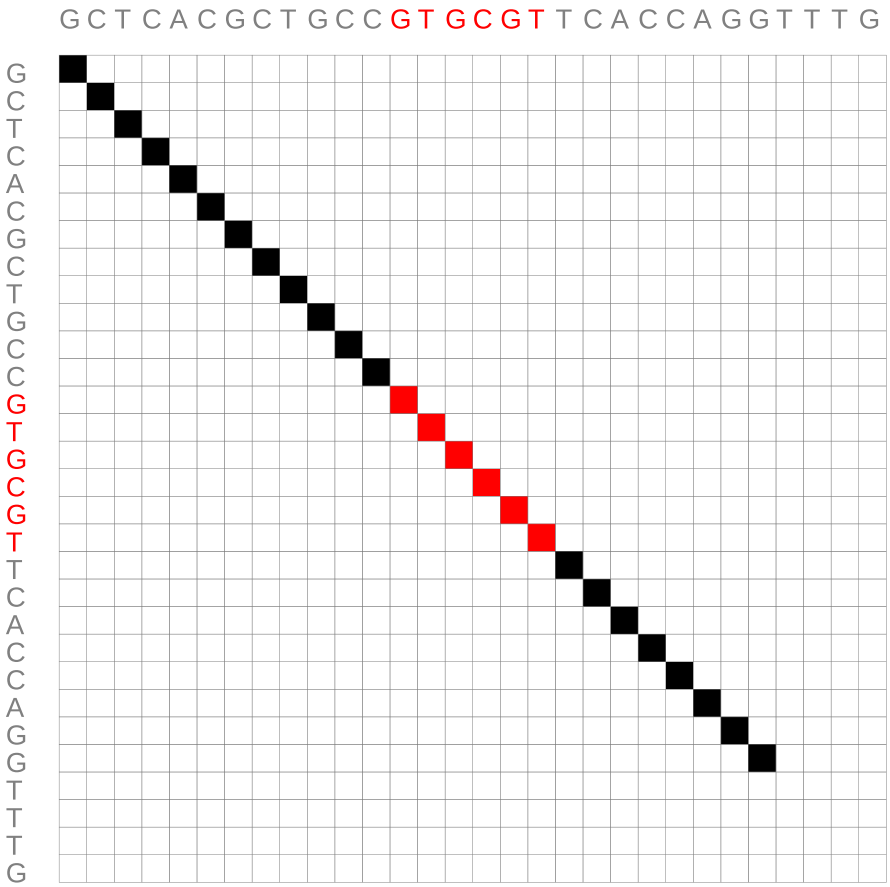
G C T C A C G C T G C C G T G C G T T C A C C A G G T T T G
G C T C A C G C T G C C G T G C G T T C A C C A G G T T T G

HP09

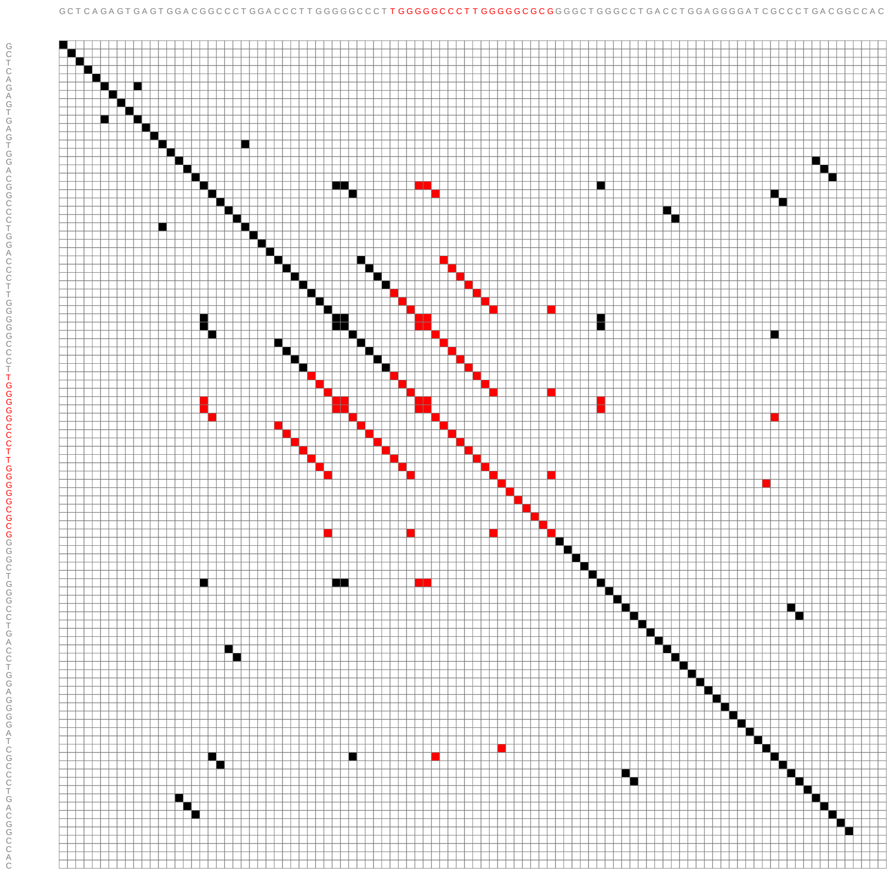
GCTCAGAGTGAGTGGACGGCCCTGGACCCTTGGGGGCCCTTGGGGGCCCTTGGGGGCGCGGGGCTGGGCCTGACCTGGAGGGGATCGCCCTGACGGCCAC

HP10

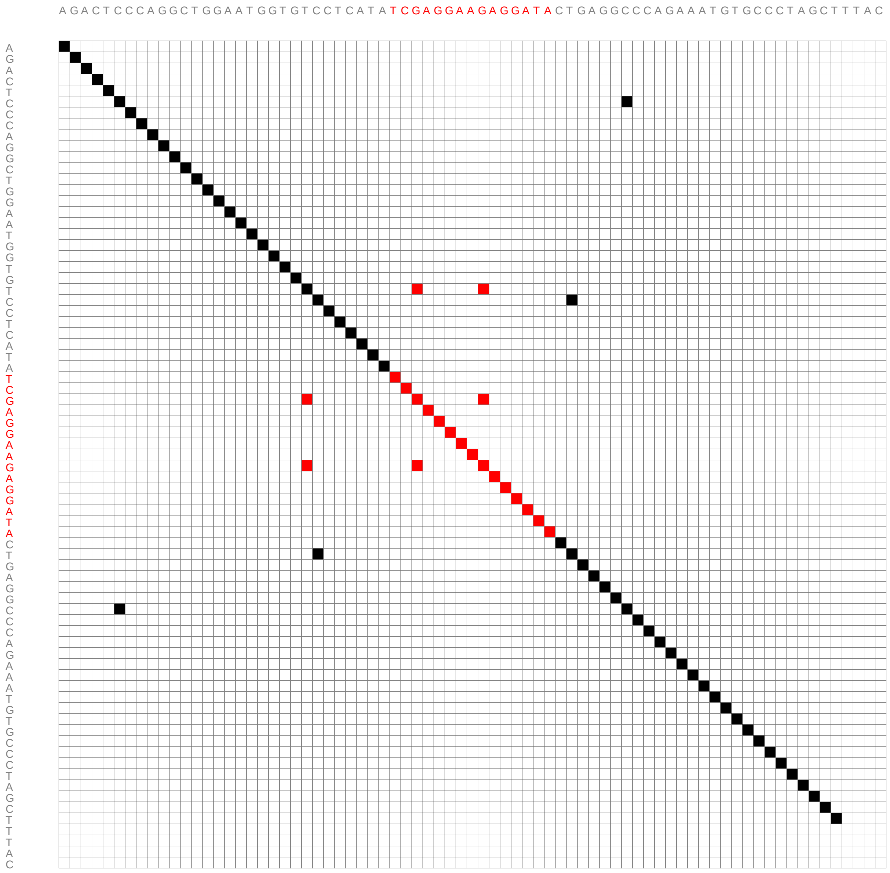
AGACTCCCAGGCTGGAATGGTGTCCTCATATCGAGGAAGAGGATACTGAGGCCCAGAAATGTGCCCTAGCTTTAC

HP11

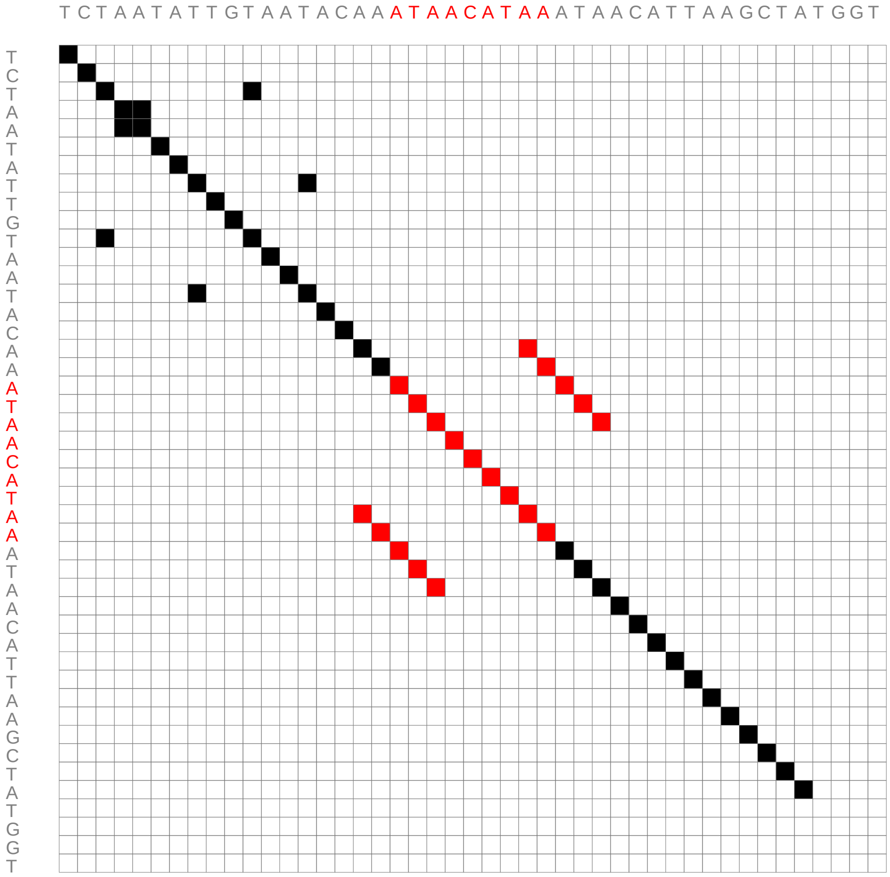

TCTAATATTGTAATACAAATAACATAAATAACATTAAGCTATGGT
TCTAATATTGTAATACAAATAACATAAATAACATTAAGCTATGGT

HP12

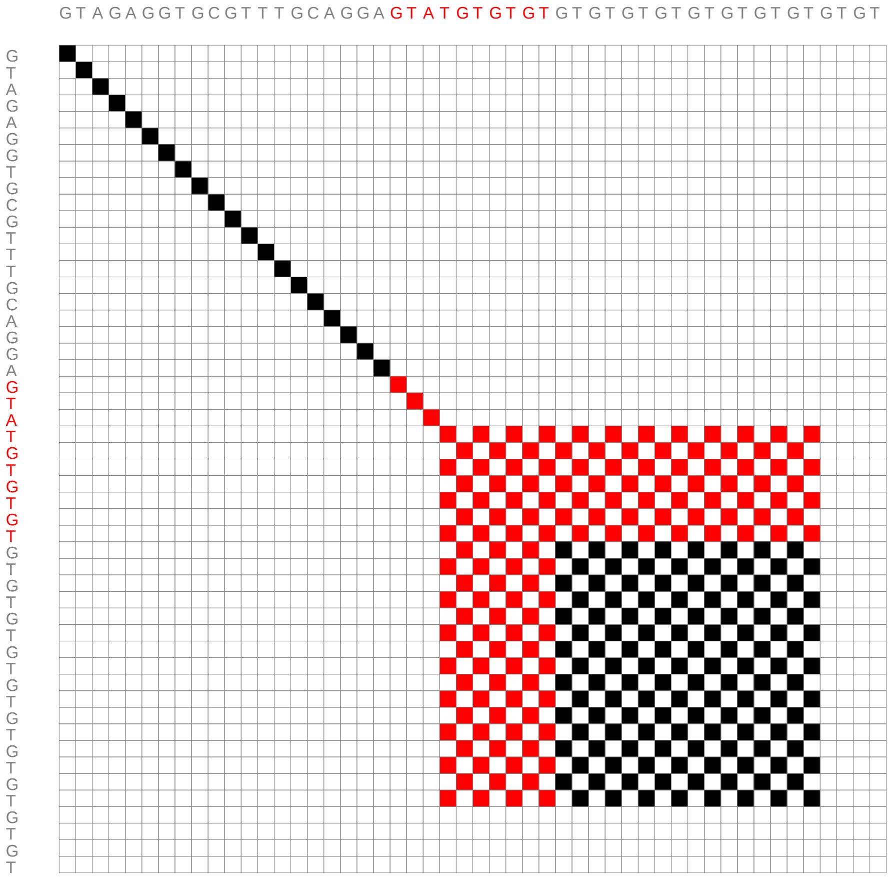

GTAGAGGTGCGTTTGCAGGAGTATGTGTGTGTGTGTGTGTGTGTGTGTGTGTGT
GTAGAGGTGCGTTTGCAGGAGTATGTGTGTGTGTGTGTGTGTGTGTGTGT

HP13

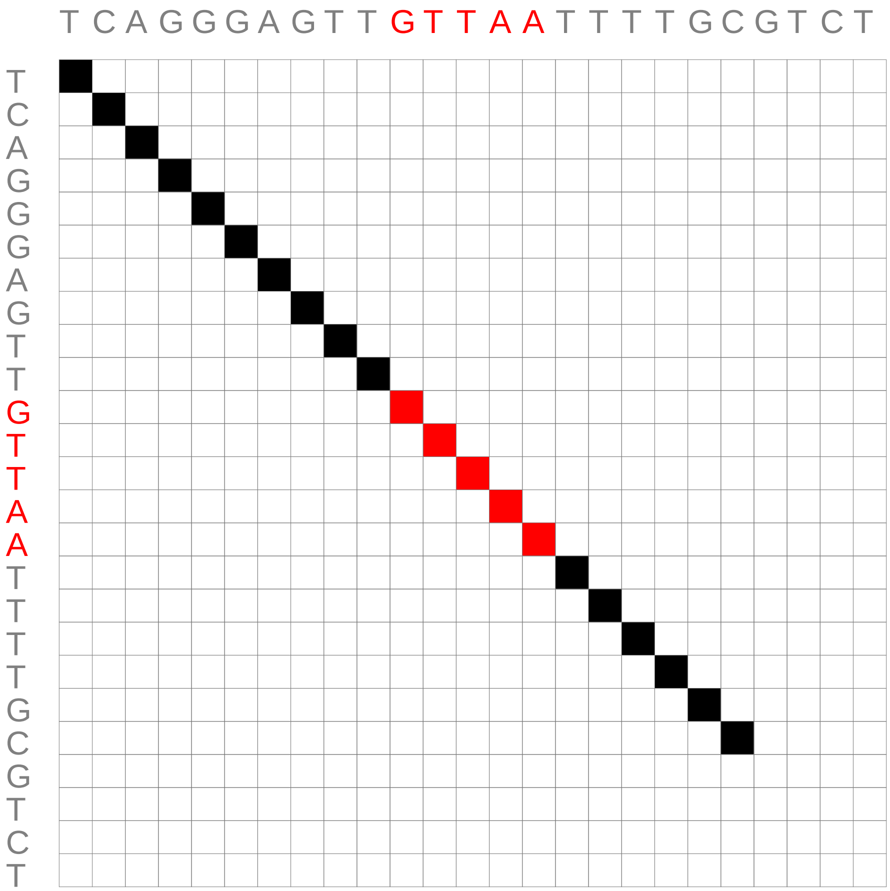

T C A G G G A G T T G T T A A T T T T G C G T C T
T C A G G G A G T T G T T A A T T T T G C G T C T

HP14

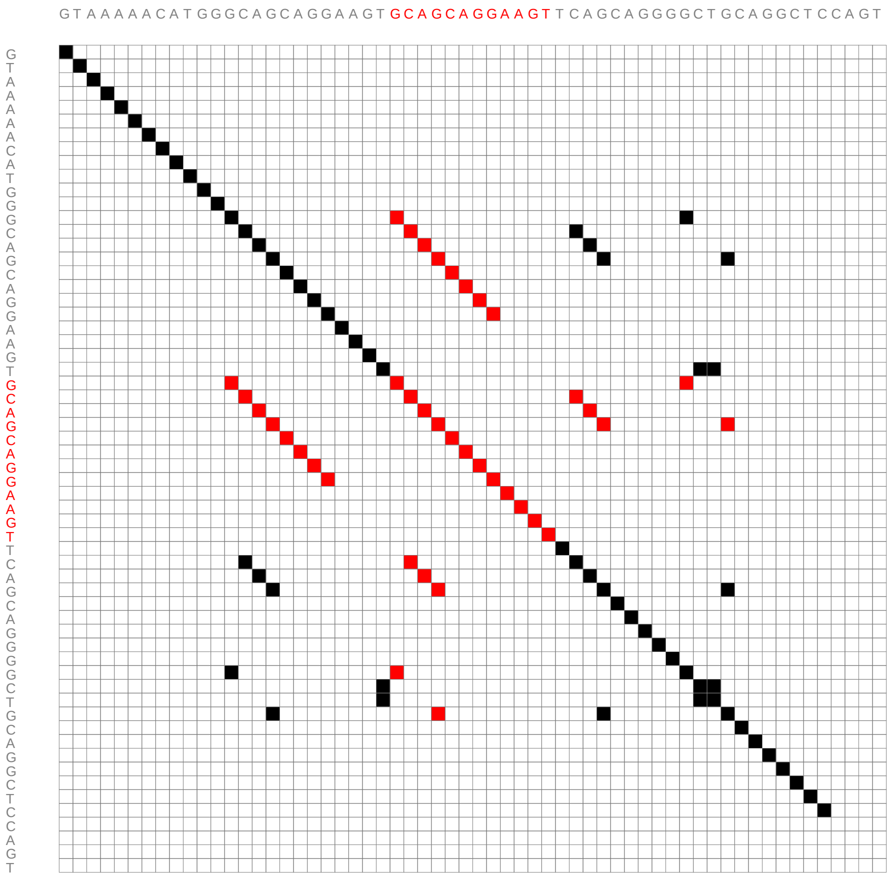

GTAAAAACATGGGCAGCAGGAAGTGCAGCAGGAAGTTCAGCAGGGGCTGCAGGCTCCAGT
GTAAAAACATGGGCAGCAGGAAGTGCAGCAGGAAGTTCAGCAGGGGCTGCAGGCTCCAGT

HP15

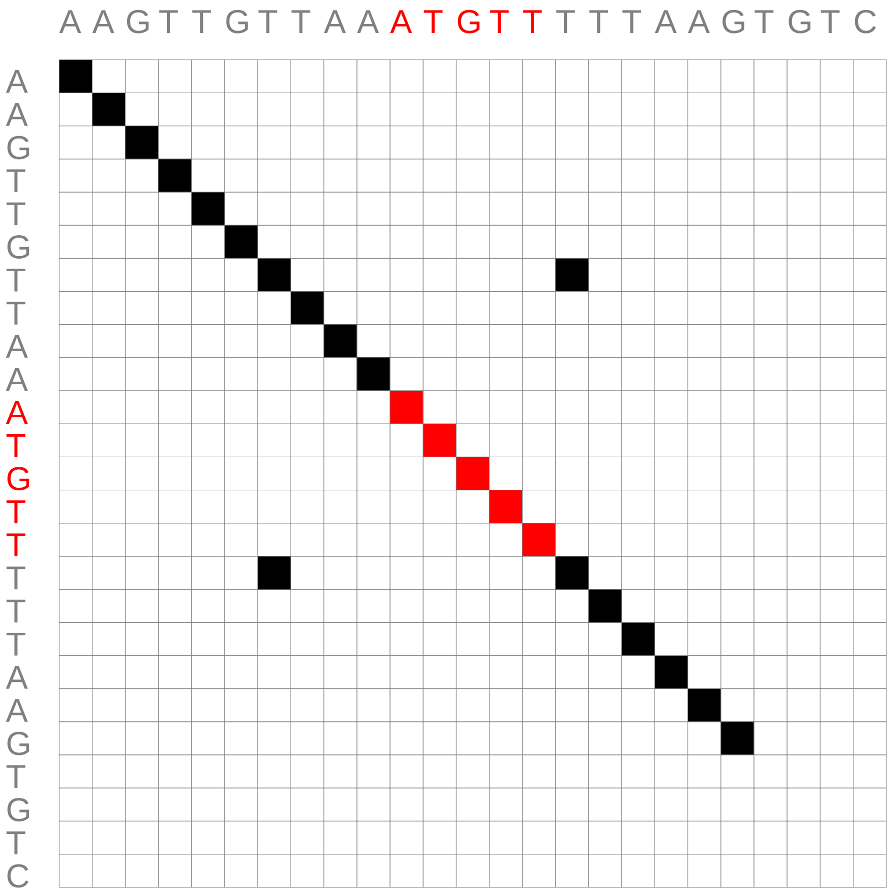
A A G T T G T T A A A T G T T T T T A A G T G T C
A A G T T G T T A A A T G T T T T T A A G T G T C

HP16

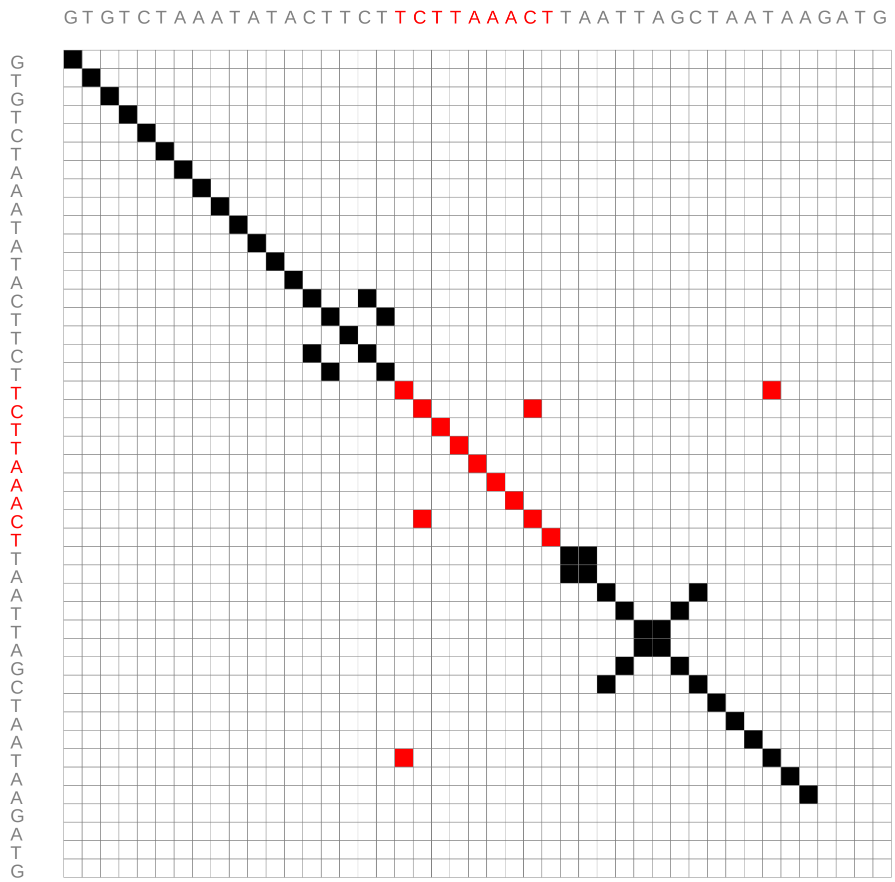

HP17

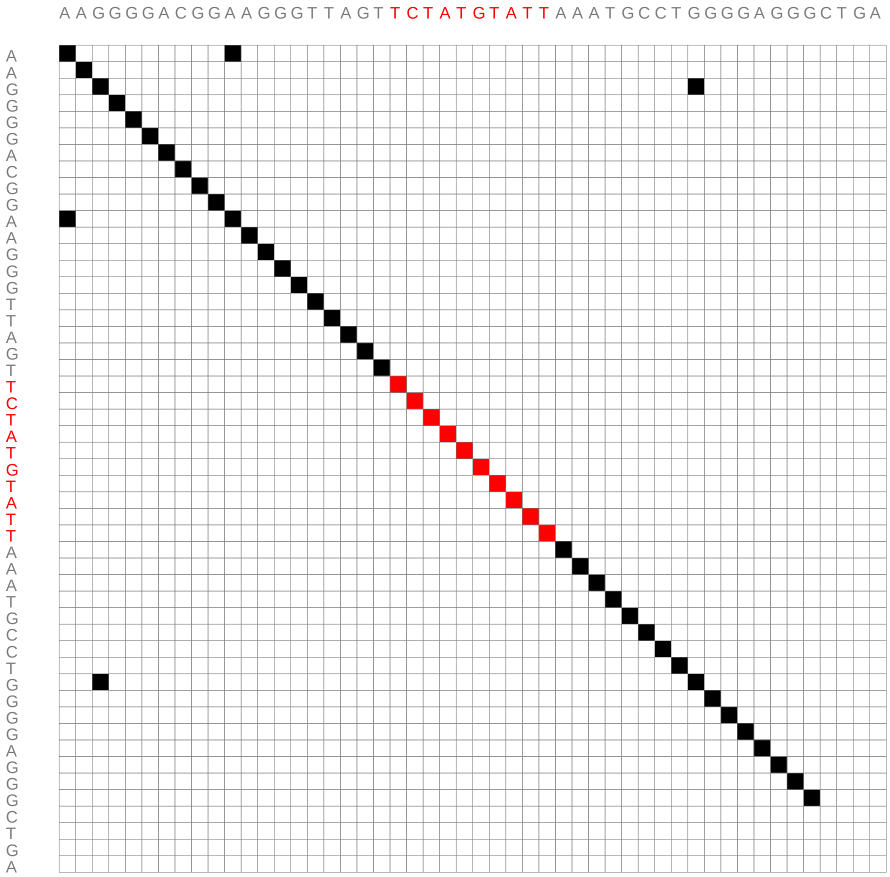
A A G G G G A C G G A A G G G T T A G T T T C T A T G T A T T A A A T G C C T G G G G A G G G C T G A
A A G G G G A C G G A A G G G T T A G T T C T A T G T A T T A A A T G C C T G G G G A G G G C T G A

HP18

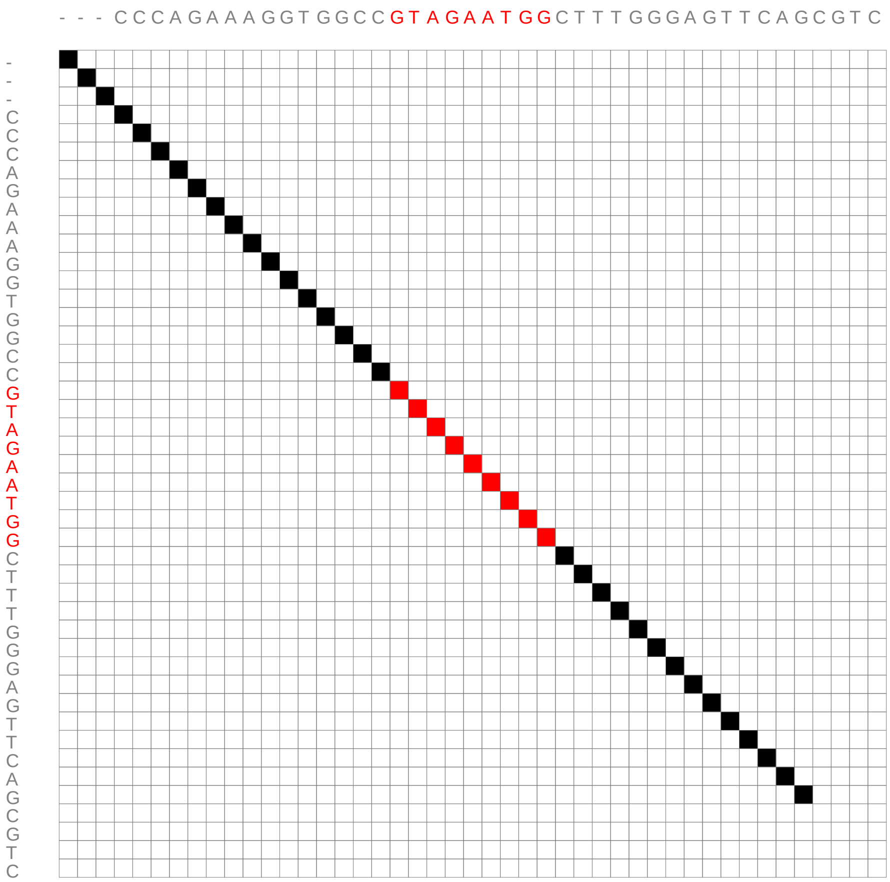
- - - CCCAGAAAGGTGGCCGTAGAATGGCTTTGGGAGTTCAGCGTC
- - - CCCAGAAAGGTGGCCGTAGAATGGCTTTGGGAGTTCAGCGTC

HP19

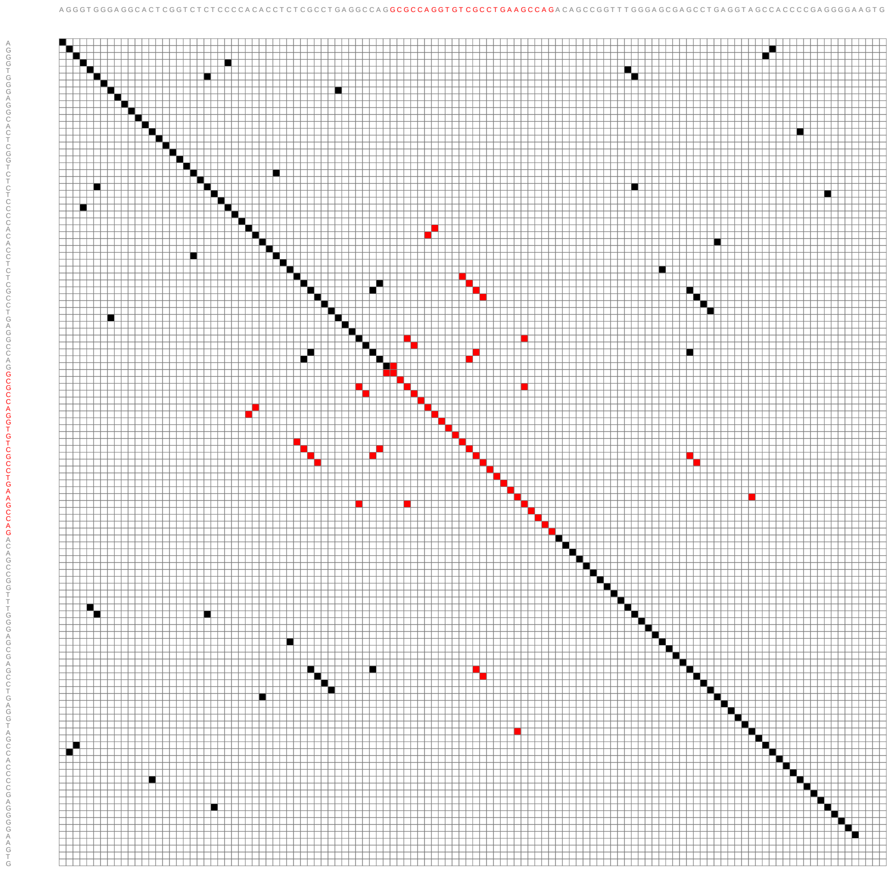

HP20

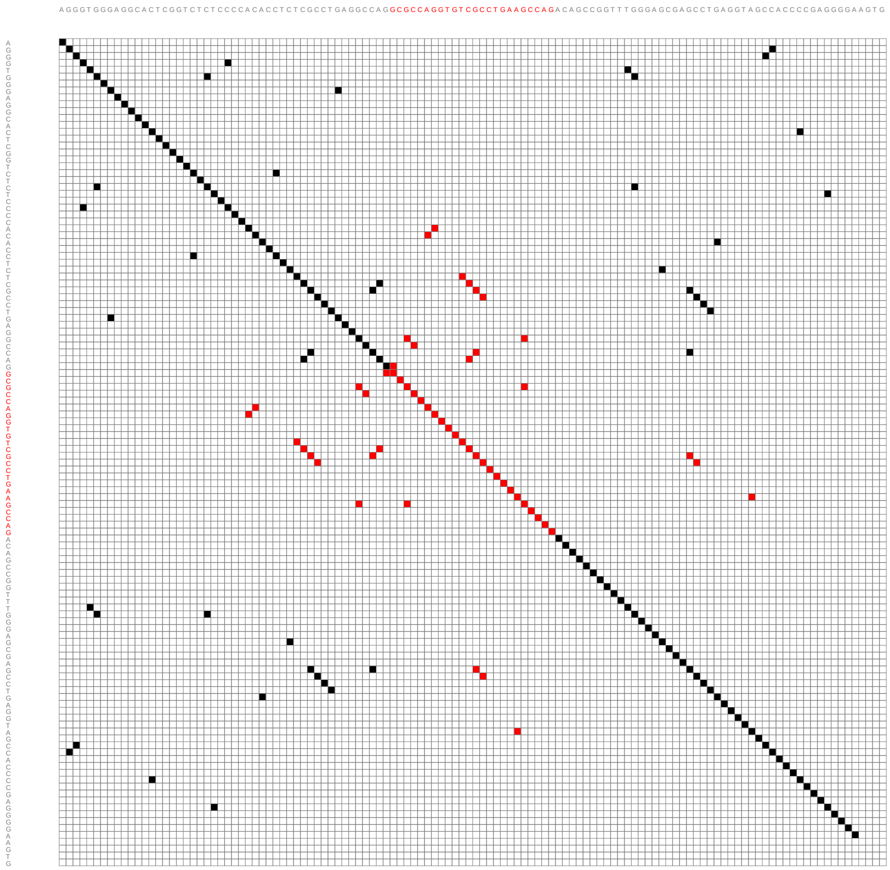
AGGGTGGGAGGCACTCGGTCTCTCCCCACACCTCTCGCCTGAGGCCAGGCGCCAGGTGTCGCCTGAAGCCAGACAGCCGGTTTGGGAGCGAGCCTGAGGTAGCCACCCCGAGGGGAAGTG

HP21

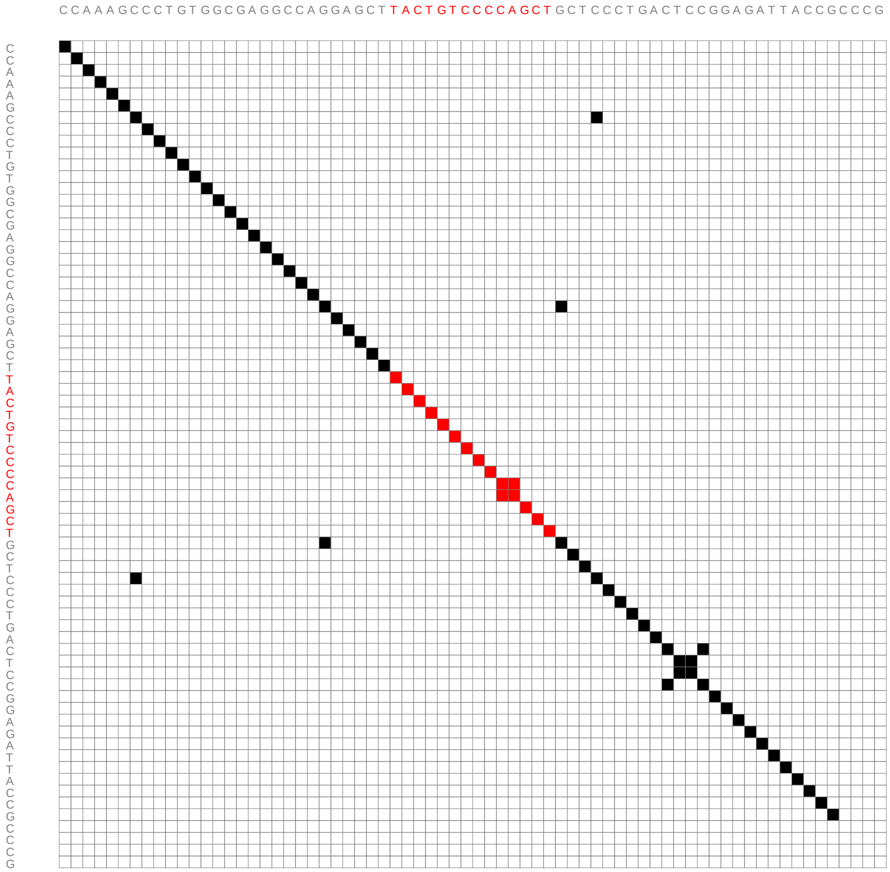
CCAAAGCCCTGTGGCGAGGCCAGGAGCTTACTGTCCCCAGCTGCTCCCTGACTCCGGAGATTACCGCCCG

HP22

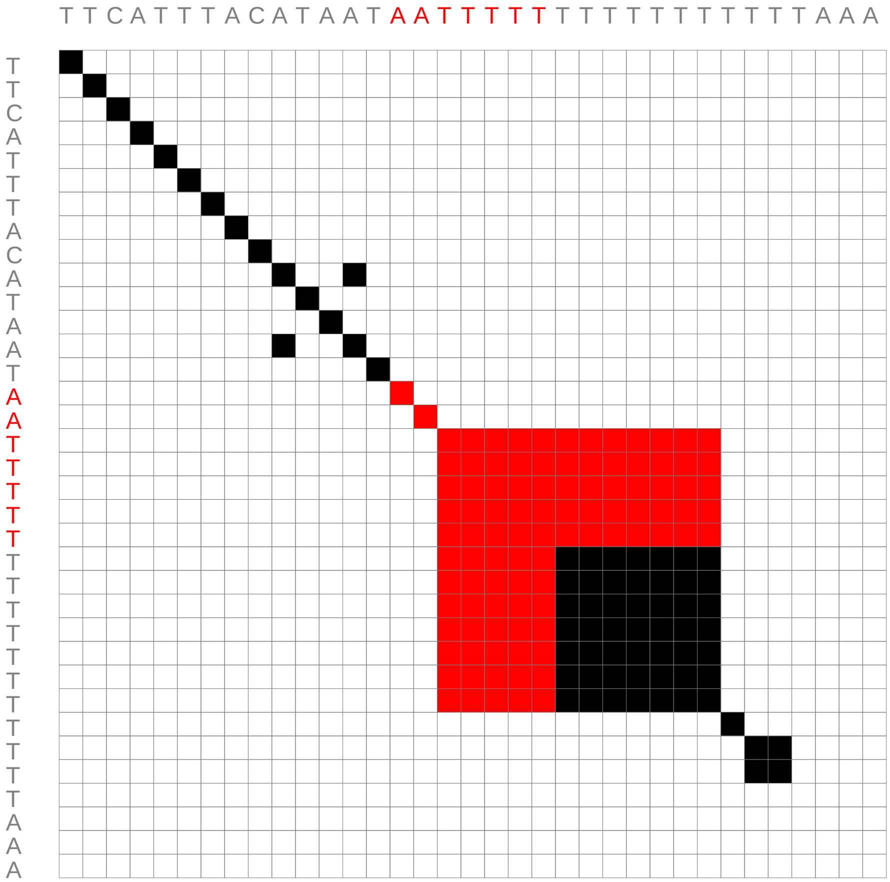
T T C A T T T A C A T A A T A A T T T T T T T T T T T T T T T T A A A
T T C A T T T A C A T A A T A A T T T T T T T T T T T T T T T T A A A

HP23

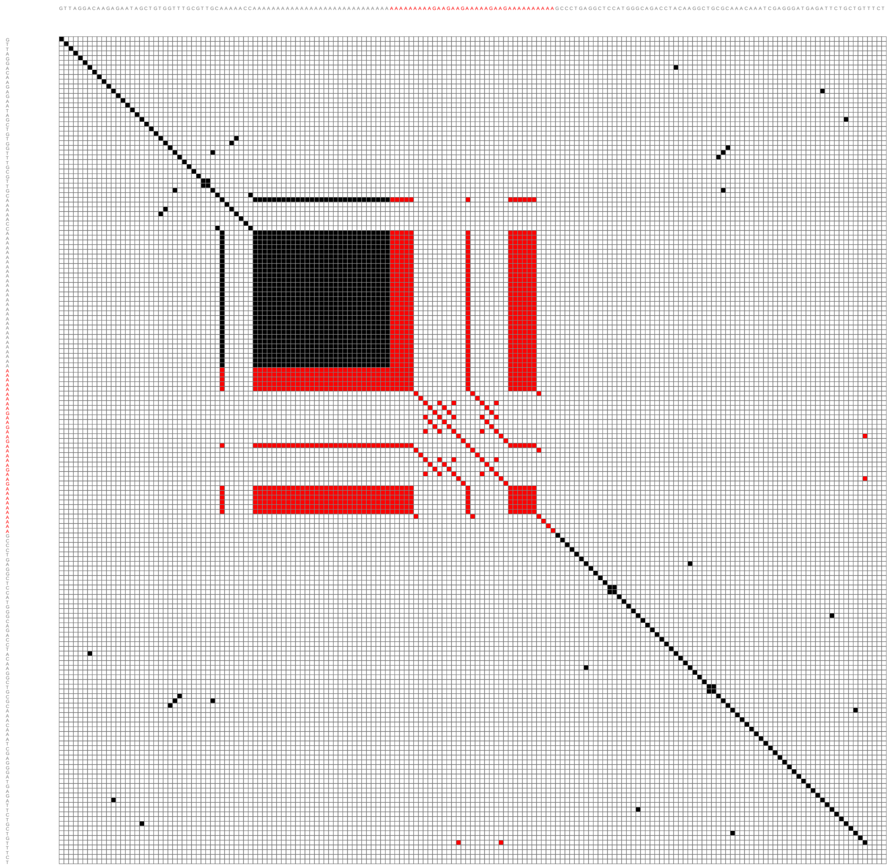
GTTAGGACAAGAGAATAGCTGTGGTTTGCGTTGCAAAAACCAAAAAAAAAAAAAAAAAAAAAAAAAAAAAAAAAAAAAAAGAAGAAGAAAAAGAAGAAAAAAAAAAGCCCTGAGGCTCCATGGGCAGACCTACAAGGCTGCGCAAACAAATCGAGGGATGAGATTCTGCTGTTTCT

HP24

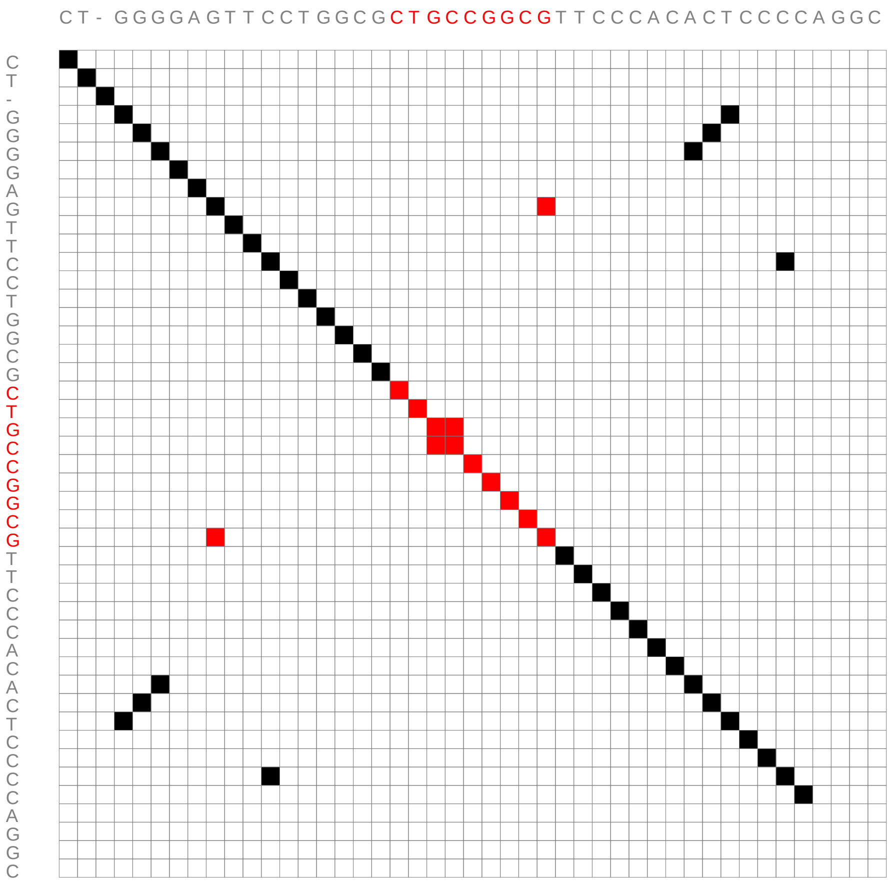

HP25

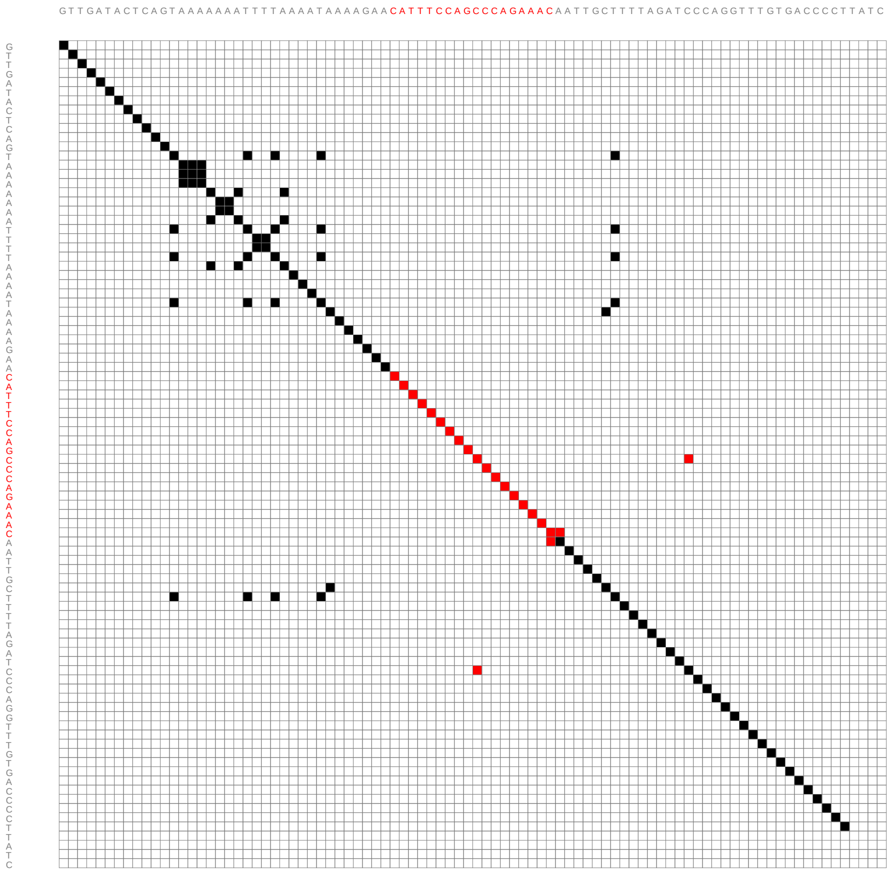
GTTGATACTCAGTAAAAAAAATTTTAAAATAAAAGAACATTTCCAGCCCAGAAACAATTGCTTTTAGATCCCAGGTTTGTGACCCCTTATC

HP26

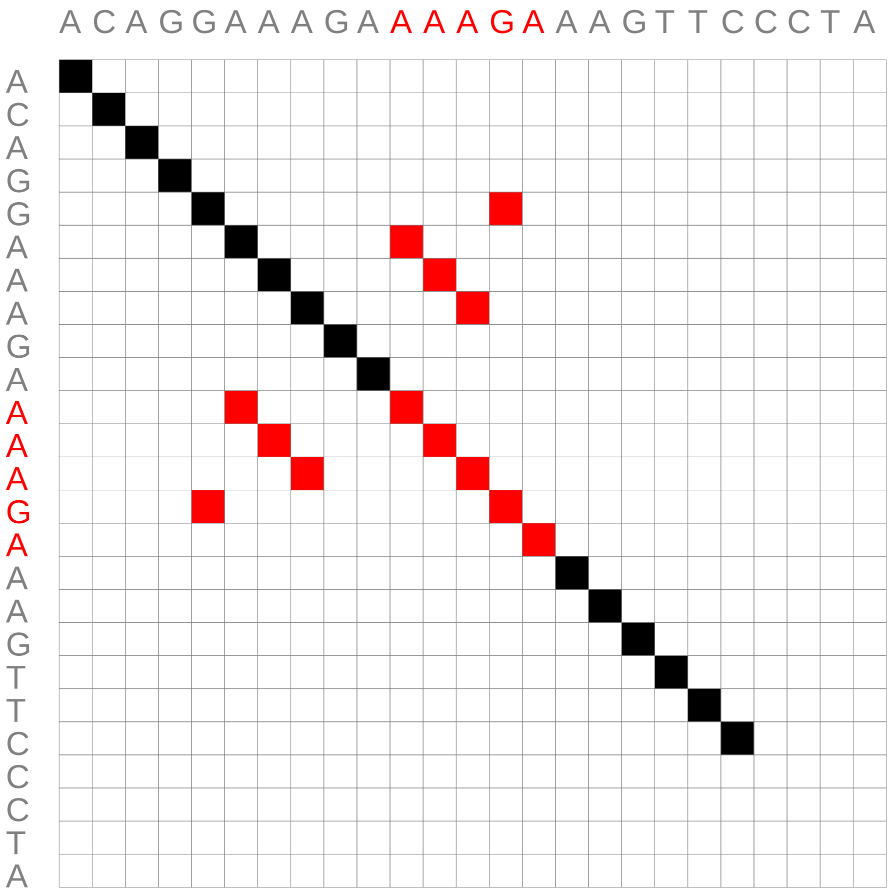
A C A G G A A A G A A A A G A A A G T T C C C T A
A C A G G A A A G A A A A G A A A G T T C C C T A

HP27

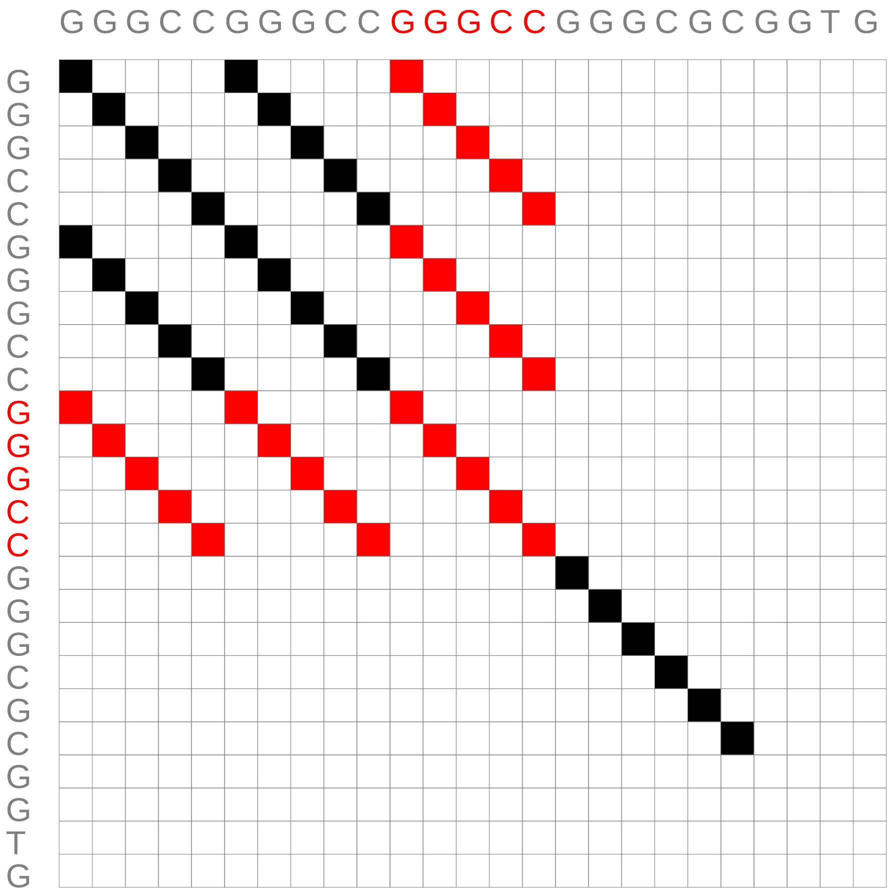
G G G C C G G G C C G G G C C G G G C G C G G T G
G G G C C G G G C C G G G C C G G G C G C G G T G

HP28

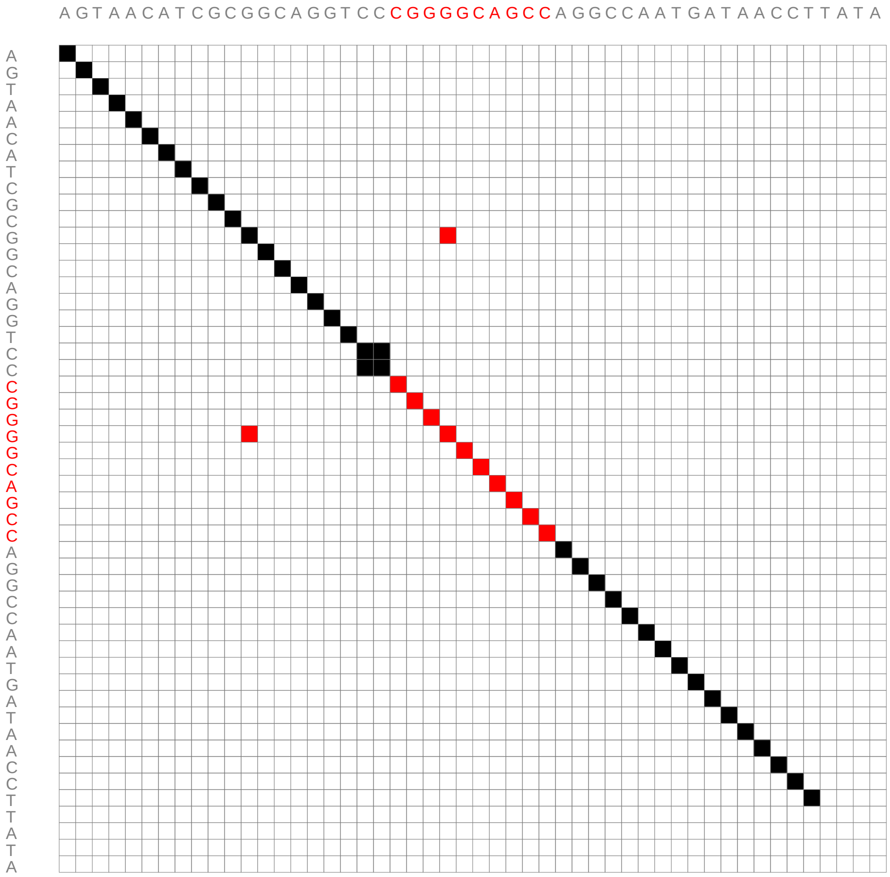
AGTAACATCGCGGCAGGTCCCGGGGCAGCCAGGCCAATGATAACCTTATA
AGTAACATCGCGGCAGGTCCCGGGGCAGCCAGGCCAATGATAACCTTATA

HP29

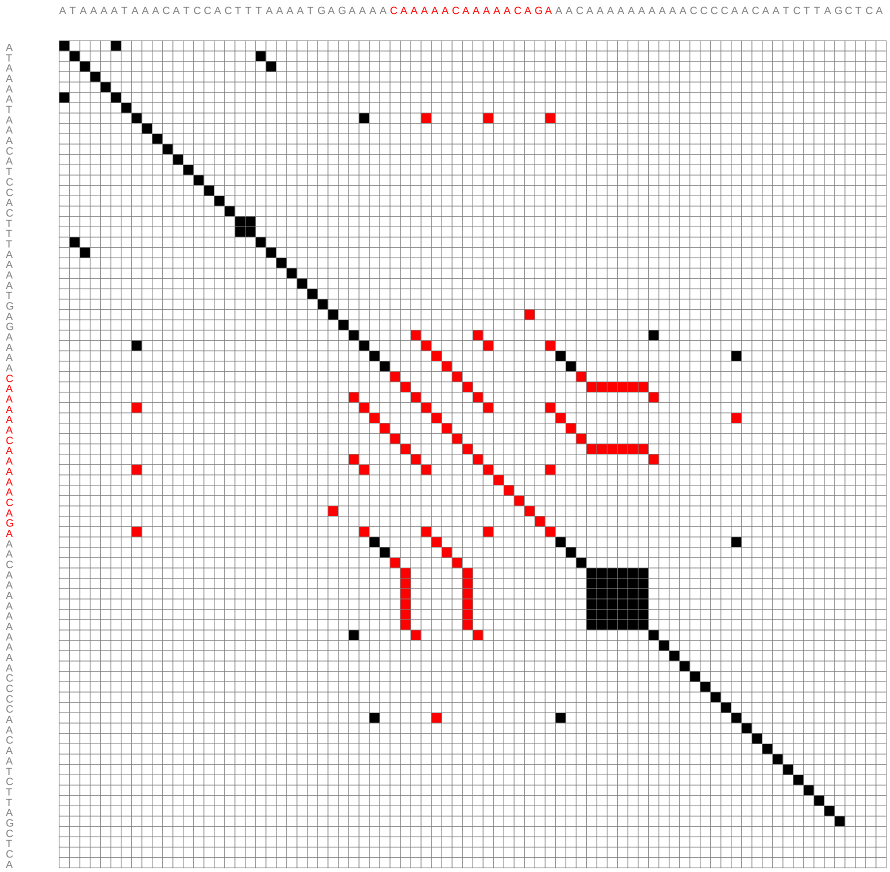
A T A A A A T A A A C A T C C A C T T T A A A A T G A G A A A A C A A A A A C A A A A A C A G A A A C A A A A A A A A A A A C C C C A A C A A T C T T A G C T C A

HP30

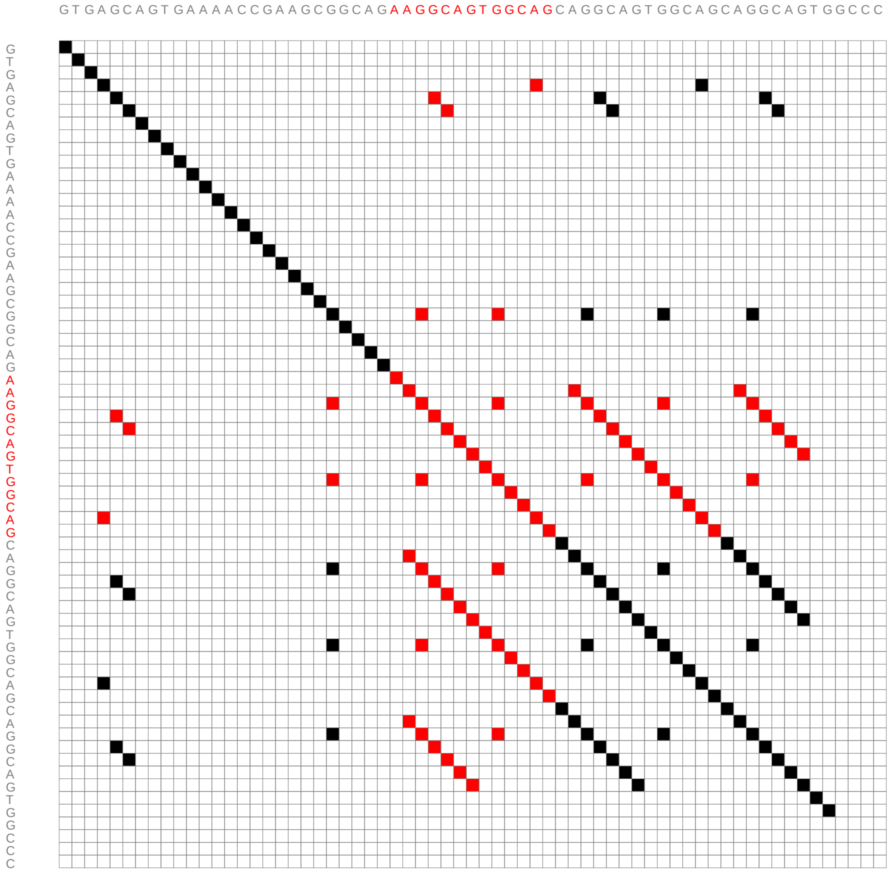
GTGAGCAGTGAAAACCGAAGCGGCAGAAGGCAGTGGCAGCAGGCAGTGGCAGCAGGCAGTGGCCC

HP31

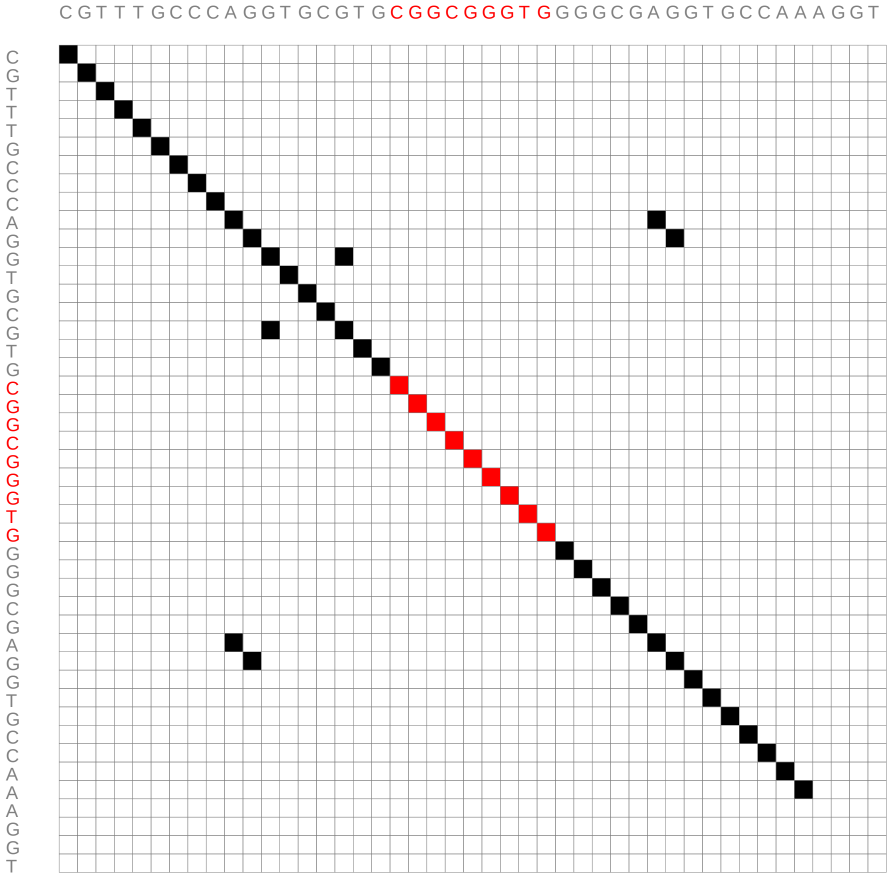

HP32

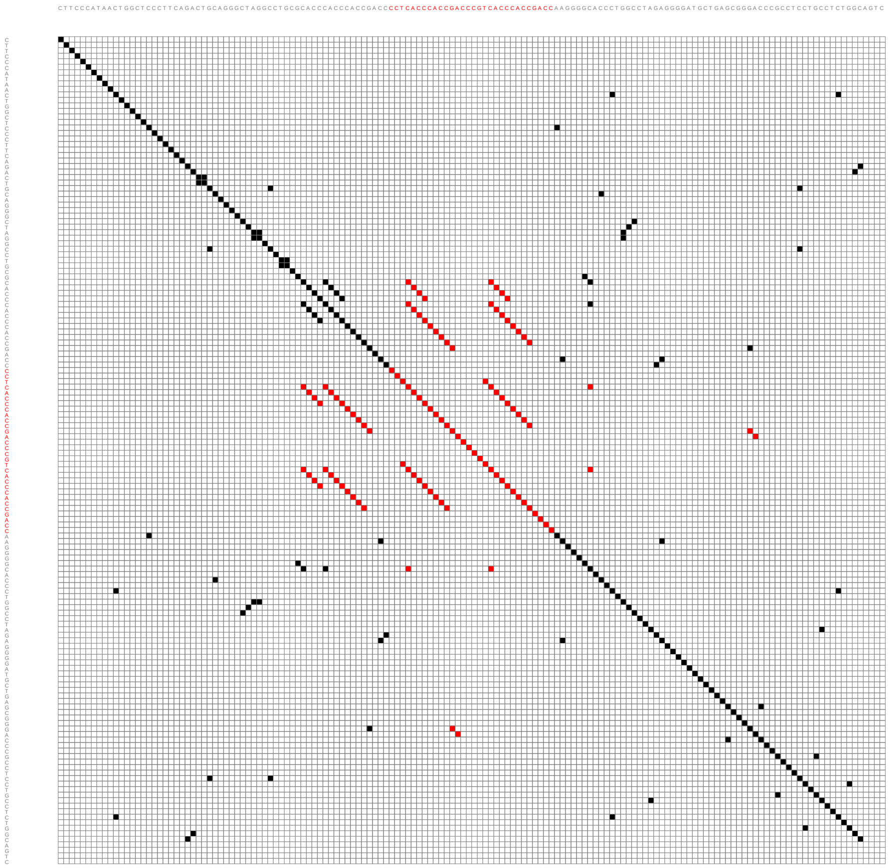

CTTCCCATAACTGGCTCCCTTCAGACTGCAGGGCTAGGCCTGCGCACCCACCCACCGACCCCTCACCCACCGACCCGTCACCCACCGACCAAGGGGCACCCTGGCCTAGAGGGGATGCTGAGCGGGACCCGCCTCCTGCCTCTGGCAGTC

HP33

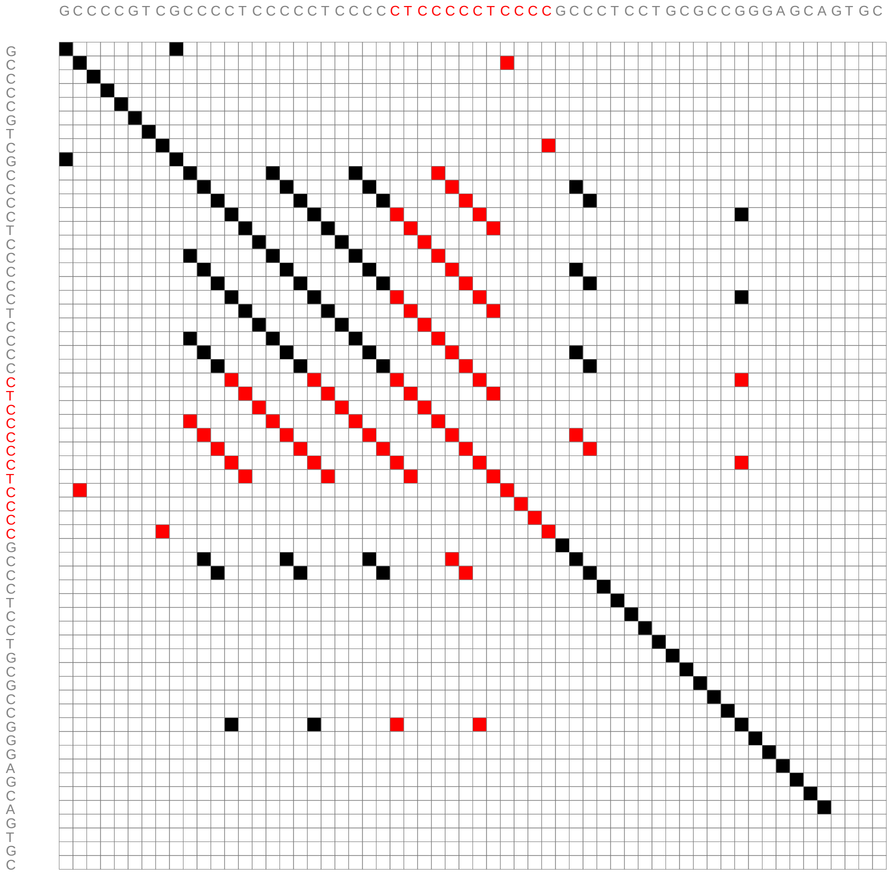
GCCCCGTCGCCCCTCCCCCTCCCCCTCCCCCTCCCCGCCCTCCTGCGCCGGGAGCAGTGC

HP34

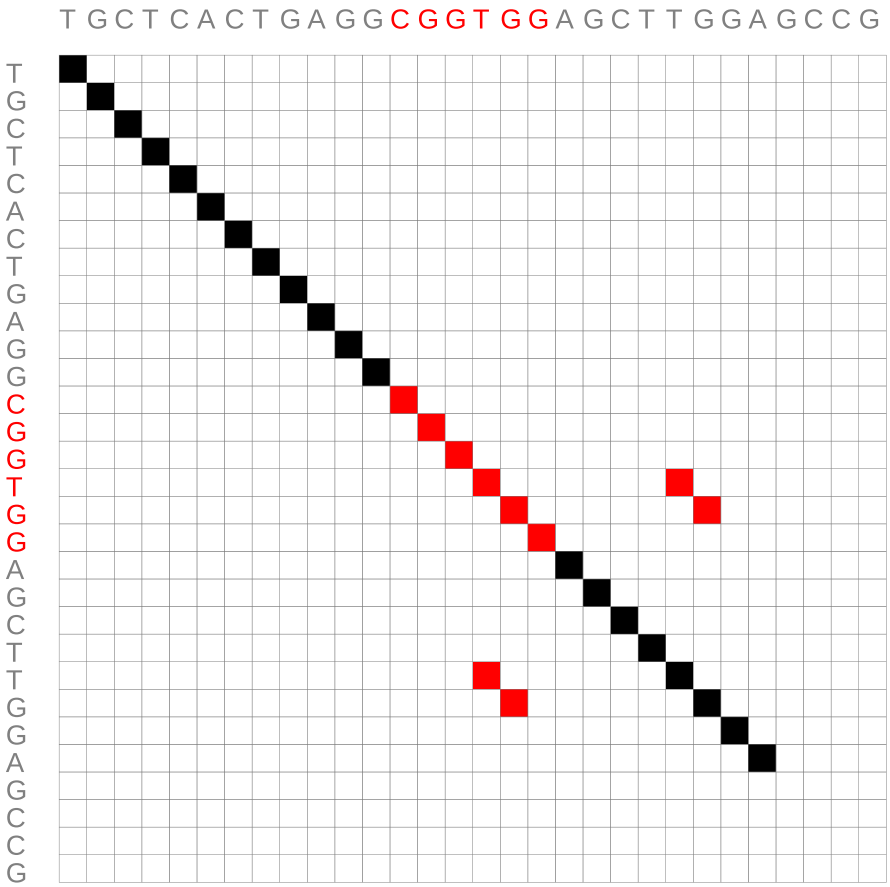
T G C T C A C T G A G G C G G T G G A G C T T G G A G C C G
T G C T C A C T G A G G C G G T G G A G C T T G G A G C C G

HP35

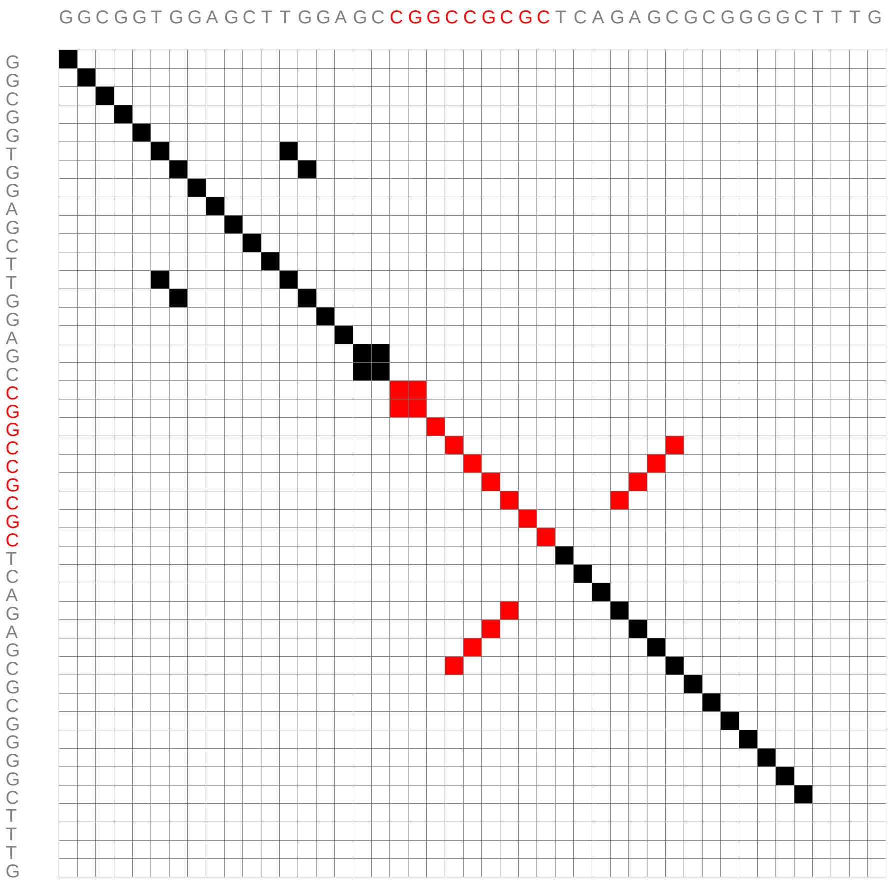

HP36

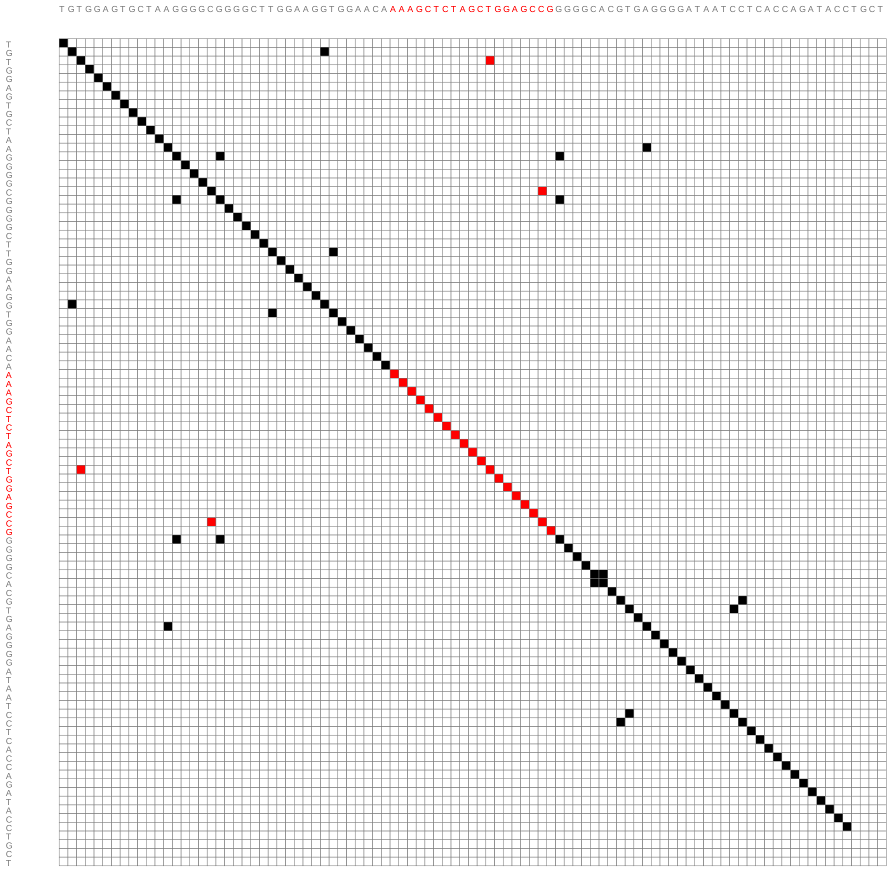
TGTGGAGTGCTAAGGGGCGGGGCTTGGAAGGTGGAACAAAAGCTCTAGCTGGAGCCGGGGGCACGTGAGGGGATAATCCTCACCAGATACCTGCT

HP37

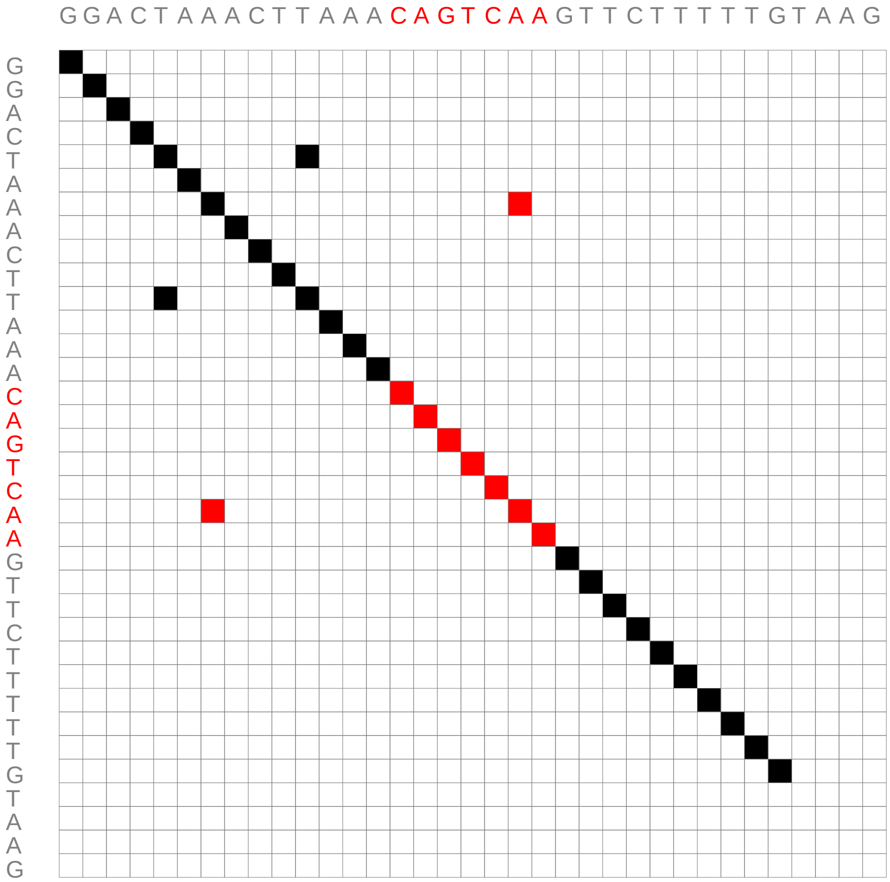
GGACTAAACTTAAACAGTCAAGTTCTTTTTGTAAG
GGACTAAACTTAAACAGTCAAGTTCTTTTTGTAAG

HP38

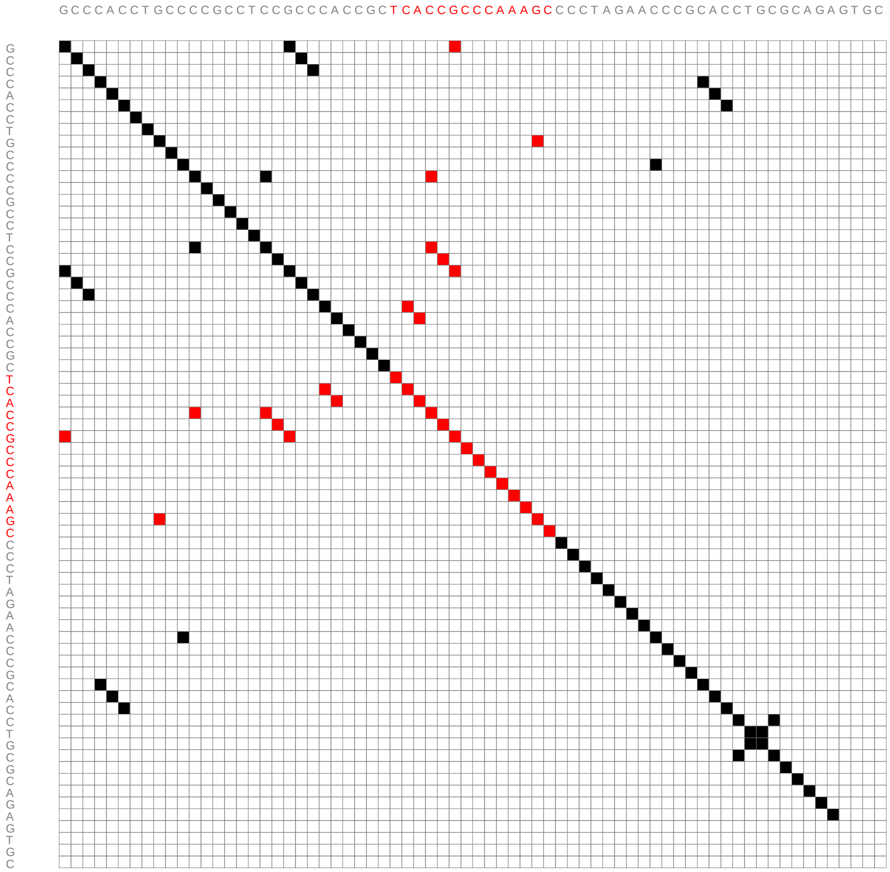

HP39

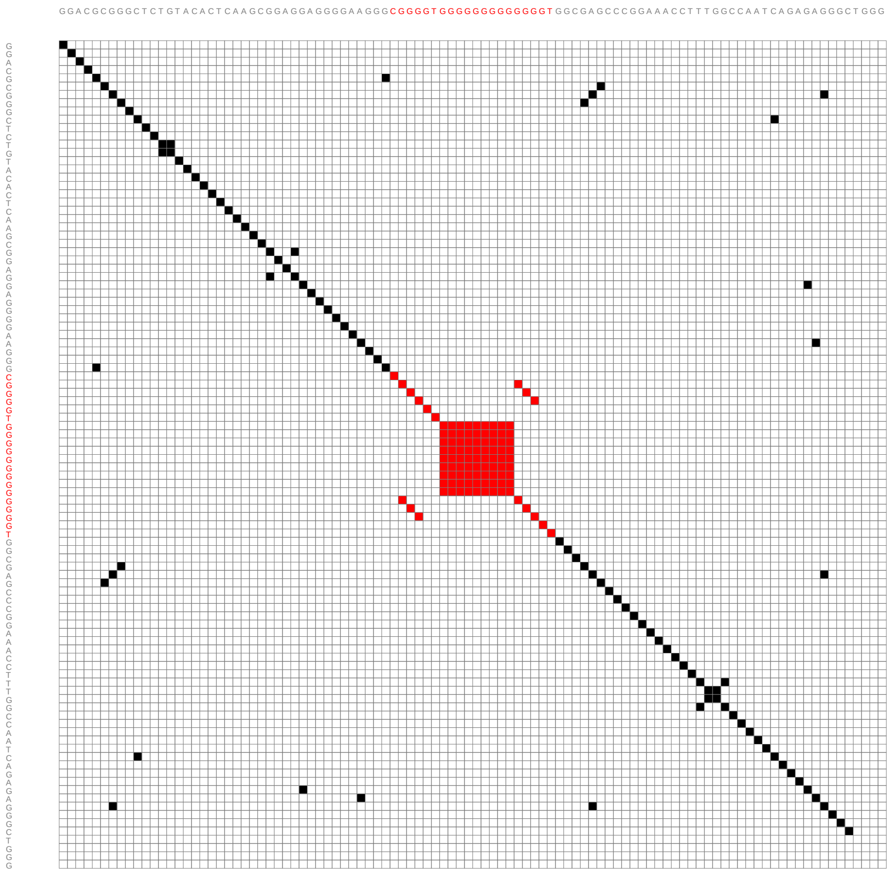
GGACGCGGGCTCTGTACACTCAAGCGGAGGAGGGGAAGGGCGGGGTGGGGGGGGGGGGGTGGCGAGCCCGGAAACCTTTGGCCAATCAGAGAGGGCTGGG

HP40

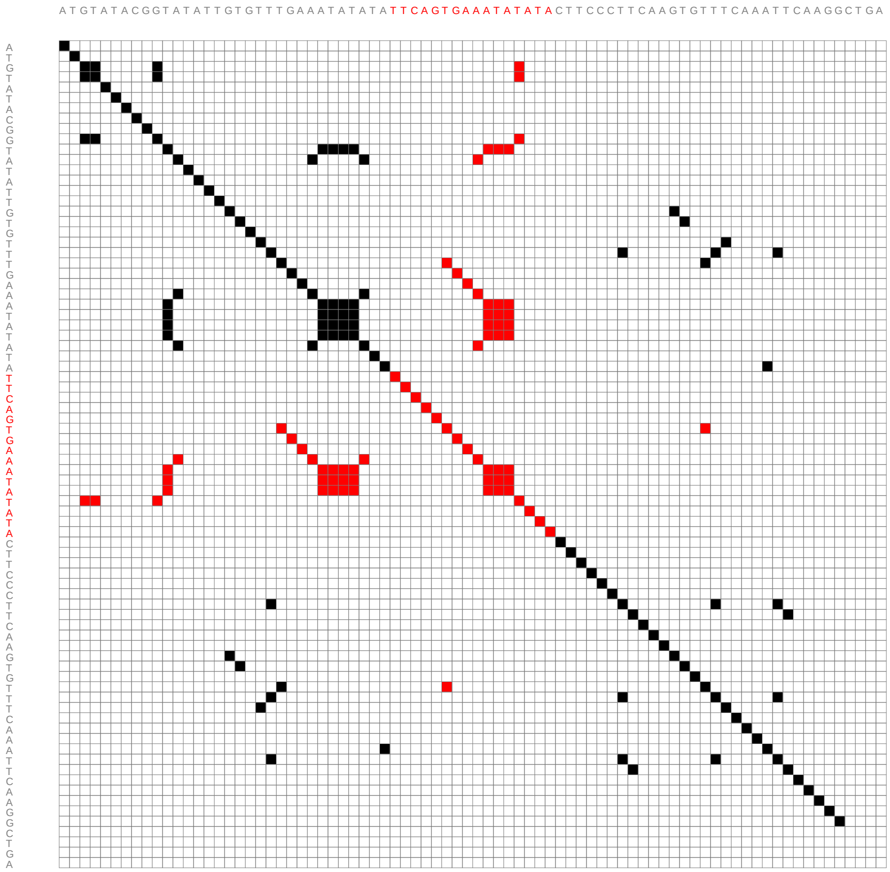

HP41

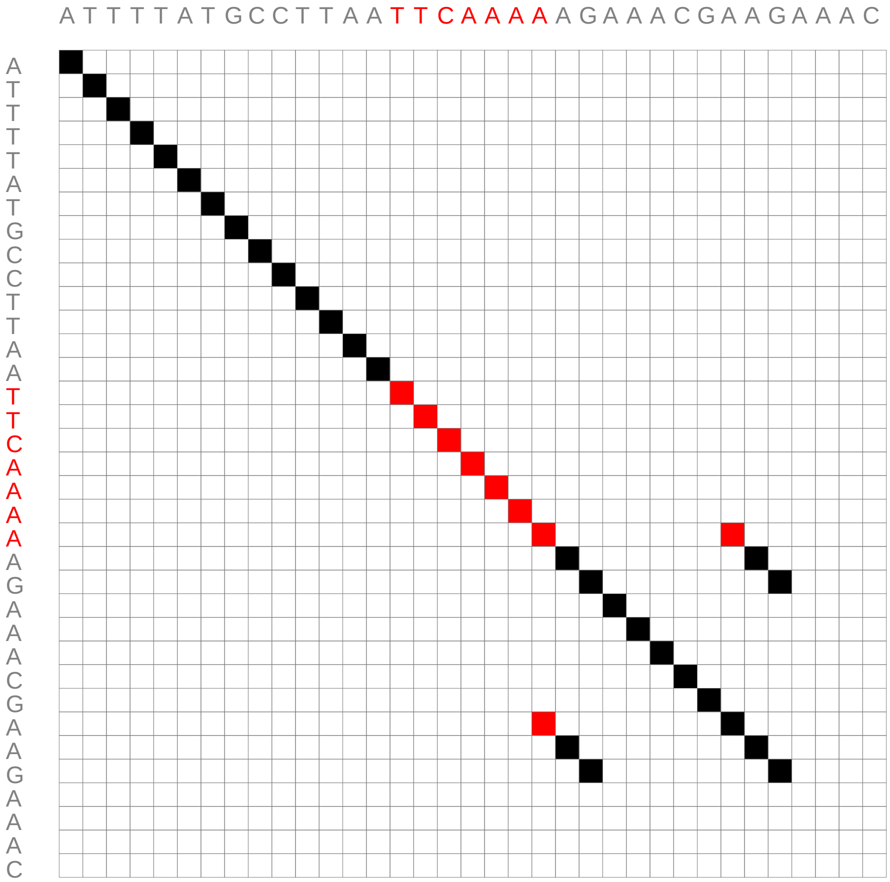
A T T T T A T G C C T T A A T T C A A A A A G A A A C G A A G A A A C
A T T T T A T G C C T T A A T T C A A A A A G A A A C G A A G A A A C

HP42

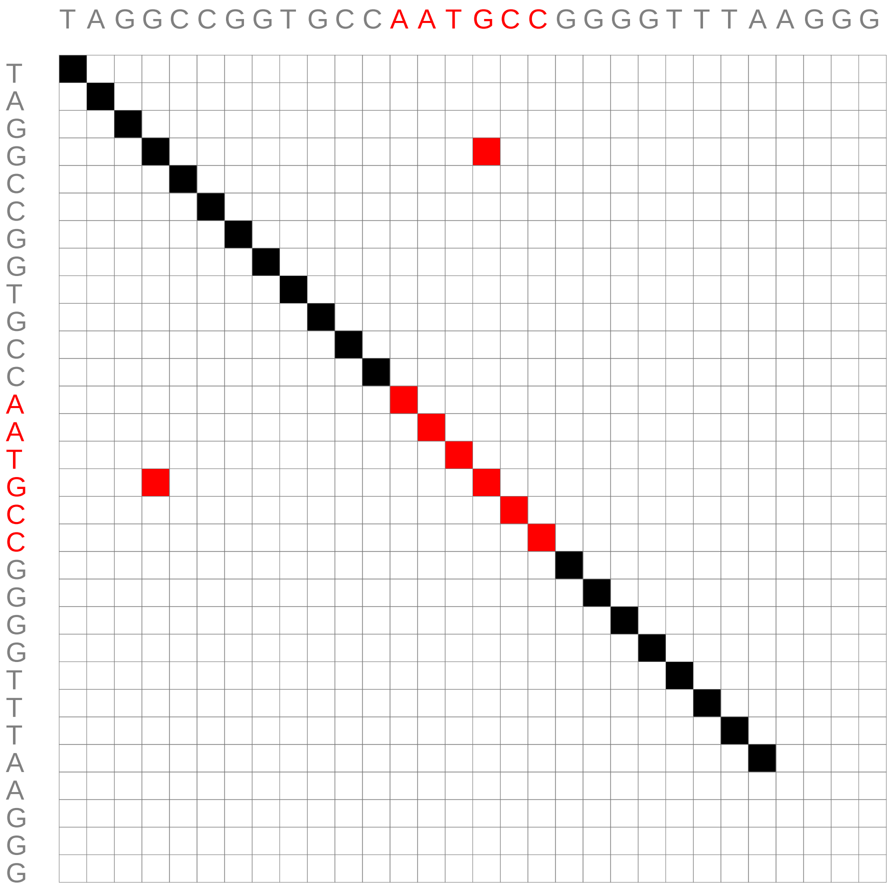

T A G G C C G G T G C C A A T G C C G G G G T T T A A G G G
T A G G C C G G T G C C A A T G C C G G G G T T T A A G G G

HP43

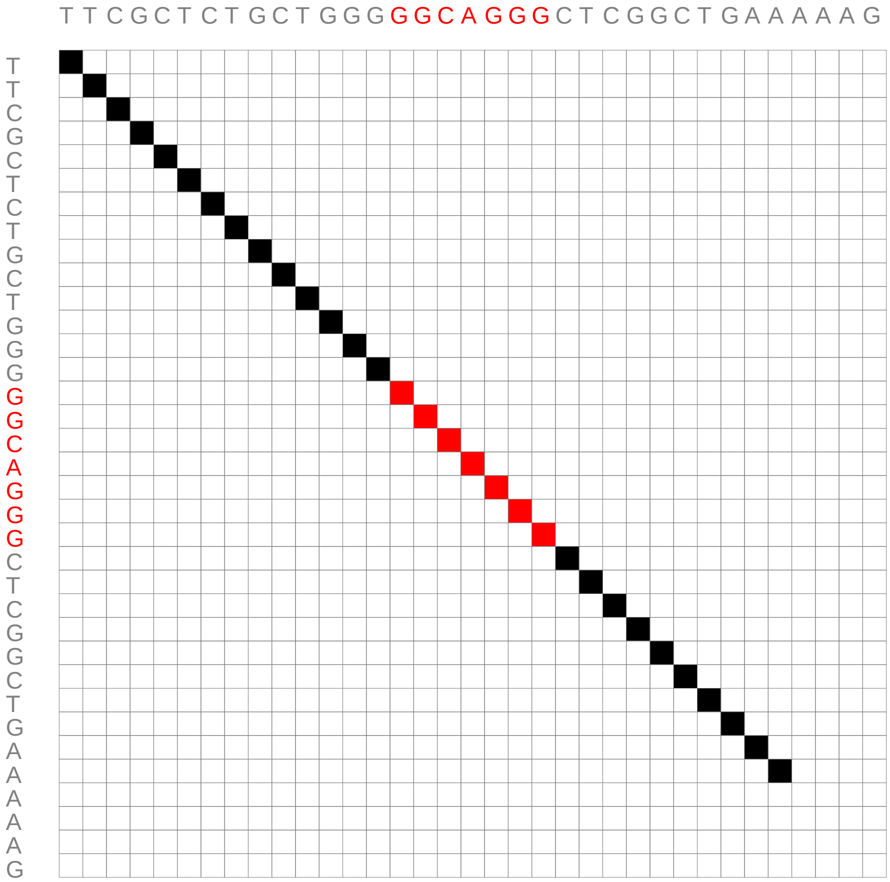
T T C G C T C T G C T G G G G G C A G G G C T C G G C T G A A A A A G
T T C G C T C T G C T G G G G G C A G G G C T C G G C T G A A A A A G

HP44

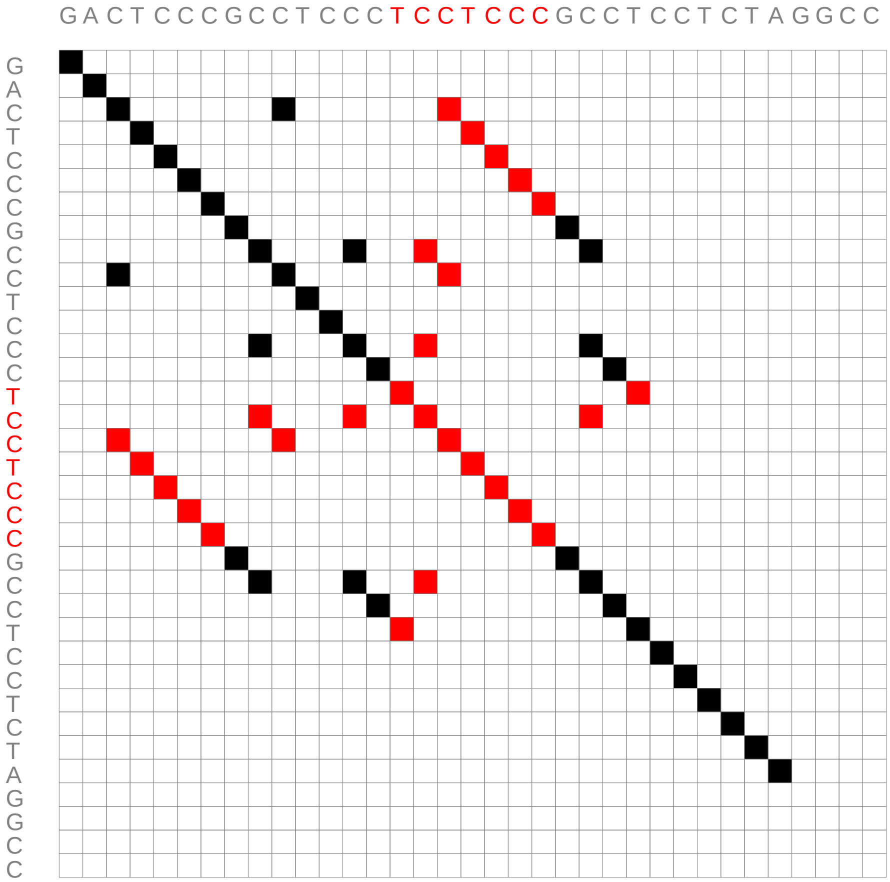
G A C T C C C G C C T C C C T C C T C C C G C C T C C T C T A G G C C
G A C T C C C G C C T C C C T C C T C C C G C C T C C T C T A G G C C

HP45

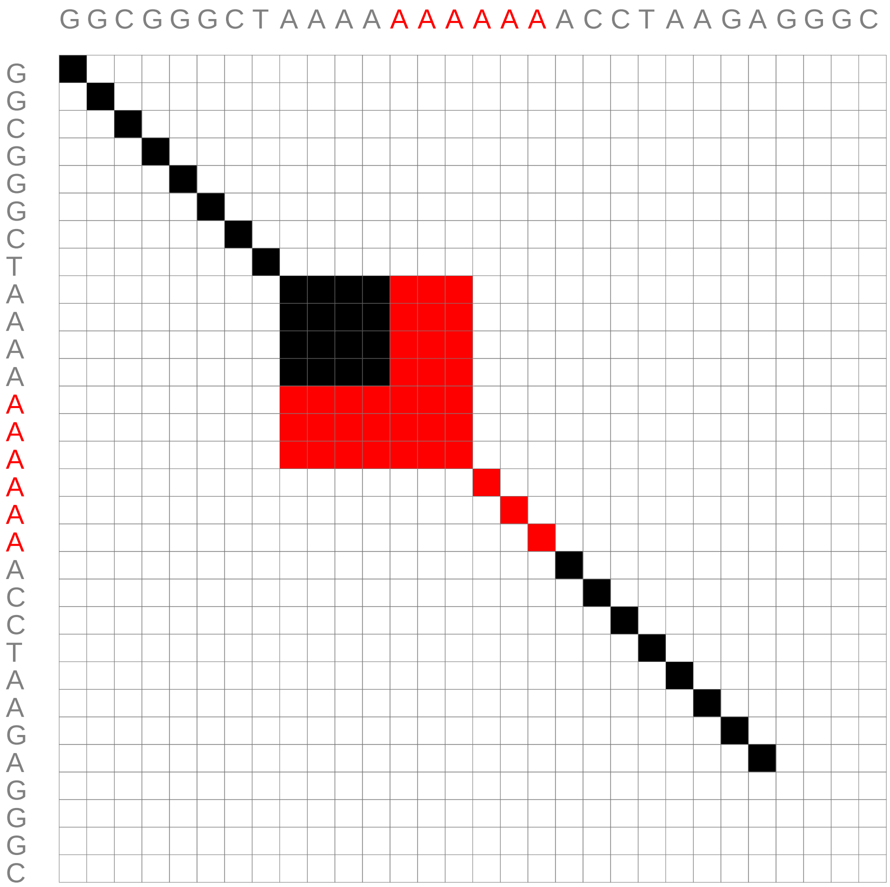
G G C G G G C T A A A A A A A A A A A C C T A A G A G G G C
G G C G G G C T A A A A A A A A A A A C C T A A G A G G G C

HP46

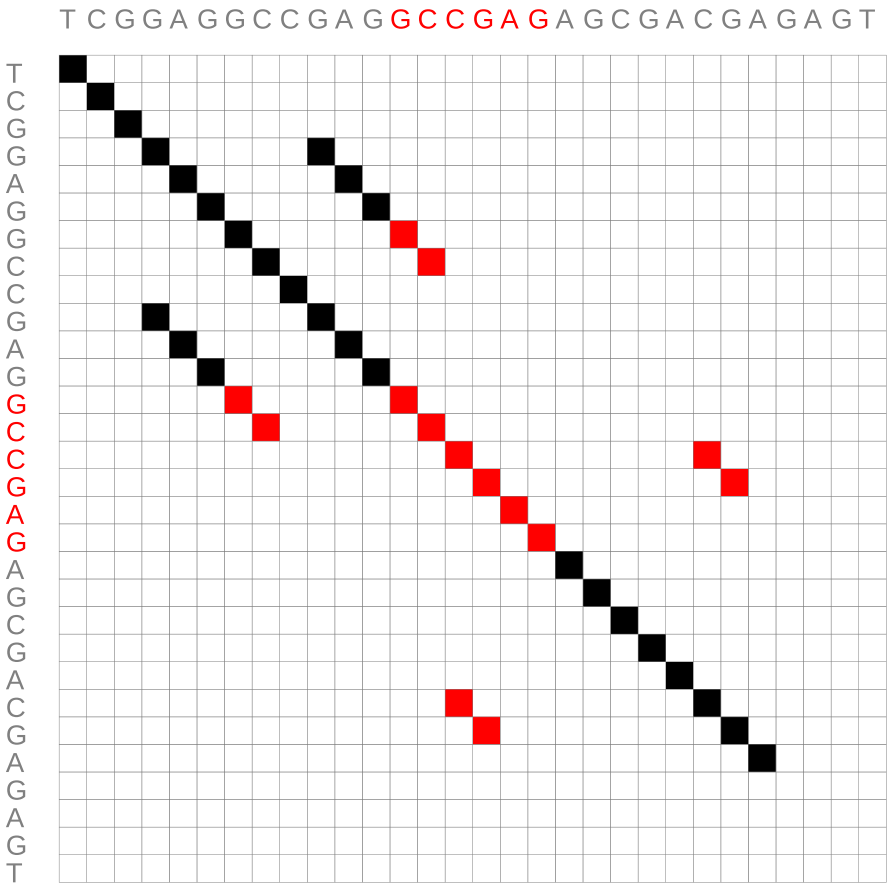
T C G G A G G C C G A G G C C G A G A G C G A C G A G A G T
T C G G A G G C C G A G G C C G A G A G C G A C G A G A G T

HP47

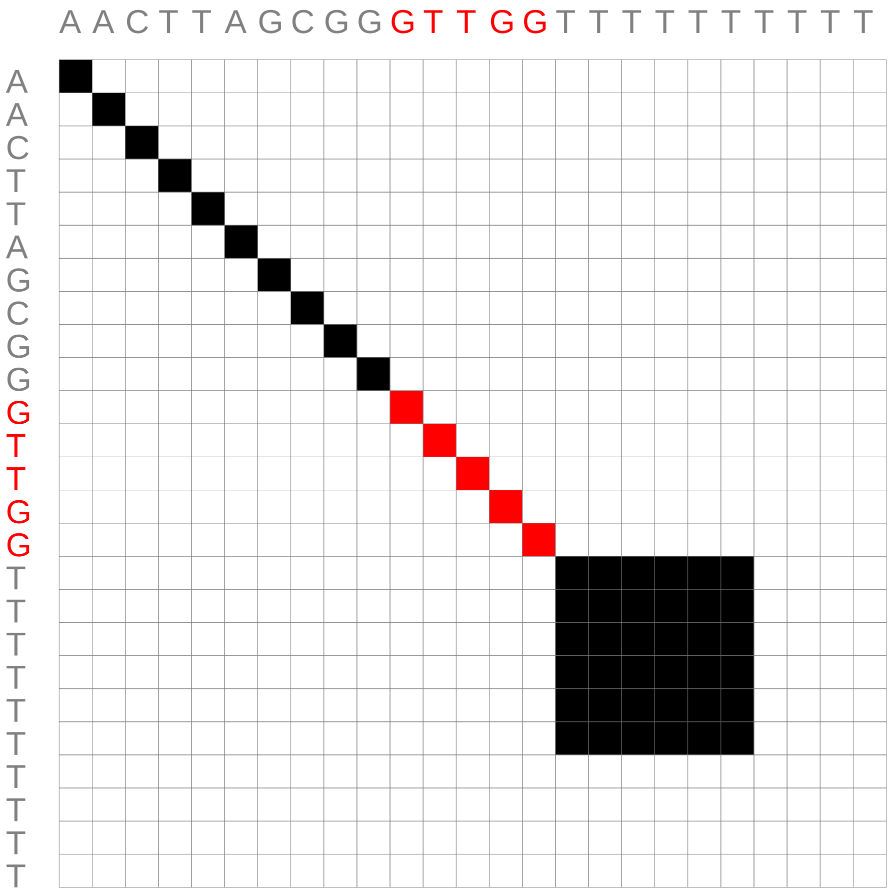
A A C T T A G C G G G T T G G T T T T T T T T T T
A A C T T A G C G G G T T G G T T T T T T T T T T

HP48

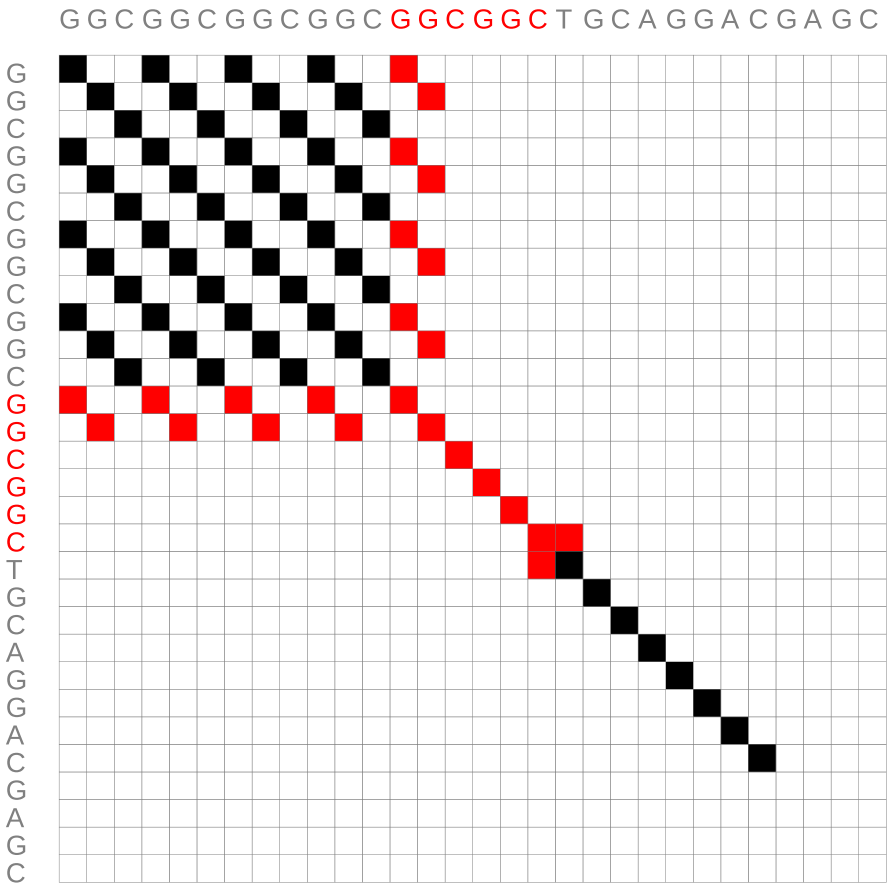
G G C G G C G G C G G C G G C G G C T G C A G G A C G A G C
G G C G G C G G C G G C G G C G G C T G C A G G A C G A G C

HP49

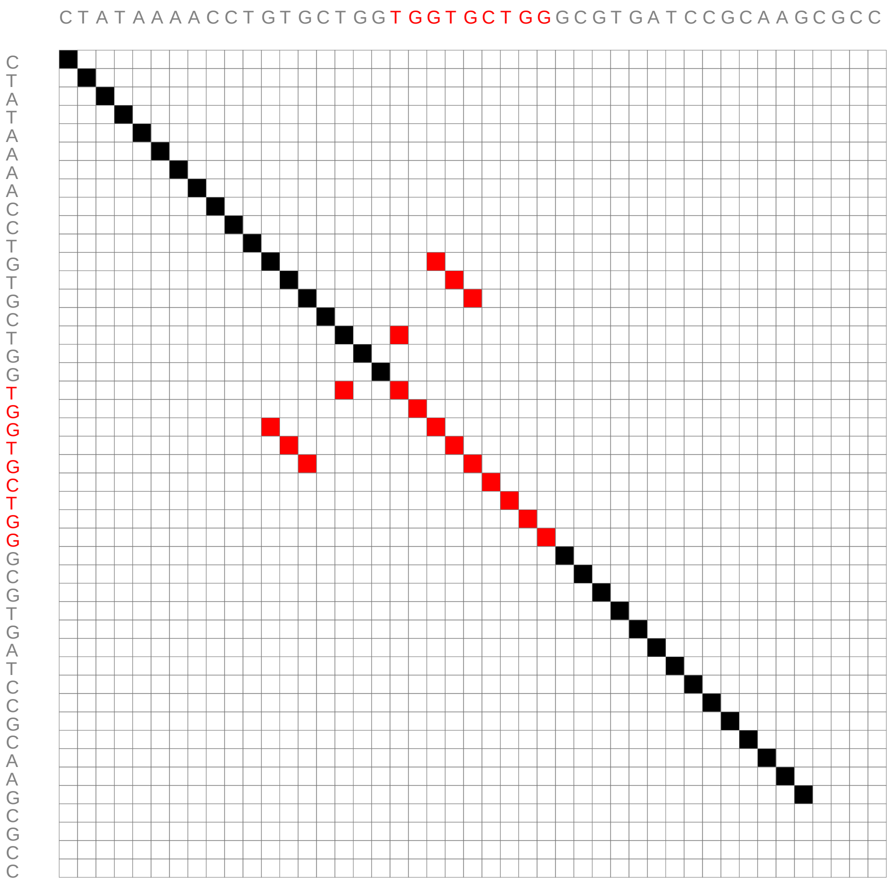

HP50

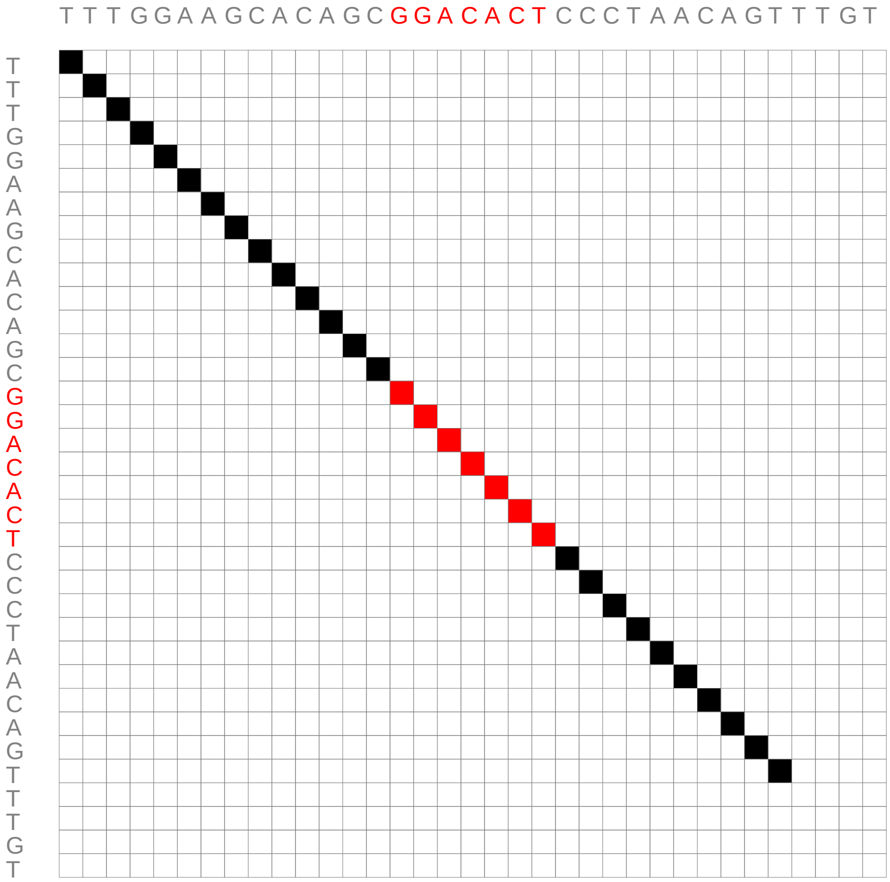
T T T G G A A G C A C A G C G G A C A C T C C C T A A C A G T T T G T
T T T G G A A G C A C A G C G G A C A C T C C C T A A C A G T T T G T

HP51

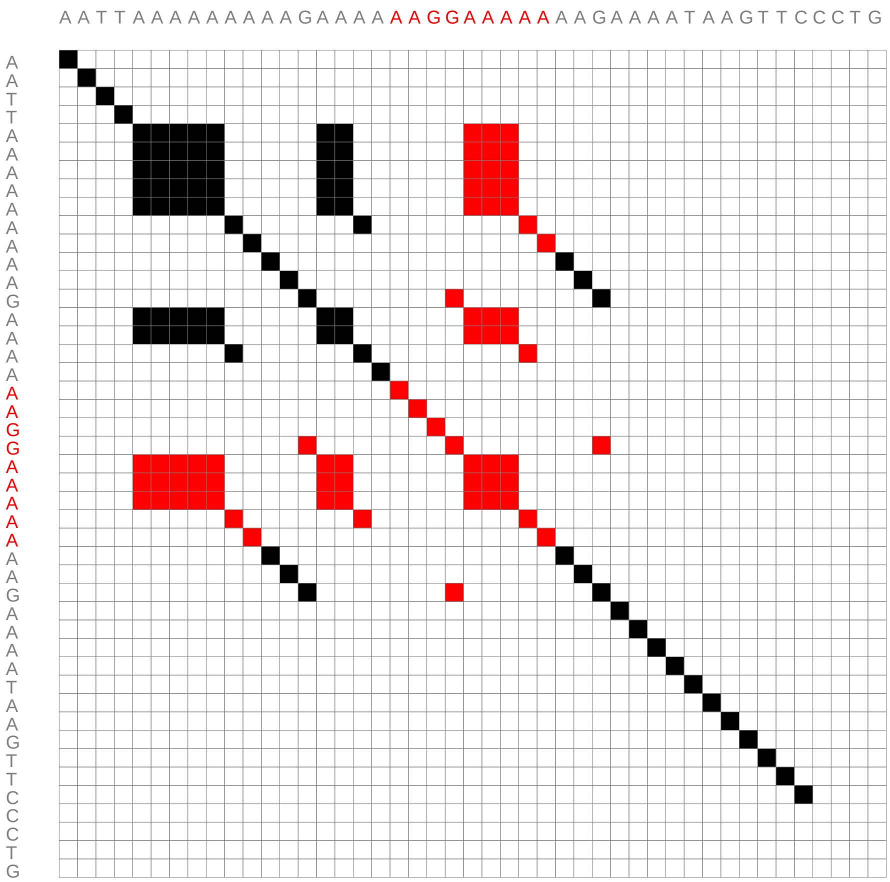

HP52

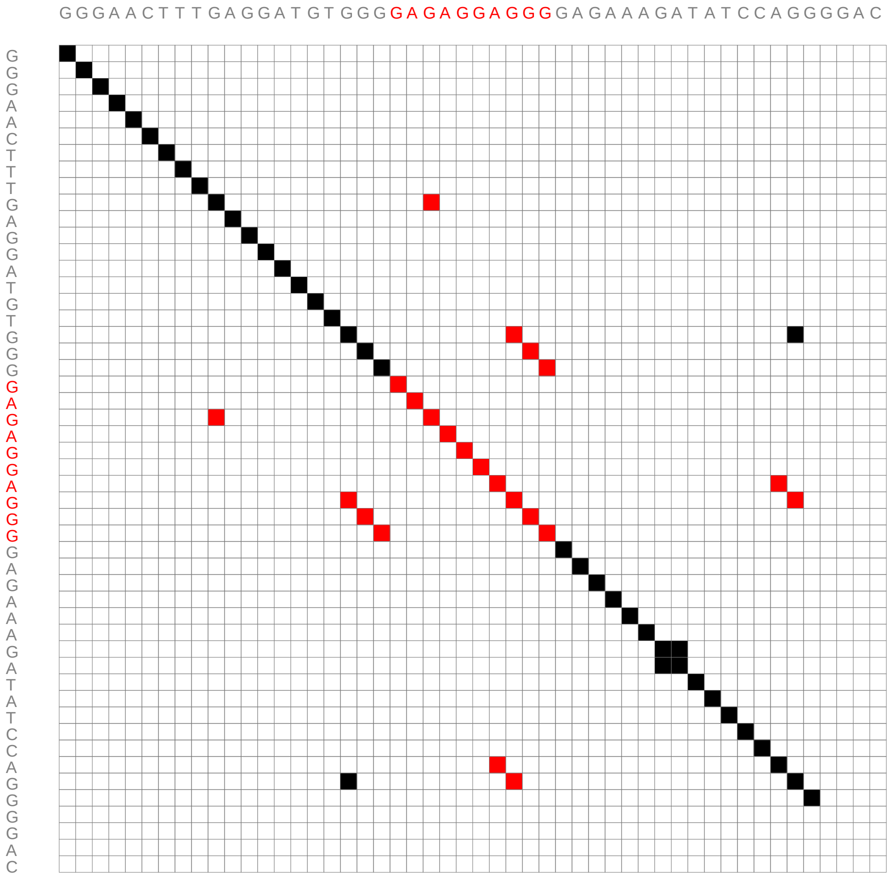
GGGAACTTTGAGGATGTGGGGAGAGGAGGGGAGAAAGATATCCAGGGGAC
GGGAACTTTGAGGATGTGGGGAGAGGAGGGGAGAAAGATATCCAGGGGAC

HP53

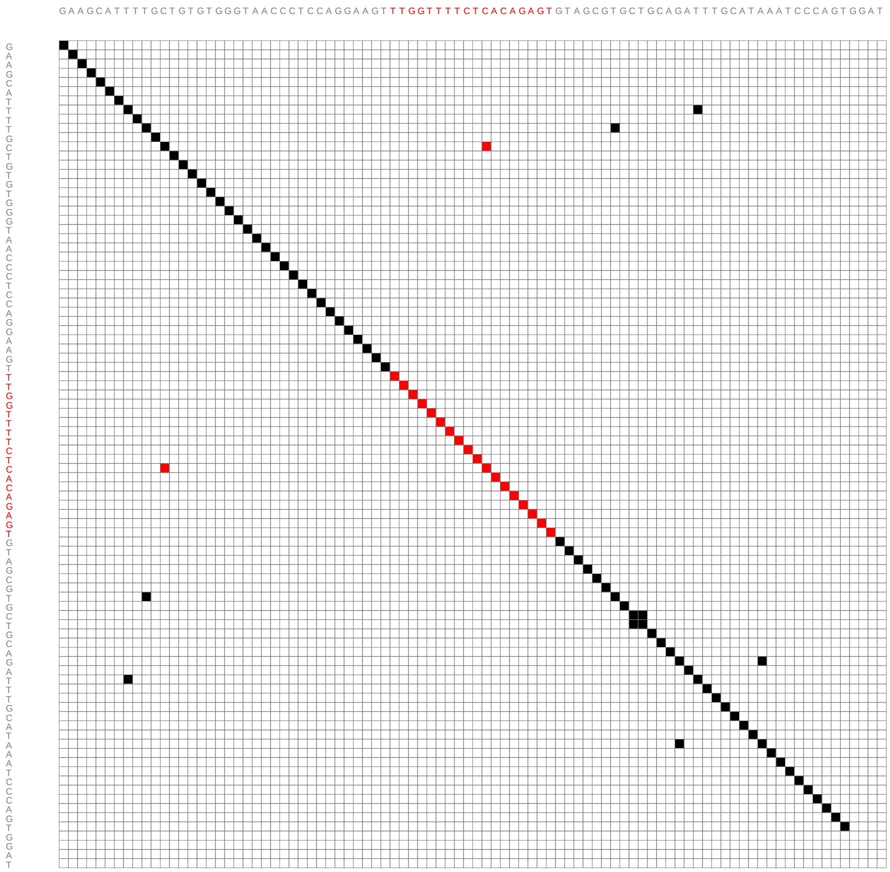

GAAGCATTTTGCTGTGTGGGTAACCCTCCAGGAAGTTTGGTTTTCTCACAGAGTGTAGCGTGCTGCAGATTTGCATAAATCCCAGTGGAT
GAAGCATTTTGCTGTGTGGGTAACCCTCCAGGAAGTTTGGTTTTCTCACAGAGTGTAGCGTGCTGCAGATTTGCATAAATCCCAGTGGAT

HP54

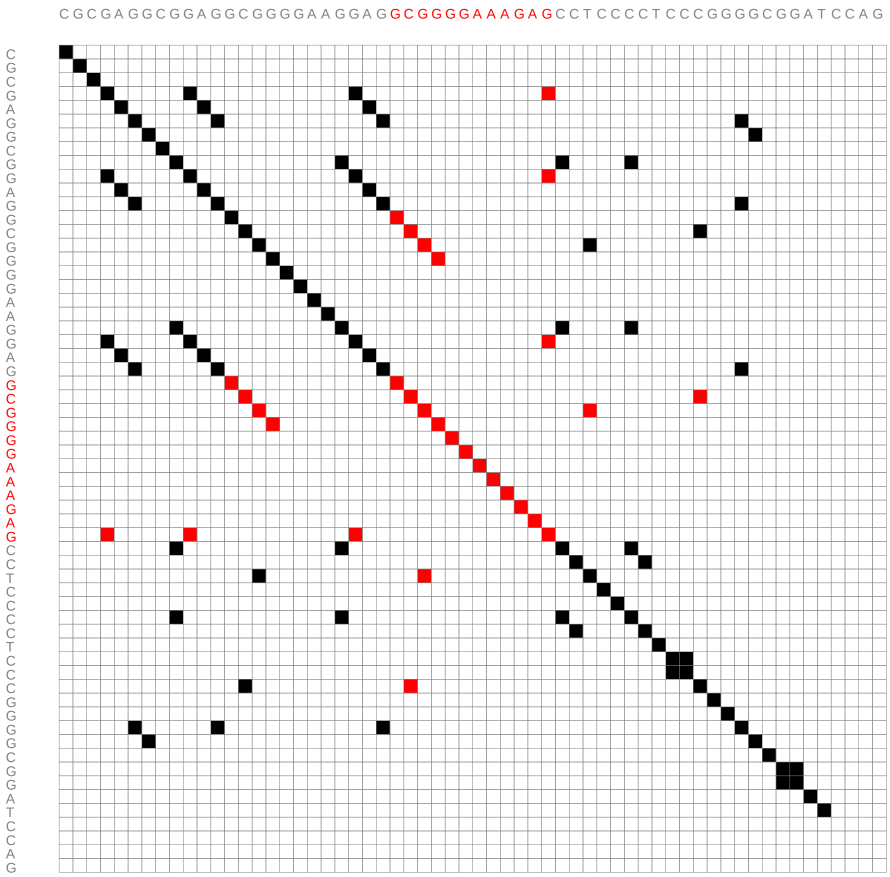
CGCGAGGCGGAGGCGGGGAAGGAGGCGGGGAAAGAGCCTCCCCTCCCGGGGCGGATCCAG

HP55

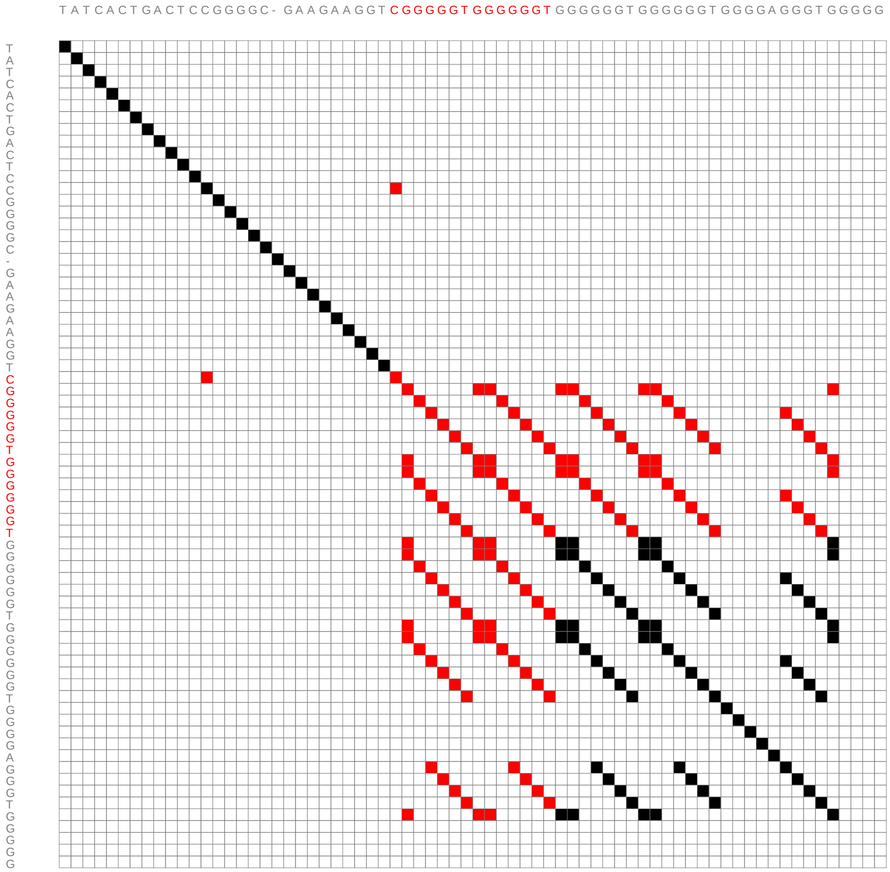
T A T C A C T G A C T C C G G G G C - G A A G A A G G T C G G G G G T G G G G G G T G G G G G G T G G G G G G T G G G G A G G G T G G G G G

HP56

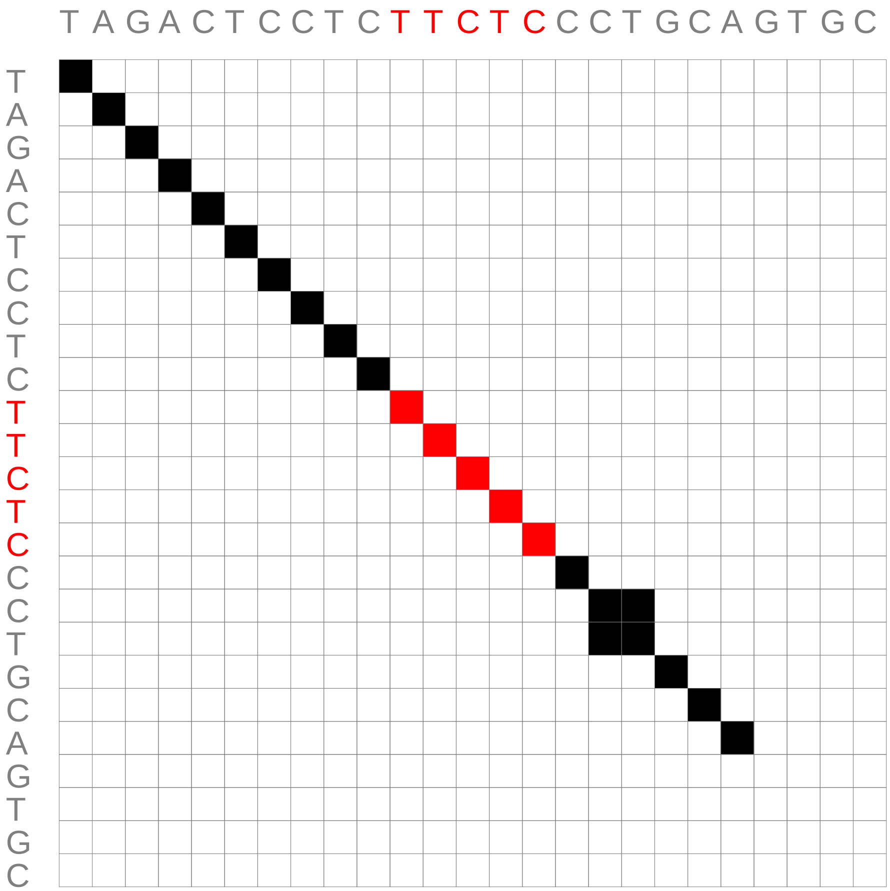
T A G A C T C C T C T T C T C C C T G C A G T G C
T A G A C T C C T C T T C T C C C T G C A G T G C

HP57

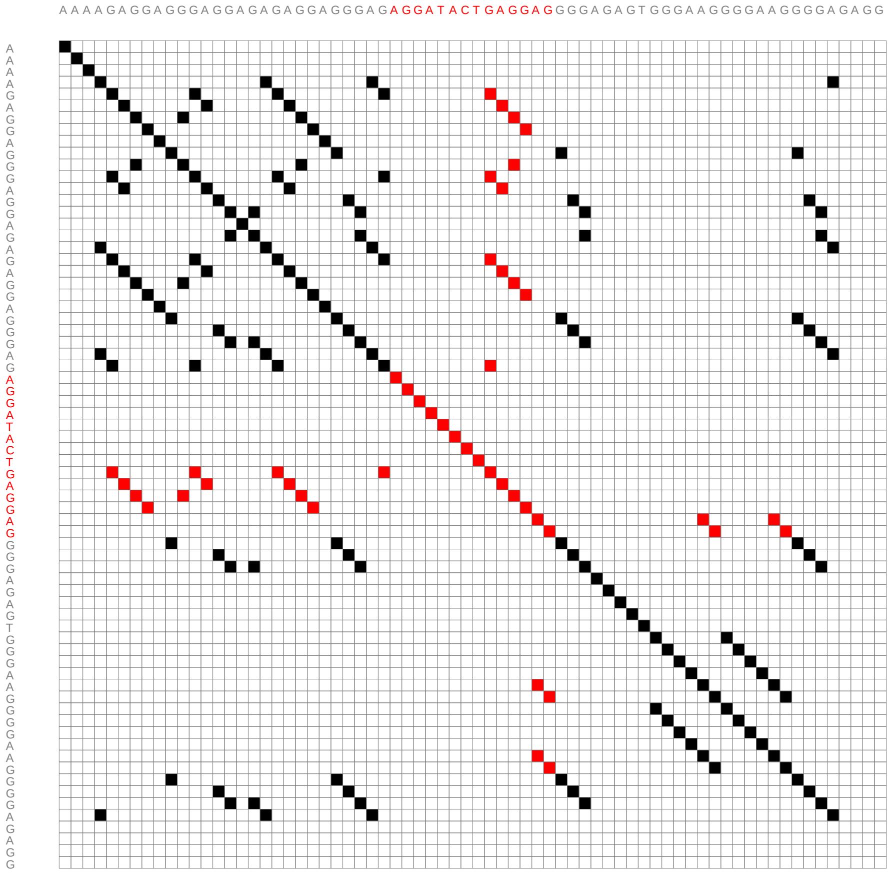
AAAAGAGGAGGGAGGAGAGAGGAGGGAGAGGATACTGAGGAGGGGAGAGTGGGAAGGGGAAGGGGAGAGG

HP58

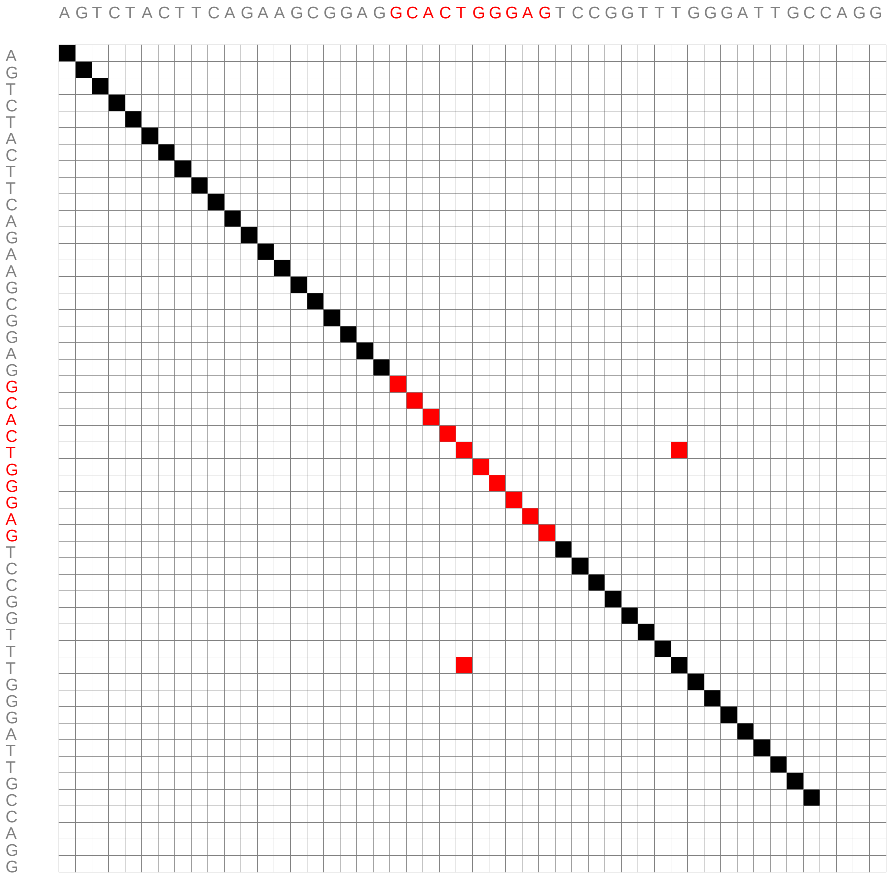

A G T C T A C T T C A G A A G C G G A G G C A C T G G G A G T C C G G T T T G G G A T T G C C A G G
A G T C T A C T T C A G A A G C G G A G G C A C T G G G A G T C C G G T T T G G G A T T G C C A G G

HP59

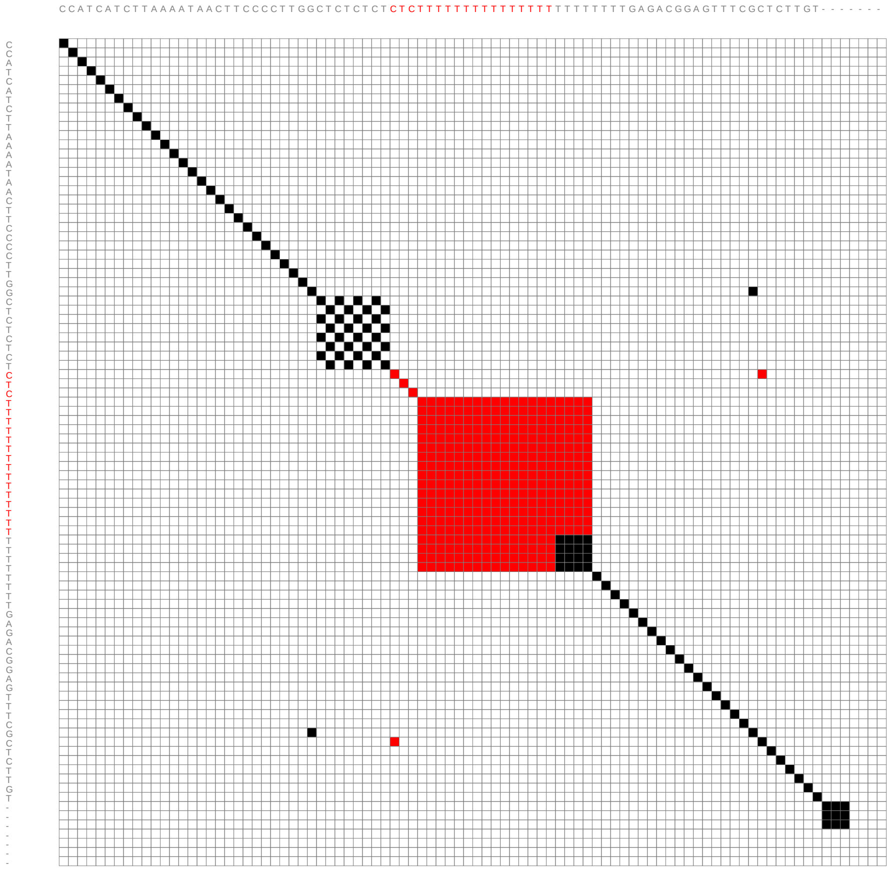

CCATCATCTTAAAATAACTTCCCCTTGGCTCTCTCTCTCTTTTTTTTTTTTTTTTTTTTTTTGAGACGGAGTTTCGCTCTTGT-------

HP60

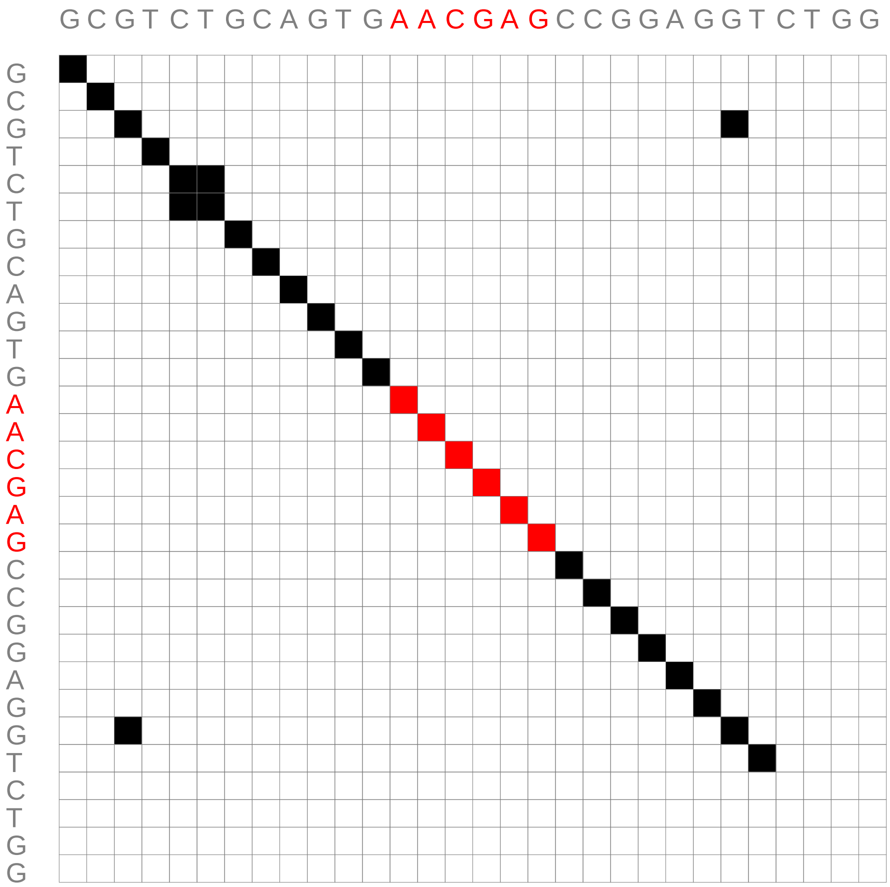
G C G T C T G C A G T G A A C G A G C C G G A G G T C T G G
G C G T C T G C A G T G A A C G A G C C G G A G G T C T G G

HP61

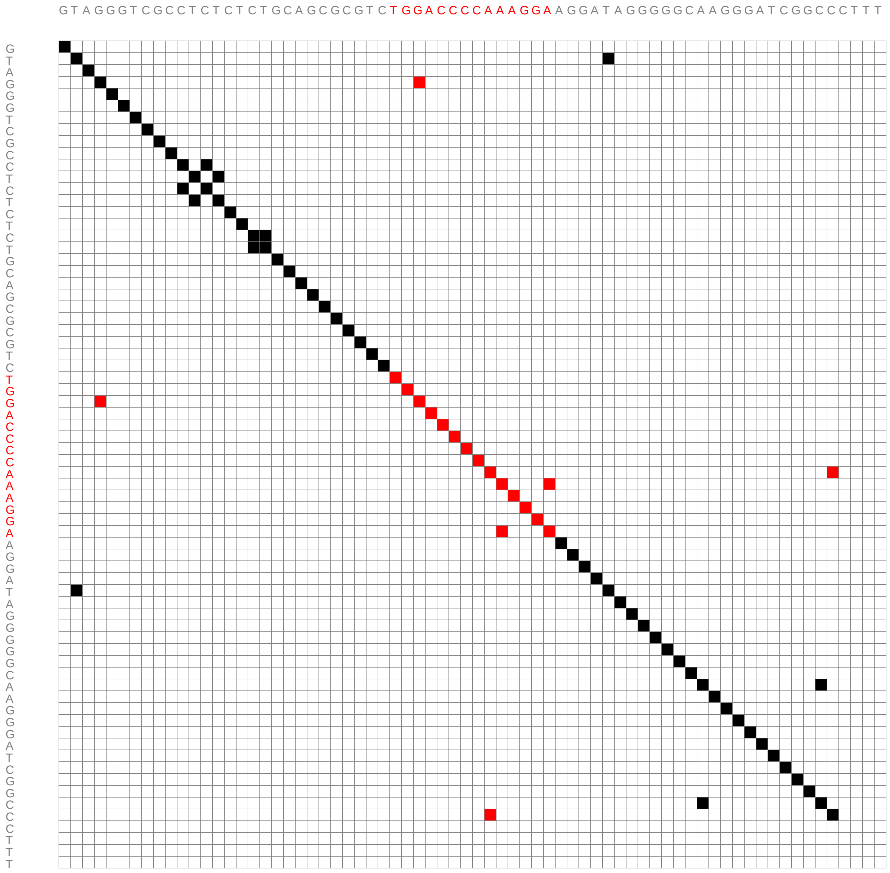
GTAGGGTCGCCTCTCTCTGCAGCGCGTCTGGACCCCAAAGGAAGGATAGGGGGCAAGGGATCGGCCCTTT

HP62

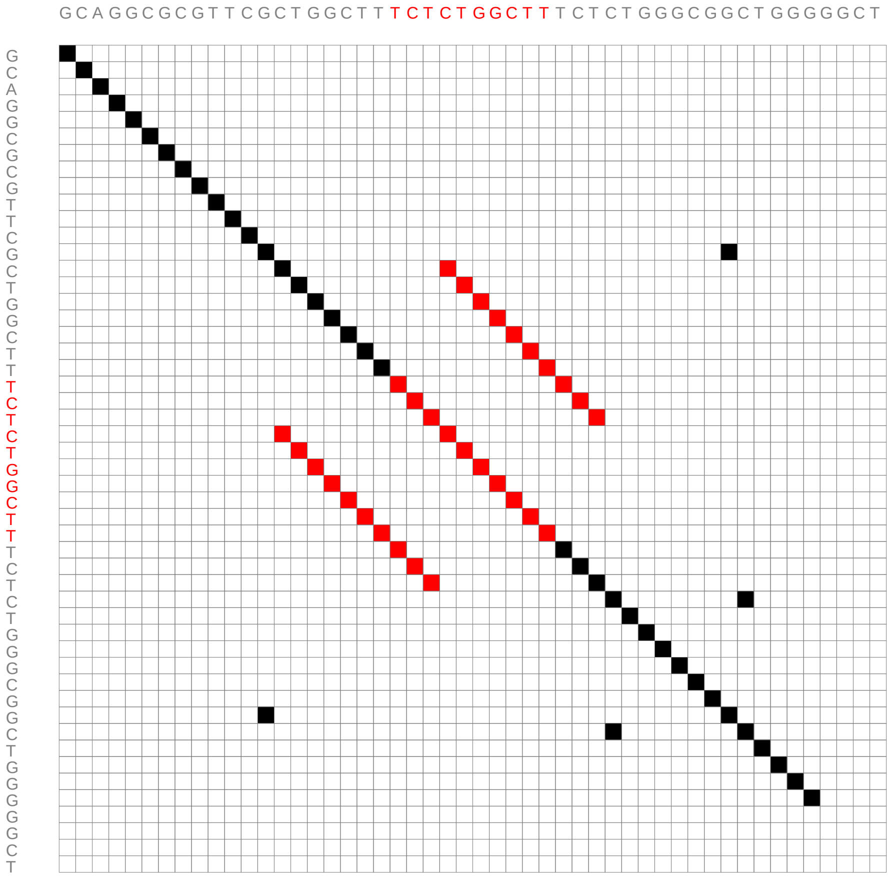

G C A G G C G C G T T C G C T G G C T T T C T C T G G C T T T C T C T G G G C G G C T G G G G G C T

HP63

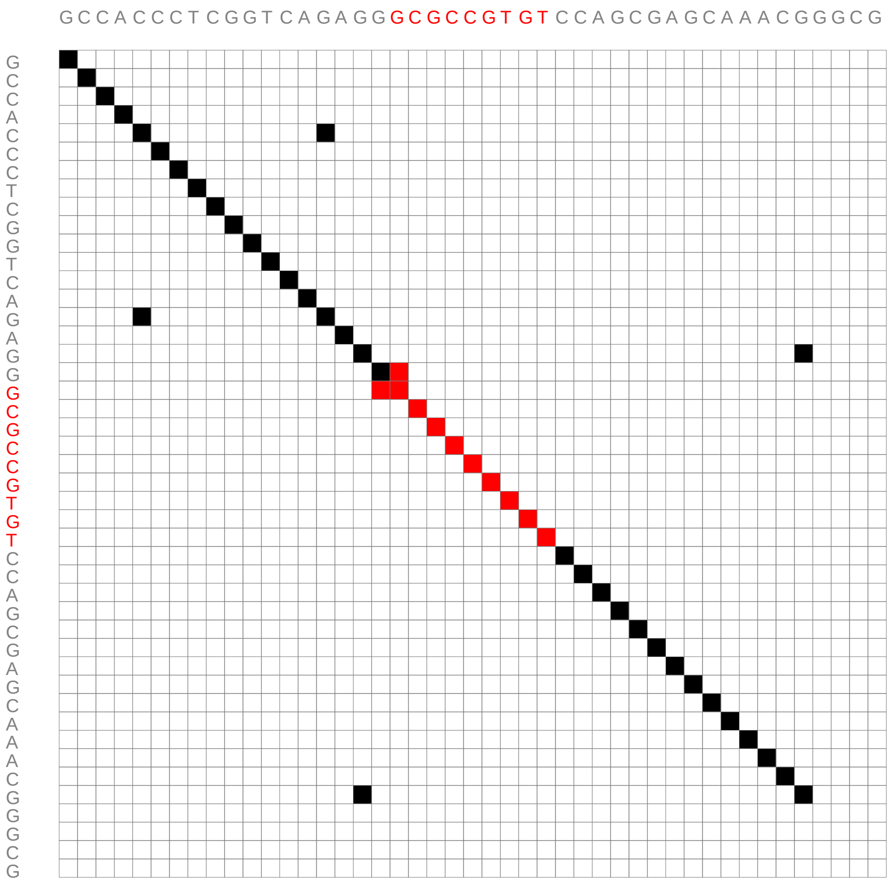
GCCACCCTCGGTCAGAGGGCGCCGTGTCCAGCGAGCAAACGGGCG
GCCACCCTCGGTCAGAGGGCGCCGTGTCCAGCGAGCAAACGGGCG

HP64

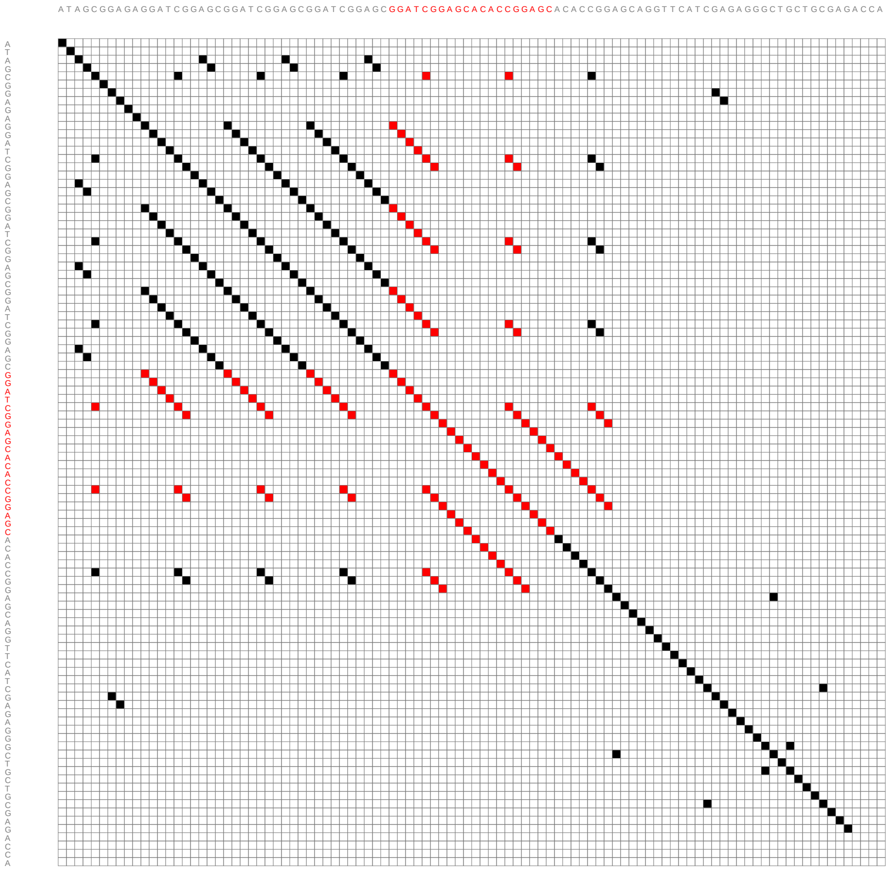
ATAGCGGAGAGGATCGGAGCGGATCGGAGCGGATCGGAGCGGATCGGAGCACACCGGAGCACACCGGAGCAGGTTCATCGAGAGGGCTGCTGCGAGACCA

HP65

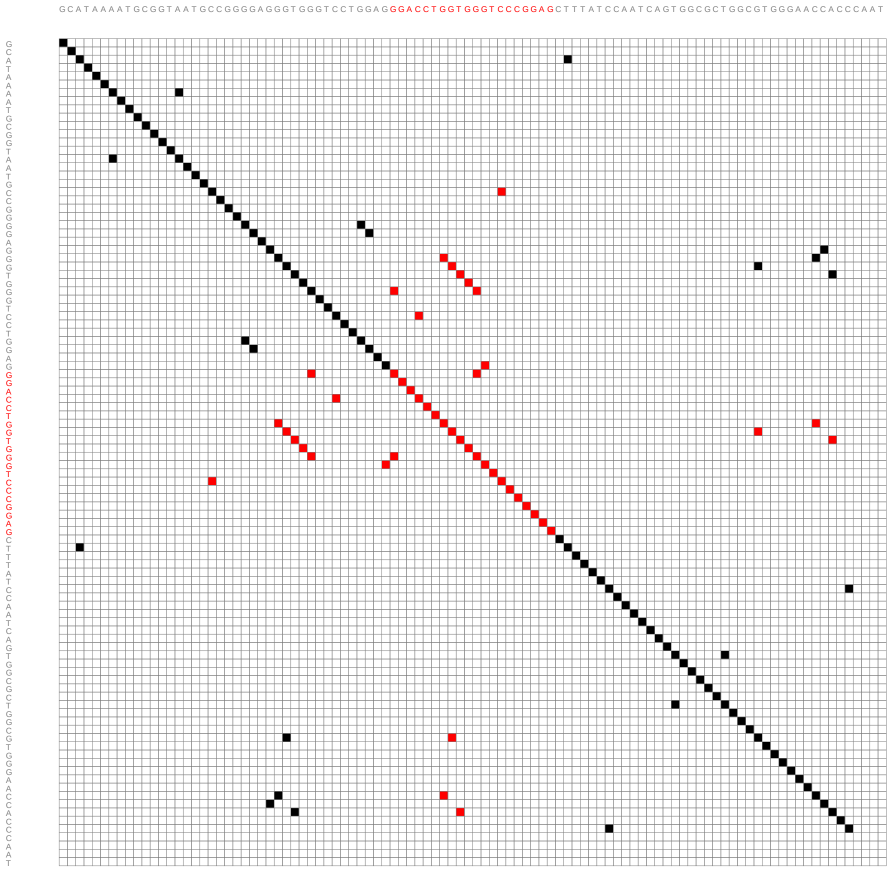

GCATAAAATGCGGTAATGCCGGGGAGGGTGGGTCCTGGAGGGACCTGGTGGGTCCCGGAGCTTTATCCAATCAGTGGCGCTGGCGTGGGAACCACCCAAT

HP66

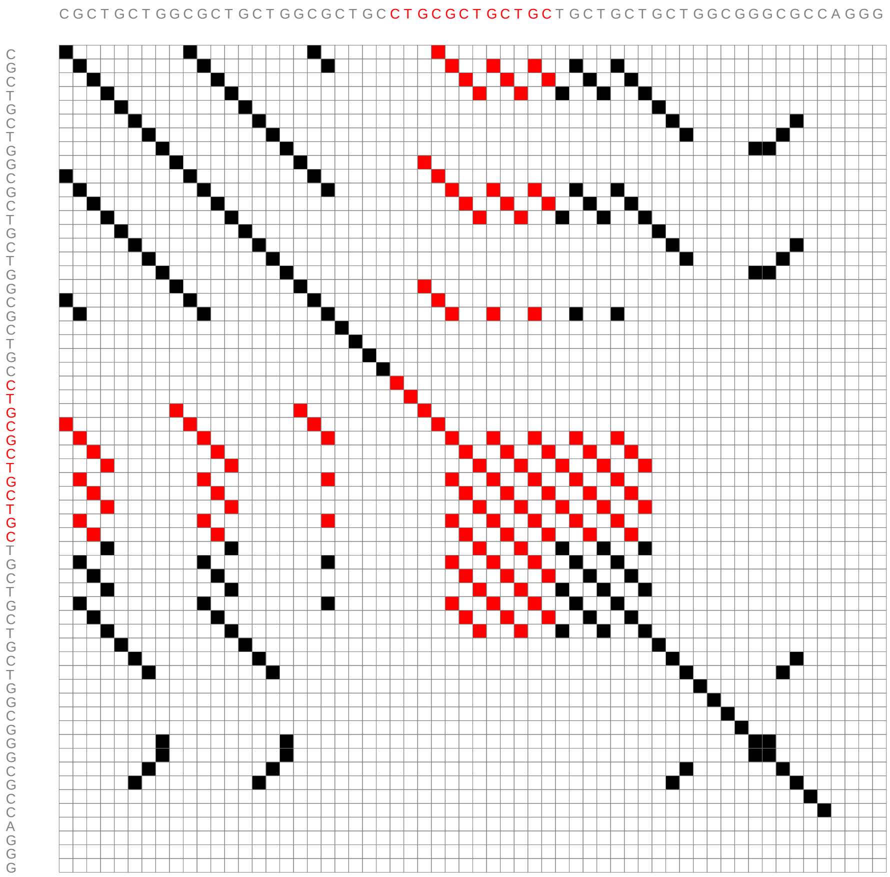
C G C T G C T G G C G C T G C T G G C G C T G C C T G C G C T G C T G C T G C T G C T G C T G G C G G G C G C C A G G G

HP67

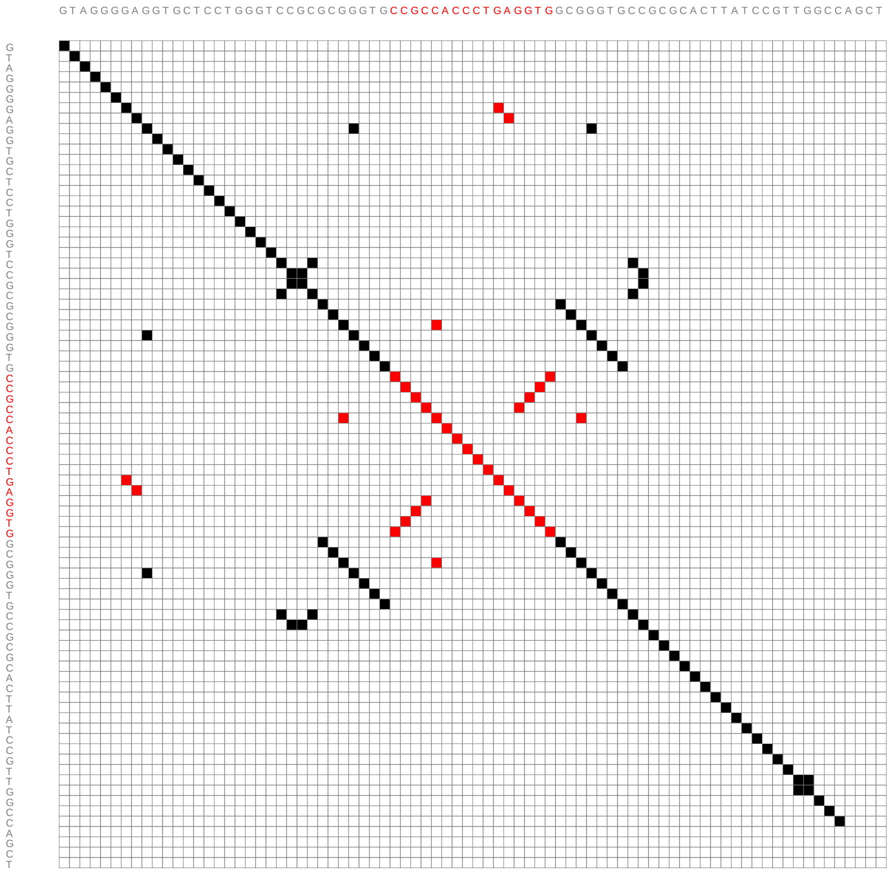
GTAGGGGAGGTGCTCCTGGGTCCGCGCGGGTGCCGCCACCCTGAGGTGGCGGGTGCCGCGCACTTATCCGTTGGCCAGCT
GTAGGGGAGGTGCTCCTGGGTCCGCGCGGGTGCCGCCACCCTGAGGTGGCGGGTGCCGCGCACTTATCCGTTGGCCAGCT

HP68

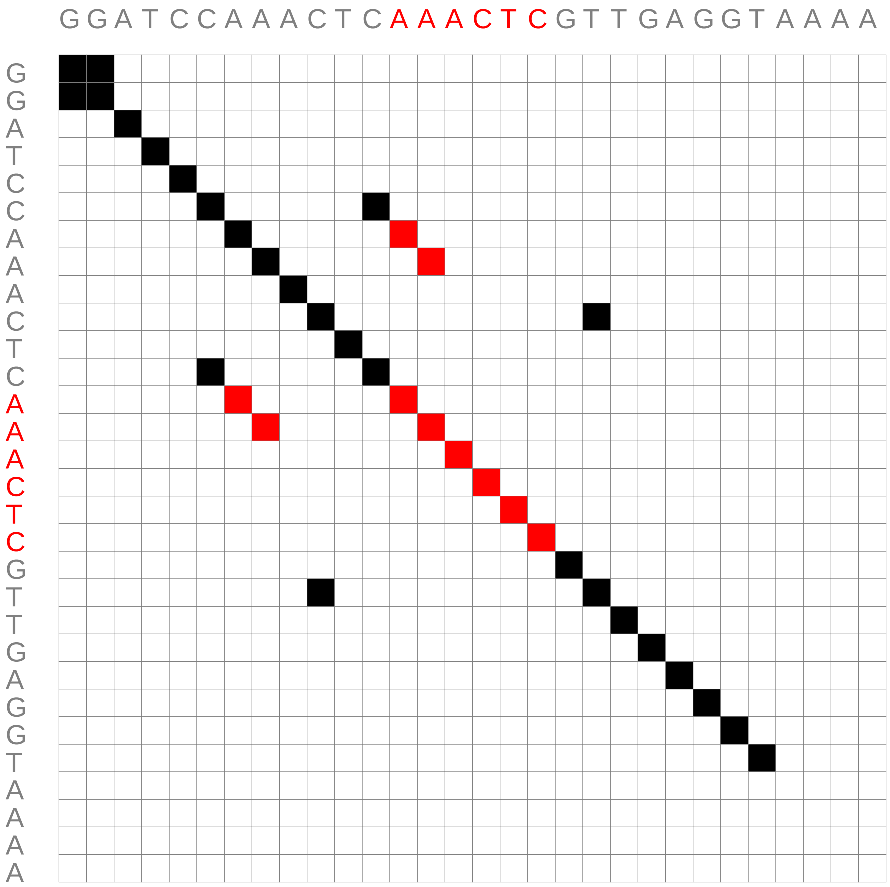
G G A T C C A A A C T C A A A C T C G T T G A G G T A A A A
G G A T C C A A A C T C A A A C T C G T T G A G G T A A A A

HP69

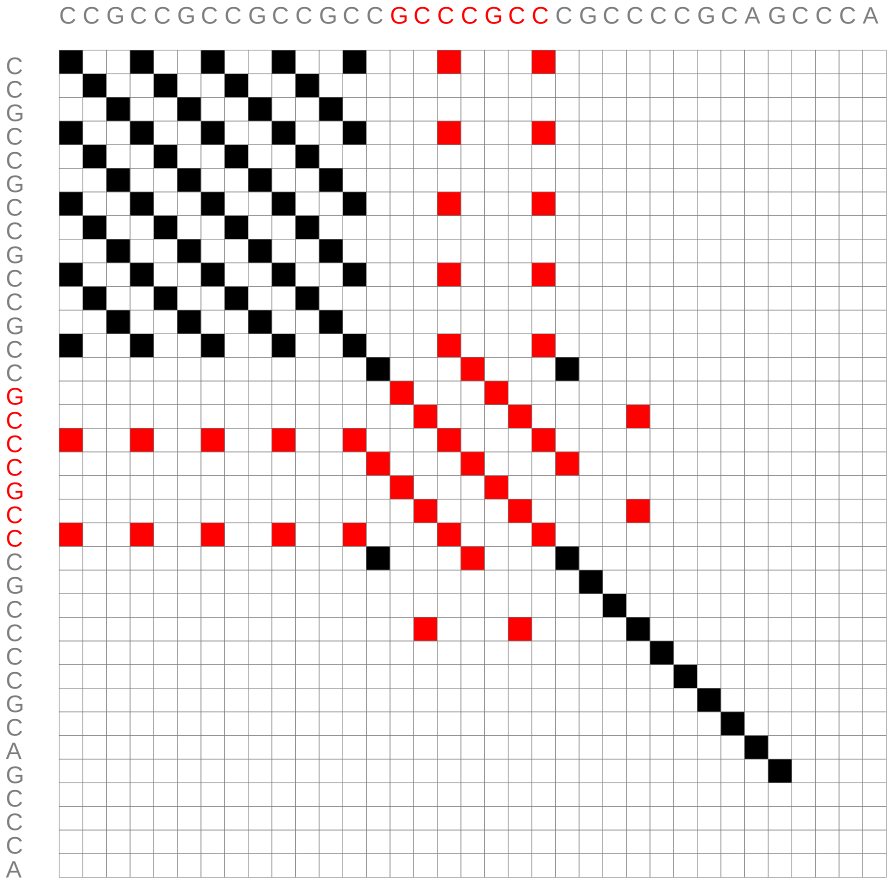

C C G C C G C C G C C G C C G C C C G C C C G C C C C G C A G C C C A
C C G C C G C C G C C G C C G C C C G C C C G C C C C G C A G C C C A

HP70

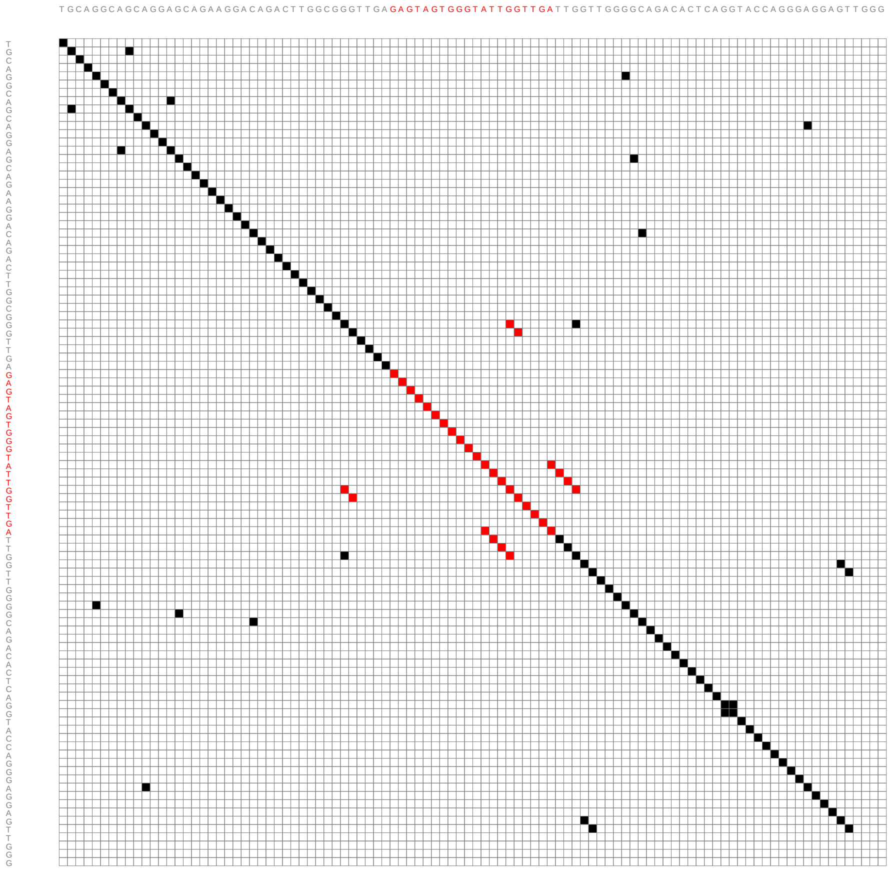

HP71

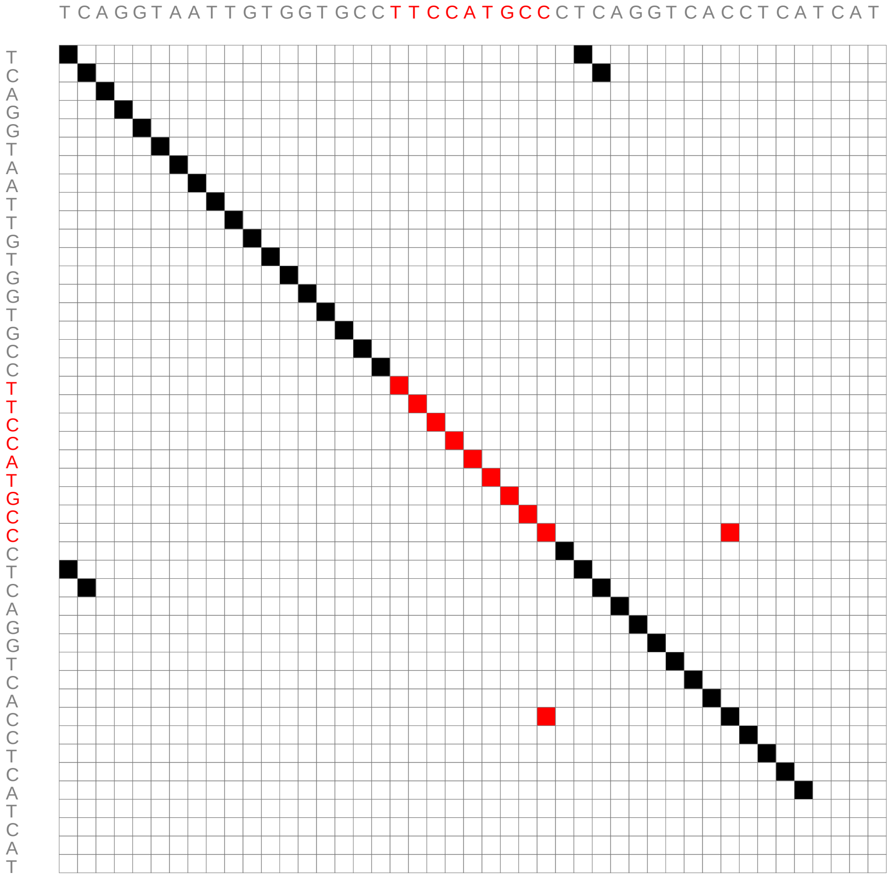

TCAGGTAATTGTGGTGCCTTCCATGCCCTCAGGTCACCTCATCAT
TCAGGTAATTGTGGTGCCTTCCATGCCCTCAGGTCACCTCATCAT

HP72

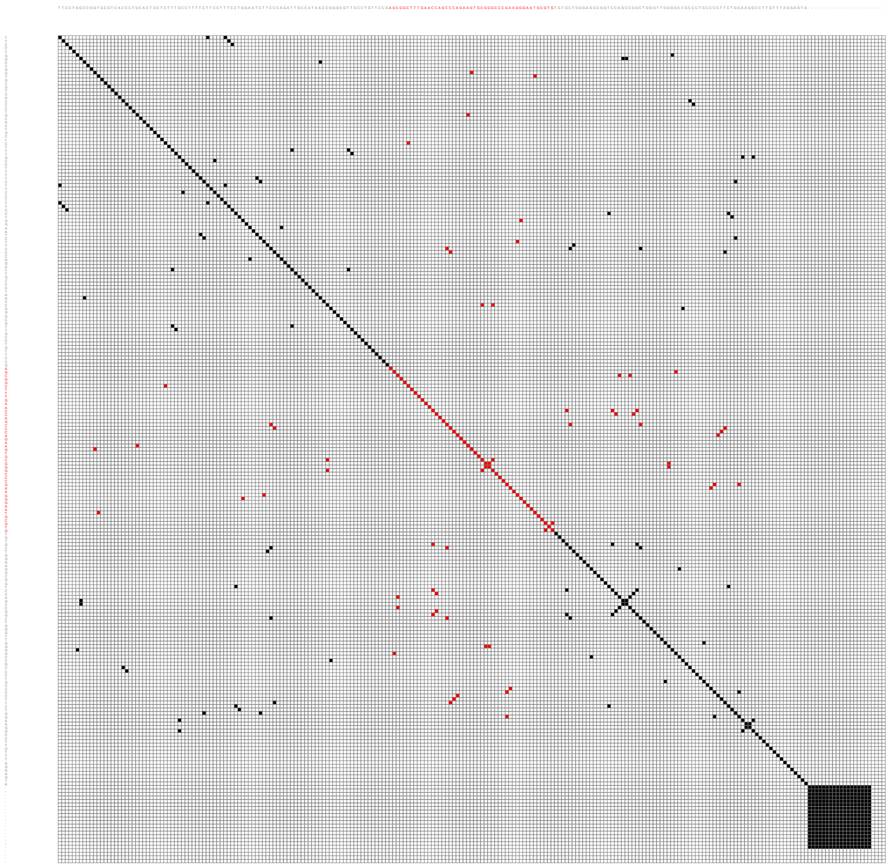

HP73

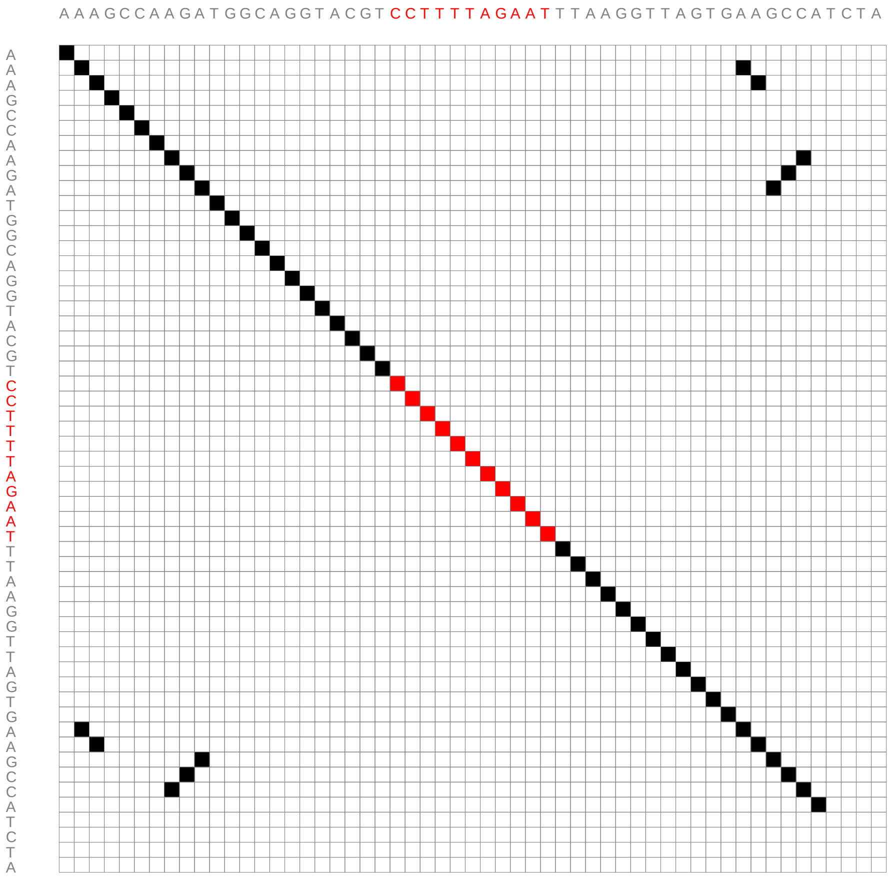
A A A G C C A A G A T G G C A G G T A C G T C C T T T T A G A A T T T A A G G T T A G T G A A G C C A T C T A

HP74

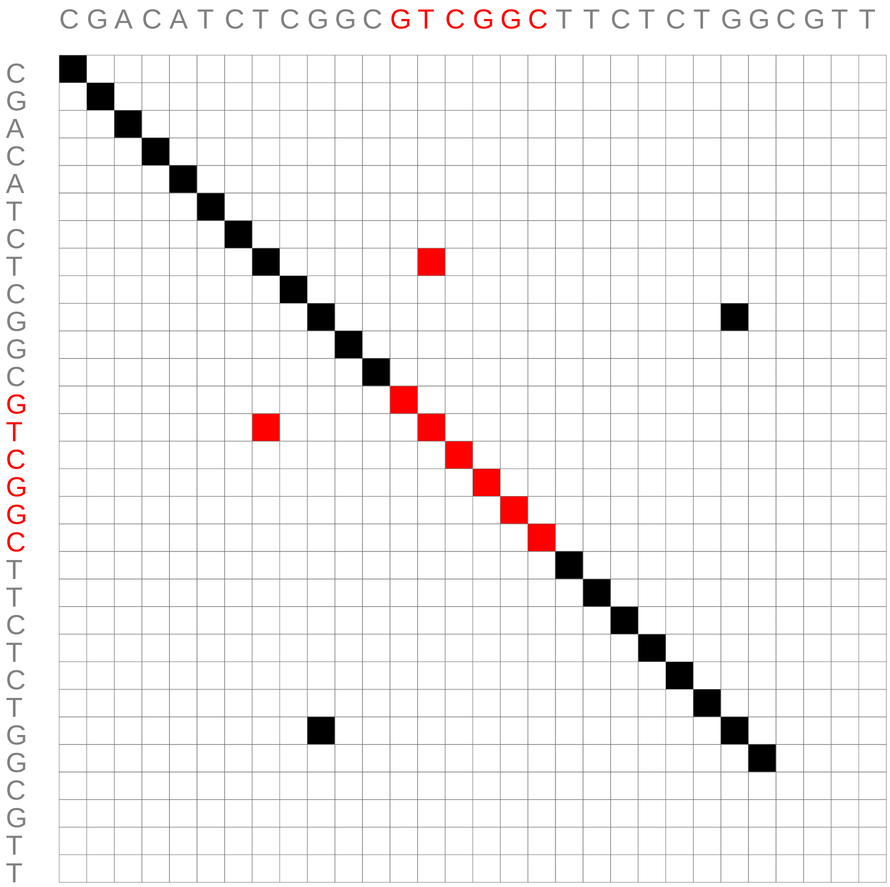
C G A C A T C T C G G C G T C G G C T T C T C T G G C G T T
C G A C A T C T C G G C G T C G G C T T C T C T G G C G T T

HP75

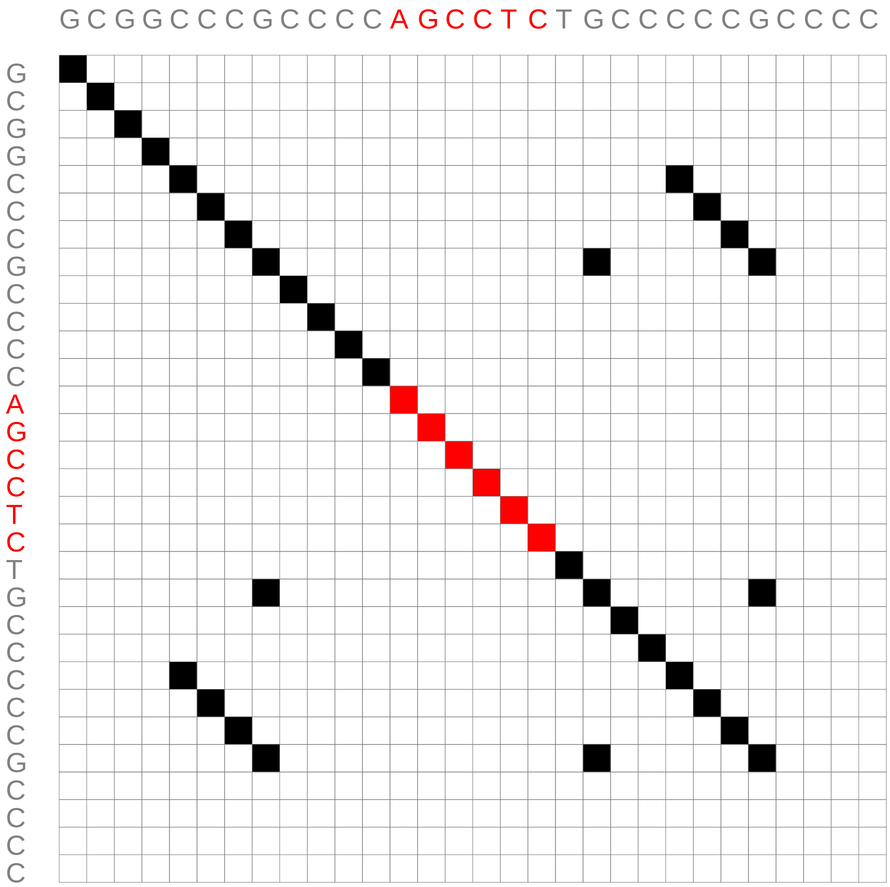
G C G G C C C G C C C C A G C C T C T G C C C C C G C C C C
G C G G C C C G C C C C A G C C T C T G C C C C C G C C C C

HP76

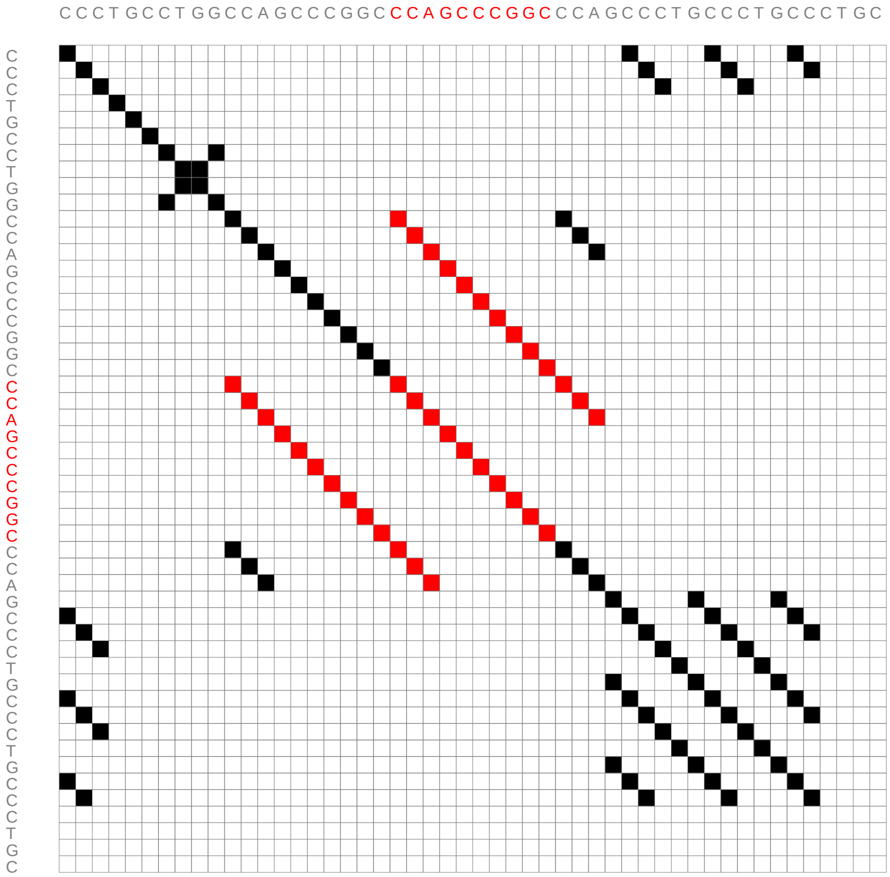
C C C T G C C T G G C C A G C C C G G C C C A G C C C G G C C C A G C C C T G C C C T G C C C T G C

HP77

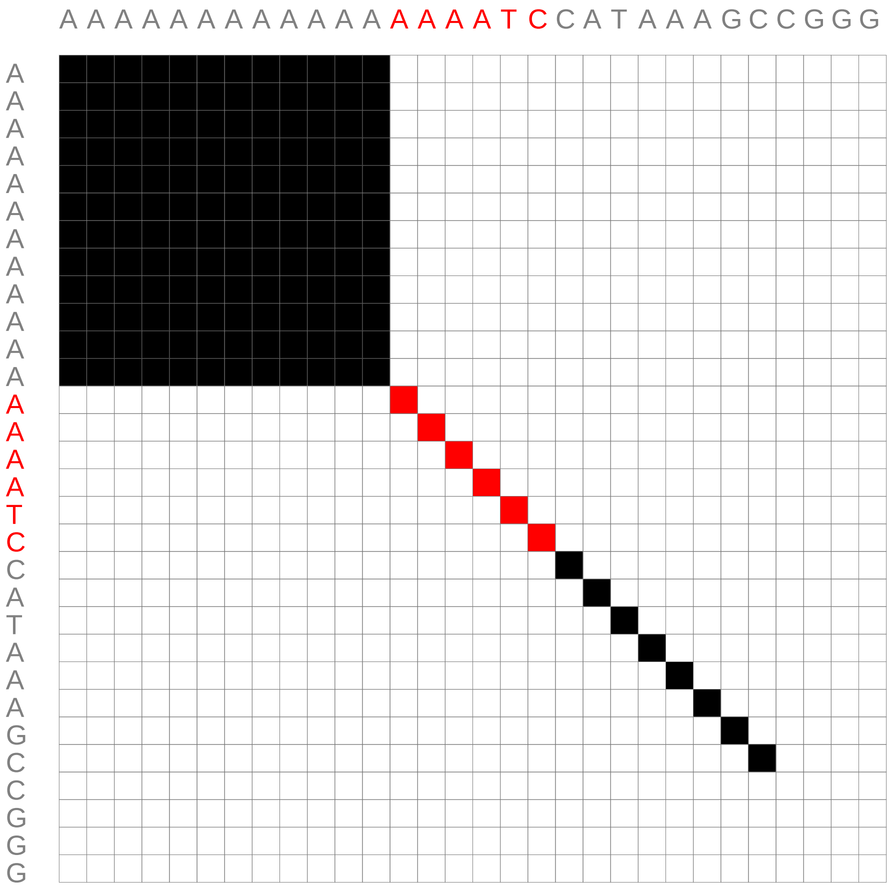
A A A A A A A A A A A A A A A A T C C A T A A A G C C G G G
A A A A A A A A A A A A A A A A T C C A T A A A G C C G G G

HP78

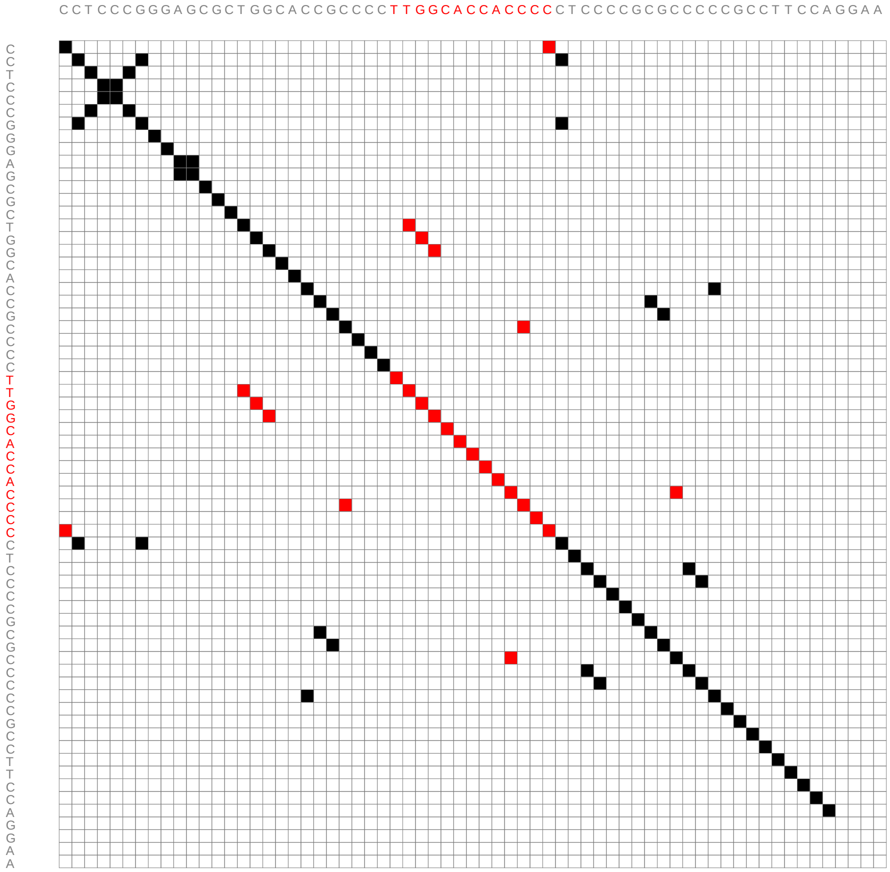
CCTCCCGGGAGCGCTGGCACCGCCCCTTGGCACCACCCCCTCCCCGCGCCCCCGCCTTCCAGGAA

HP79

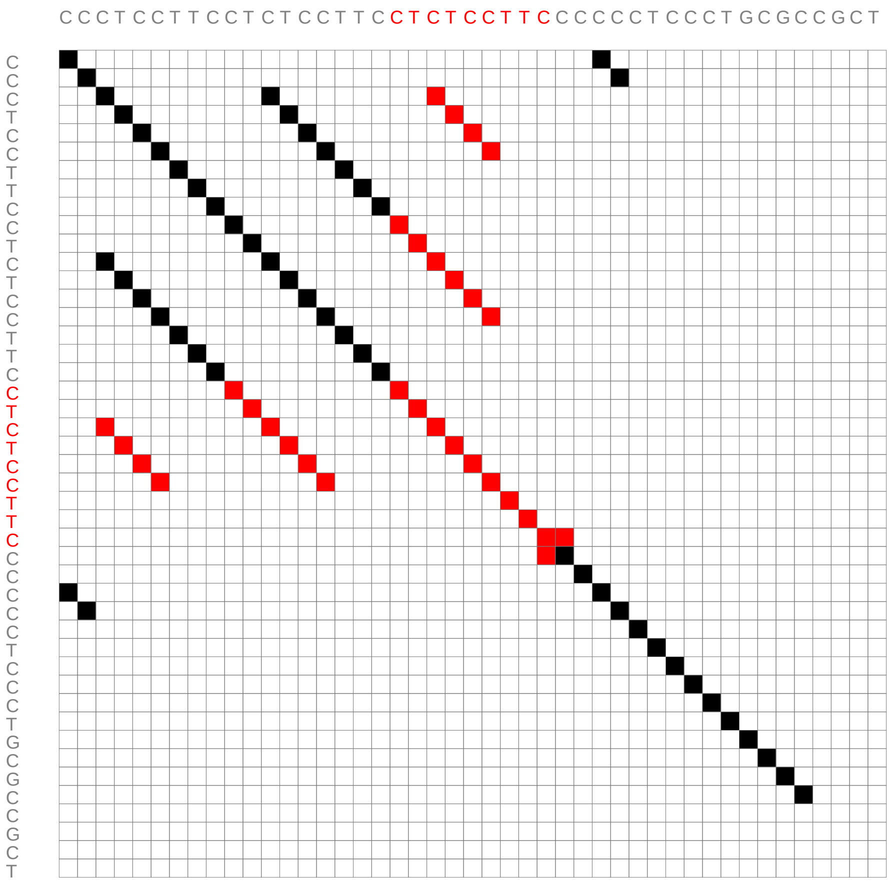
C C C T C C T T C C T C T C C T T C C T C T C C T T C C C C C C T C C C T G C G C C G C T
C C C T C C T T C C T C T C C T T C C T C T C C T T C C C C C C T C C C T G C G C C G C T

HP80

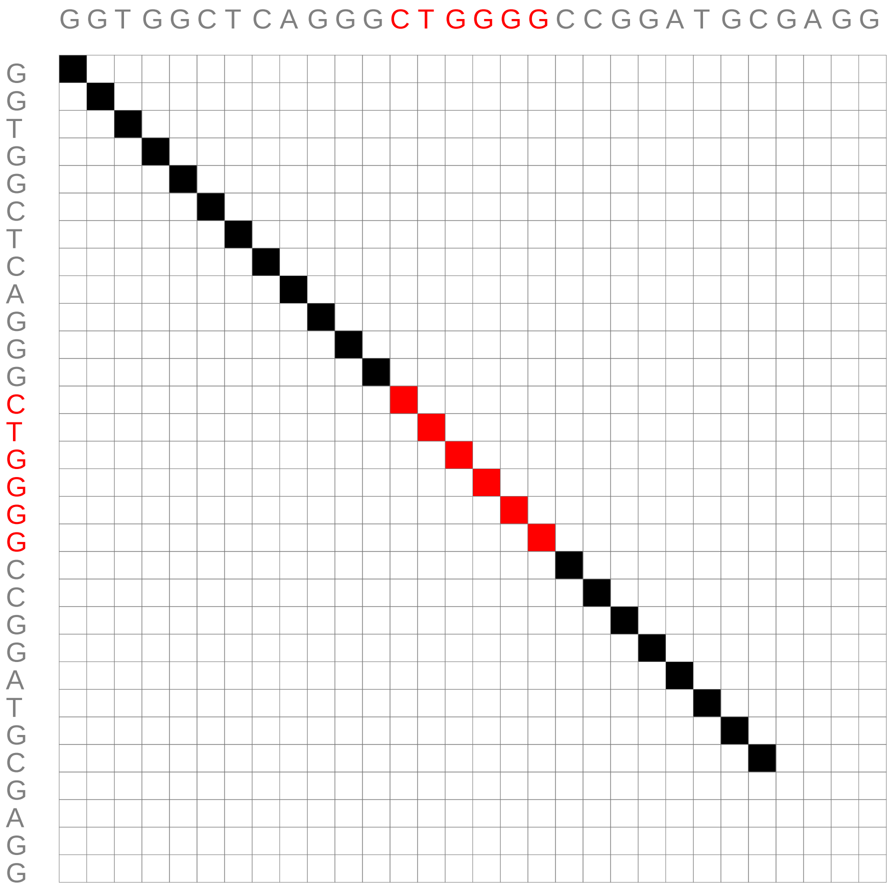

G G T G G C T C A G G G C T G G G G C C G G A T G C G A G G
G G T G G C T C A G G G C T G G G G C C G G A T G C G A G G

HP81

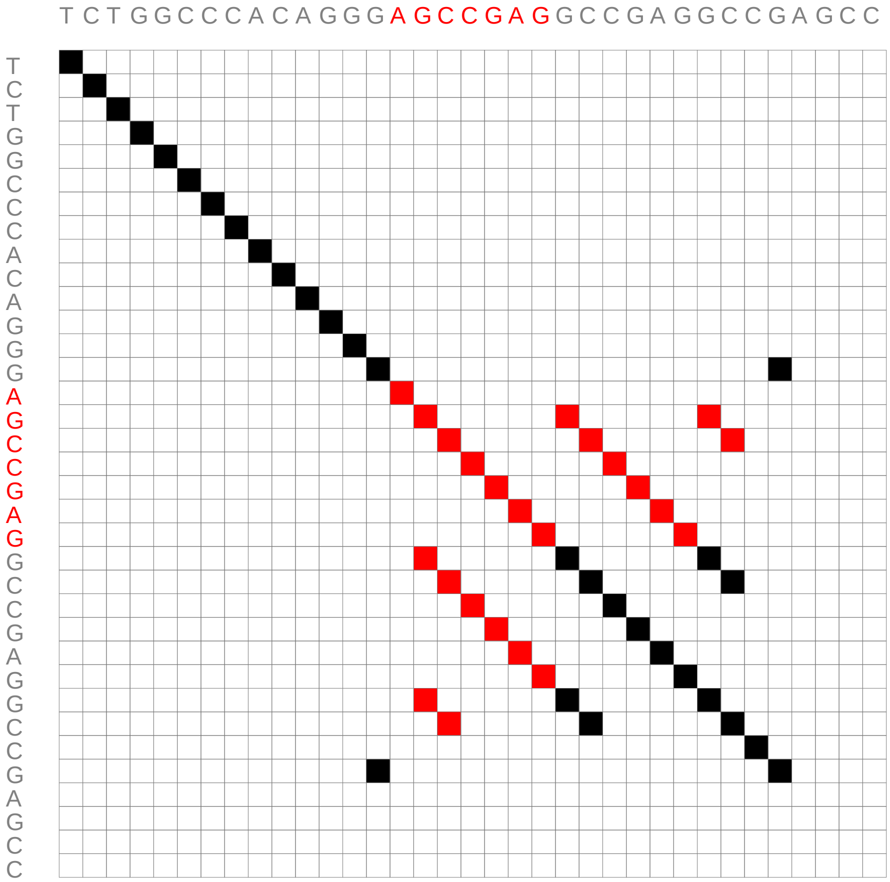

T C T G G C C C A C A G G G A G C C G A G G C C G A G G C C G A G C C
T C T G G C C C A C A G G G A G C C G A G G C C G A G G C C G A G C C

HP82

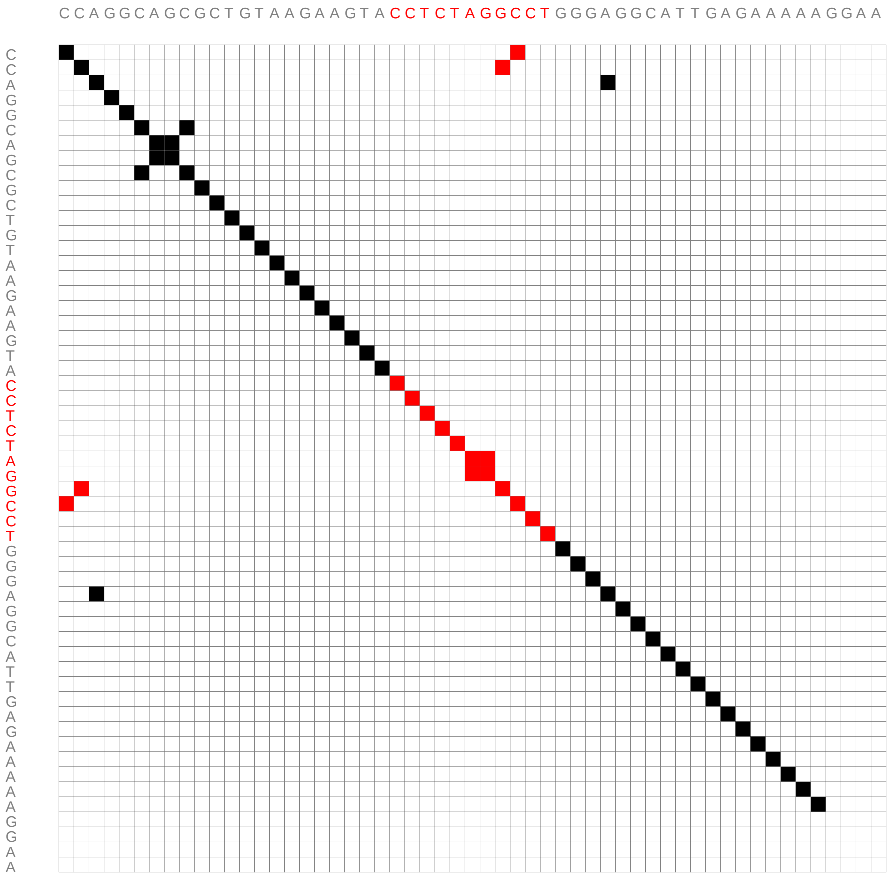
CCAGGCAGCGCTGTAAGAAGTACCTCTAGGCCTGGGAGGCATTGAGAAAAAGGAA
CCAGGCAGCGCTGTAAGAAGTACCTCTAGGCCTGGGAGGCATTGAGAAAAAGGAA

HP83

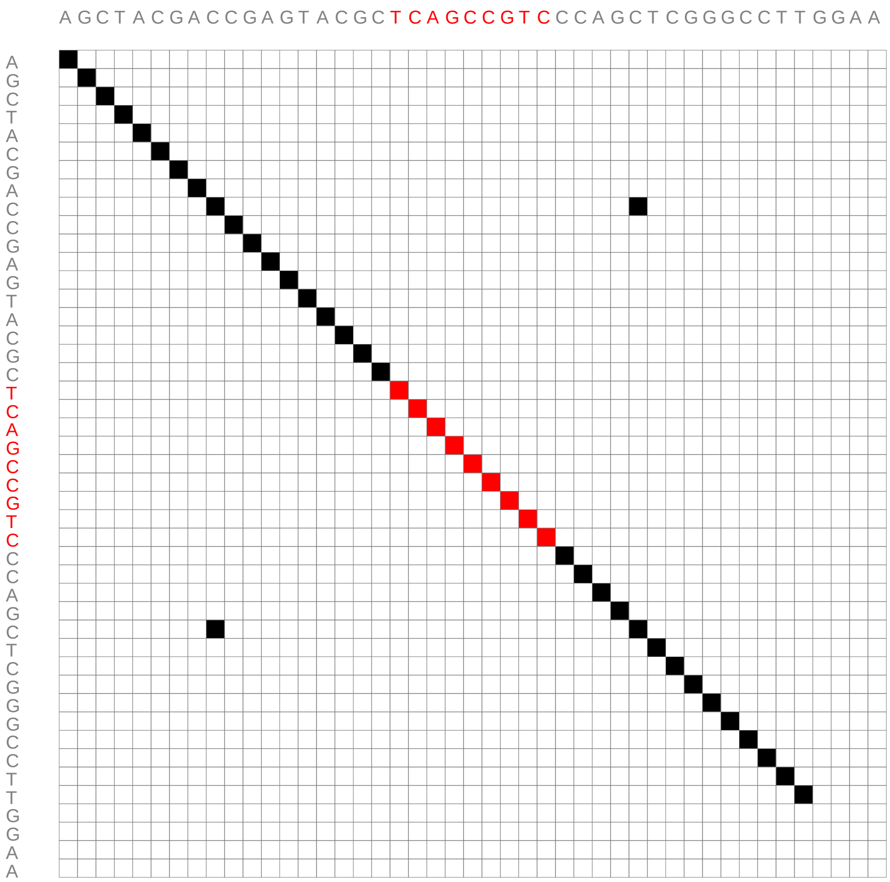

HP84

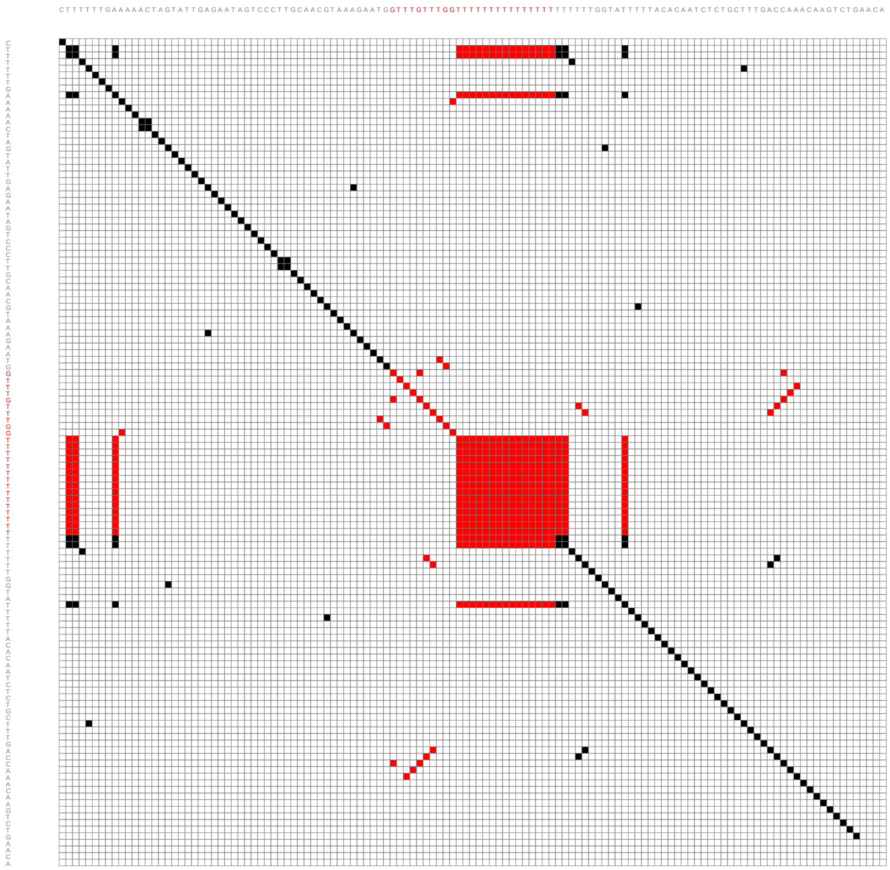

C T T T T T T G A A A A A C T A G T A T T G A G A A T A G T C C C T T G C A A C G T A A A G A A T G G T T T G T T T G G T T T T T T T T T T T T T T T T T T T T T G G T A T T T T T A C A C A A T C T C T G C T T T G A C C A A A C A A G T C T G A A C A

HP85

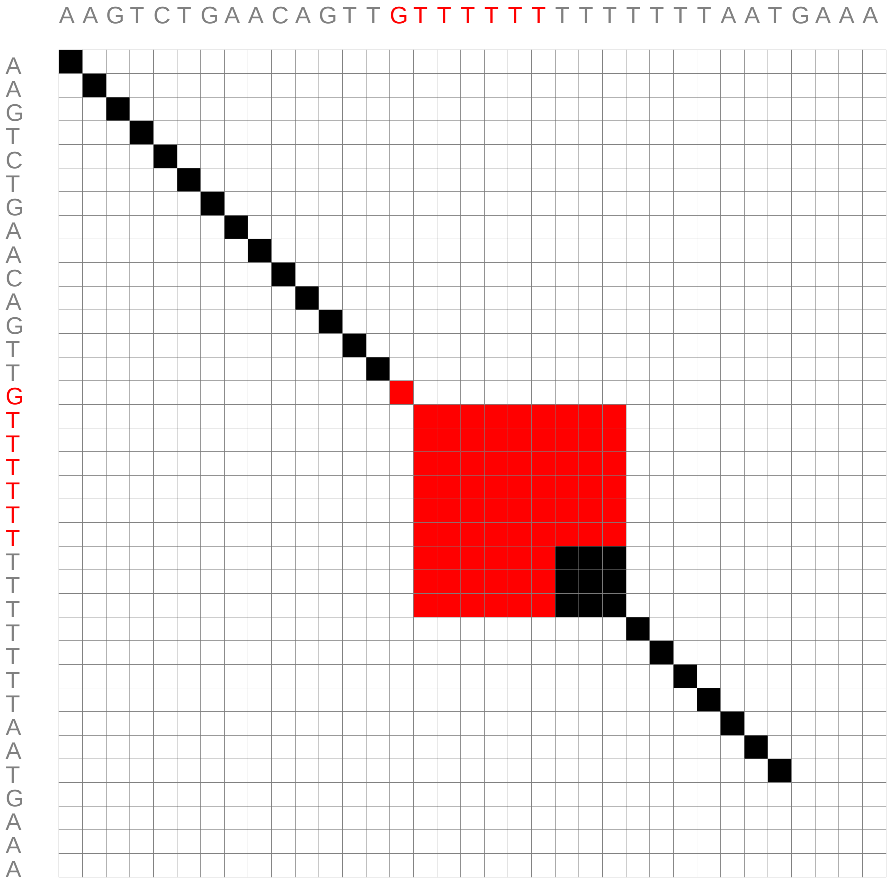

A A G T C T G A A C A G T T G T T T T T T T T T T T T T T A A T G A A A
A A G T C T G A A C A G T T G T T T T T T T T T T T T T T A A T G A A A

HP86

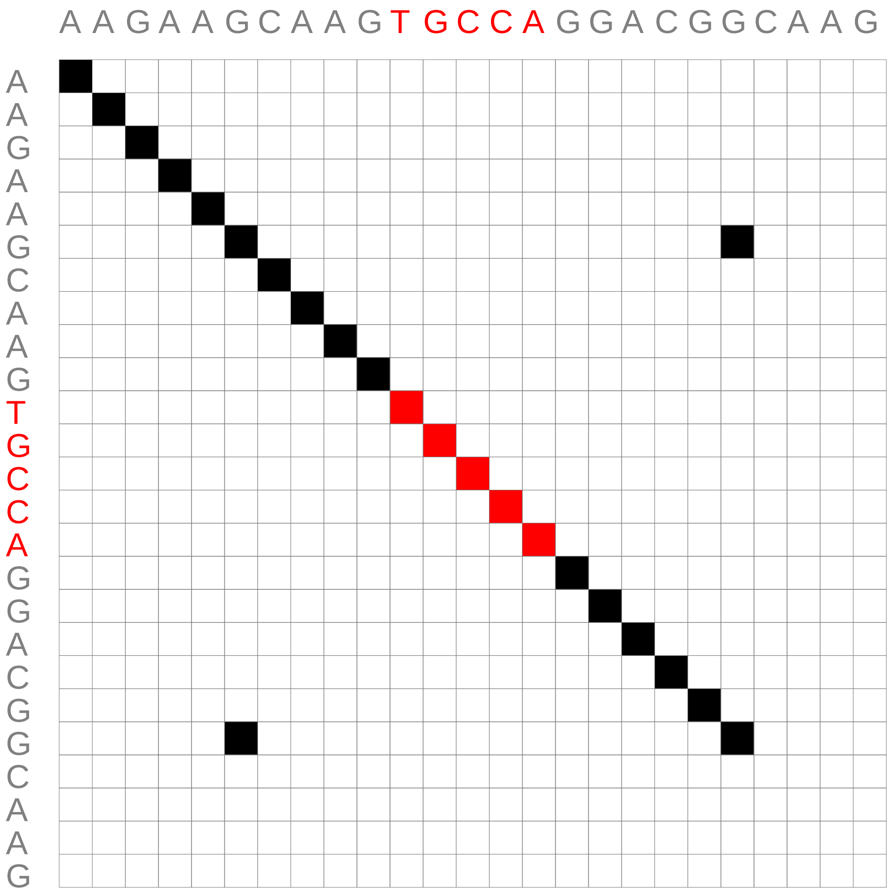
A A G A A G C A A G T G C C A G G A C G G C A A G
A A G A A G C A A G T G C C A G G A C G G C A A G

HP87

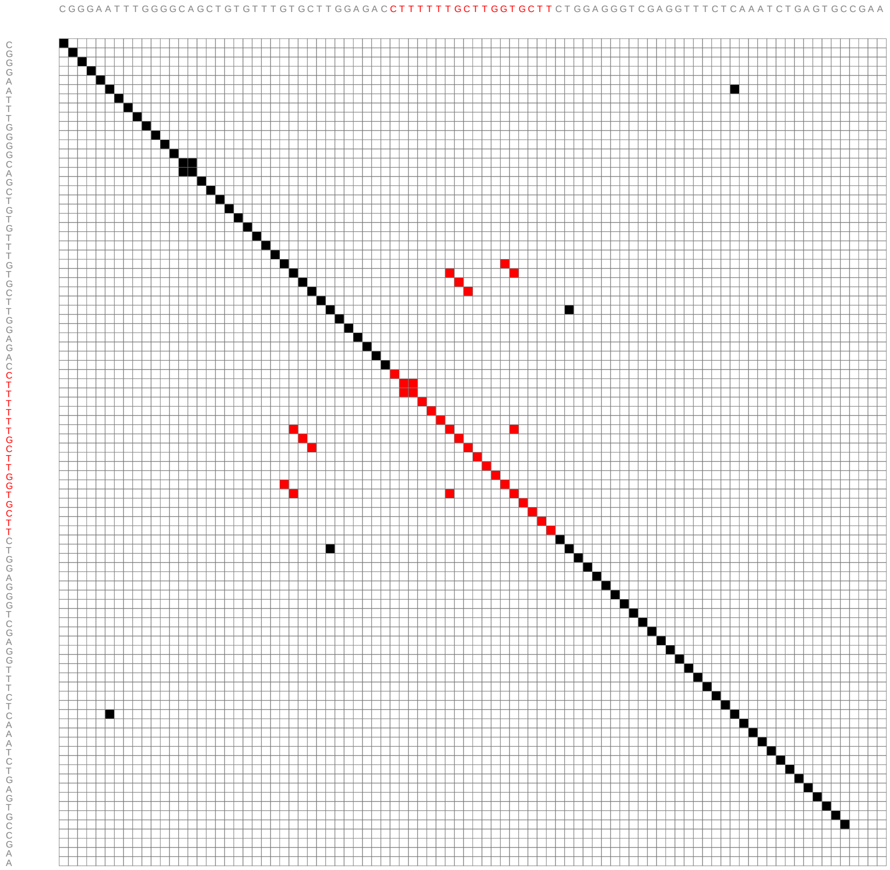

HP88

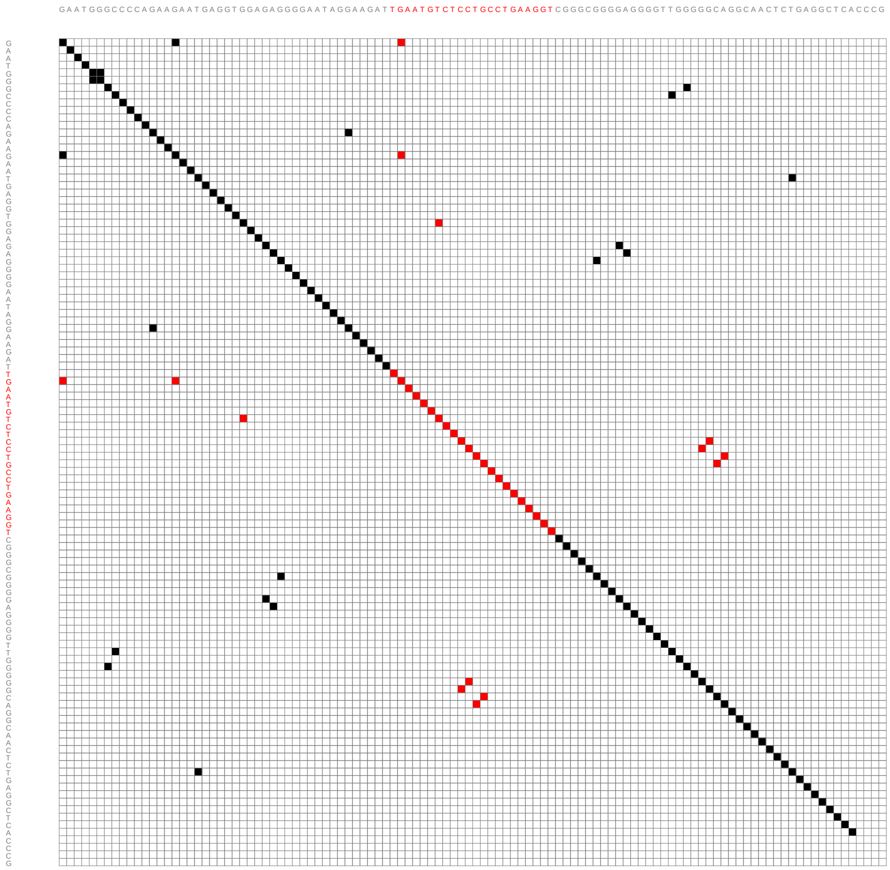

GAATGGGCCCCAGAAGAATGAGGTGGAGAGGGGAATAGGAAGATTGAATGTCTCCTGCCTGAAGGTCGGGCGGGGAGGGGTTGGGGGCAGGCAACTCTGAGGCTCACCCG
GAATGGGCCCCAGAAGAATGAGGTGGAGAGGGGAATAGGAAGATTGAATGTCTCCTGCCTGAAGGTCGGGCGGGGAGGGGTTGGGGGCAGGCAACTCTGAGGCTCACCCG
