# Supplementary material for: Diversification of CpG-Island Promoters Revealed by Comparative Analysis Between Human and Rhesus Monkey Genomes
Source: Mamm Genome. 2020 Jul 9;31(7):240–51. doi: 10.1007/s00335-020-09844-2 (PMC7496026; doi:10.1007/s00335-020-09844-2)
Supplement: Supplementary file 3 — Supplementary file3 (PDF 185 kb) [file 335_2020_9844_MOESM3_ESM.pdf]

Table S1. List of 88 indels in promoter alignments and ther repeat types

Protein-cording gene	Promoter flagment with an indel				
RefSeq ID	hg19	rhMac8	ID	Repeat	Repeat type
NM_053064	GGGCTGGGCTGGGCTGCGCCGGAGC	GGGCTGGGCT-----GCGCCGGAGC	HP01	Yes	Tandem/Inverted
NM_024326	GGGGTAAGAGGTTAGACGGGATGAACTAGG	GGGGTAAGAG----------ATGACCTAGG	HP02	No	-
NM_014869	GCTGAGCTGGGAGCTGGAGTGAGGGGC	GCTGAGCTGG-------AGTGAGGGGC	HP03	Yes	Tandem
NM_024712	GCGGGAGGGC--------AGGAGGGGCG	GCGGGAGGGCGGGTGGACAGGAGGGGCG	HP04	No	Tandem
NM_014815	CCCTTTGATACTTCTTTATTCATTTATT	CCCTTTGATA--------TTTATTTATT	HP05	Yes	Tandem
NM_177424	TCCCAGCTCCAGTCCCAGCTCCAGTCCCAGCG	TCCCAGCCCC------------AGTCCCAGCG	HP06	Yes	Tandem
NM_001039592,NM_172312	ATCGCGGTCGCGGTCGCGGTGAGAGC	ATCGCGGTCG------CGGTGAGAGC	HP07	Yes	Tandem
NM_014844	TCACGCTGCCGTGCGTTCACCAGGTT	TCAGGCTGCC------TCACCAGGTT	HP08	No	-
NM_030760	TGGGGGCGCT--------------------GGGCTGGGCC	TGGGGGCCCTTGGGGGCCCTTGGGGGCGCGGGGCTGGGCC	HP09	Yes	Tandem
NM_004864	TGTCCTCATATCGAGGAAGAGGATACTGAGGCCCA	TGTCCTCATA---------------CTGAGGCCCA	HP10	Yes	Tandem/Inverted
NM_005869	TGTAATACAAATAACATAAATAACATTAA	TGTAATACAA---------ATTACATTAA	HP11	Yes	Tandem
NM_024493	GTTTGCAGGAGTATGTGTGTGTGTGTGTGT	GTTTGCAGGG----------GTGTGTGTGT	HP12	Yes	Tandem
NM_000971	TCAGGGAGTT-----CTTTGCGTCT	TCAGGGAGTTGTTAATTTTGCGTCT	HP13	No	-
NM_173590	AGCAGGAAGT------------TCAGCAGGGG	AGCAGGAAGTGCAGCAGGAAGTTCAGCAGGGG	HP14	Yes	Tandem
NM_006912	AAGTTGTTAA-----TTTAAGTGTC	AAGTTGTTAAATGTTTTTAAGTGTC	HP15	Yes	Inverted
NM_003414	ATATACTTCTTCTTAAACTTAATTAGCTA	ATGTACTTCT---------TAATTAGCTA	HP16	Yes	Tandem/Inverted
NM_213607	AAGGGTTAGTTCTATGTATTAAATGCCTGG	AAGGGTTAGT----------AAATGCCTGG	HP17	No	-
NM_025061	AAAGGTGGCCGTAGAATGGCTTTGGGAGT	AAGGGTGGCC---------CTTTGGGAGT	HP18	No	-
NM_017578	CTGAGGCCAGGCGCCAGGTGTCGCCTGAAGCCAGACAGCCGGTT	CTGAGGCCAG------------------------ACAGCCGGTT	HP19	Yes	Tandem/Inverted
NM_001012337	CTGAGGCCAGGCGCCAGGTGTCGCCTGAAGCCAGACAGCCGGTT	CTGAGGCCAG------------------------ACAGCCGGTT	HP20	Yes	Tandem/Inverted
NM_018073	GCCAGGAGCT--------------GCTCCCTGAC	GCCAGGAGCTTACTGTCCCCAGCTGCTCCCTGAC	HP21	Yes	Inverted
NM_172059,NM_172060,NM_172058,NM_000503	TTTACATAAT-------TTTTTTTTTT	TTTACATAATAATTTTTTTTTTTTTTT	HP22	Yes	Tandem
NM_172059,NM_172060,NM_172058,NM_000503	AAAAAAGAAA------------------------------------GCCCCGAGGC	AAAAAAAAAAAAAAAAAAAGAAGAAGAAAAAGAAGAAAAAAAAAAAGCCC<br>TGAGGC	HP23	Yes	Tandem
NM_199235,NM_024027	GTTCCAGGCG---------TTCCCACACT	GTTCCTGGCGCTGCCGGCGTTCCCACACT	HP24	Yes	Inverted
NM_001012665,NM_015522,NM_016008	AATAAAAGAA------------------AATTGCTTTT	AATAAAAGAACATTTCCAGCCCAGAAACAATTGCTTTT	HP25	Yes	Inverted
NM_001012665,NM_015522,NM_016008	ACAGGAAAGAAAAGAAAGTTCCCTA	ACAGGAAAGA-----AAGTTCCCTA	HP26	Yes	Inverted
NM_001010938,NM_005781	GGGCCGGGCCGGGCCGGGCGCGGTG	GGGCCAGGCC-----GGGCGCGGTG	HP27	Yes	Tandem
NM_014364	CAGCAGGTCC----------AGGCCAATGA	CGGCAGGTCCCGGGGCAGCCAGGCCAATGA	HP28	Yes	Tandem
NM_007151,NM_001023560,NM_152736	AATGAGAAAACAAAAACAAAAACAGAAACAAAAAAA	AATGAGAAAG----------------AACTAAAAAA	HP29	Yes	Tandem
NM_017549	GAAGCGGCAGAAGGCAGTGGCAGCAGGCAGTGG	GAAGCGGCAG-------------CAGGCAGTGG	HP30	Yes	Tandem
NM_001003841	CAGGTGCCTG---------GGGCGAGGTG	CAGGTGCGTGCGGCGGGTGGGGCGAGGTG	HP31	Yes	Tandem
NM_016564	CCCACCGACCCCTCACCCACCGACCCGTCACCCACCGACCAAGGGGCAC<br>C	CCCACCGACC------------------------------AAGGGGCACC	HP32	Yes	Tandem
NM_016564	CCCCCTCCCC------------GCCCTCCTGC	CCCCCTCCCCCTCCCCCTCCCCGCCCTCCTGC	HP33	Yes	Tandem
NM_000334	CTCACTGAGGCGGTGGAGCTTGGAGC	CTCACTGAGG------AGCTTGGAGC	HP34	Yes	Tandem
NM_000334	AGCTTGGAGCCGGCCGCGCTCAGAGCGCG	AGCTTGGAGC---------TCAGAGGGCG	HP35	Yes	Inverted
NM_000334	AGGTGGAACAAAAGCTCTAGCTGGAGCCGGGGGCACGTG	AGGTGGAACC-------------------GGGGCACGCG	HP36	Yes	Tandem
NM_182833	TAAACTTAAACAGTCAAGTTCTTTTTG	TAAACTTACA-------GTTCTTTTTG	HP37	Yes	Inverted
NM_007155	CGCCCATCGC--------------CCCTAGAGCC	CGCCCACCGCTCACCGCCCAAAGCCCCTAGAACC	HP38	Yes	Tandem
NM_021992	AGGGGAAGGGCGGGGTGGGGGGGGGGGGGTGGCGAGCCCG	AGGGGAAGGG--------------------GGCGAGCCCG	HP39	Yes	Tandem
NM_024528	GAAATATATA----------------CTTCCCTTCA	GAAATATATATTCAGTGAAATATATACTTCCCTTCA	HP40	Yes	Tandem
NM_004270	TATGCCTTAATTCAAAAAGAAACGAAG	TATGCCTTAA-------AGAAACGAAG	HP41	Yes	Tandem
NM_023074	GGCCAGTGCC------GGGGTTTAAG	GGCCGGTGCCAATGCCGGGGTTTAAG	HP42	Yes	Tandem
NM_021216	CTCGGCTGGG-------CTCGGCTGAA	CTCTGCTGGGGGCAGGGCTCGGCTGAA	HP43	No	-
NM_003662,NM_001018109	CCCGCCTCCC-------GCCTCCTCTA	CCCGCCTCCCTCCTCCCGCCTCCTCTA	HP44	Yes	Tandem
NM_012286	CGGGCTAAAAAAAAAAACCTAAGAGG	CGGGCTAAAA------ACCTAAGAGG	HP45	Yes	Tandem
NM_004632,NM_033657	GGAGGCCGAG------AGCGATGAGA	GGAGGCCGAGGCCGAGAGCGACGAGA	HP46	Yes	Tandem
NM_004995	AACTTAGAGG-----TGTTTTTTTT	AACTTAGCGGGTTGGTTTTTTTTTT	HP47	No	-
NM_024900,NM_199320	CGGCGGCGGC------TGCAGGACGA	CGGCGGCGGCGGCGGCTGCAGGACGA	HP48	Yes	Tandem
NM_002514	CCTGTGCTGG---------GCGTGATCGG	CCTGTGCTGGTGGTGCTGGGCGTGATCCG	HP49	Yes	Tandem
NM_022496	GAAGCACAGC-------CCCTAACATT	GAAGCACAGCGGACACTCCCTAACAGT	HP50	No	-
NM_016216	AAAAAGAAAAAAGGAAAAAAAGAAAATAA	AAAAAGAAAA---------AAGAAAATAA	HP51	Yes	Tandem
NM_005284	AGGATGTGGGGAGAGGAGGGGAGAAAGATA	AGGATGTGGG----------GAGAAGGATA	HP52	Yes	Tandem
NM_003174,NM_021738	TCCAGGAAGTTTGGTTTTCTCACAGAGTGTAGCGTGCT	TCCGGGAAGT------------------GTAGCGTGCT	HP53	Yes	Inverted
NM_018378	GGGGAAGGAG------------CCTCCCCTCC	GGGGAAGGAGGCGGGGAAAGAGCCTCCCCTCC	HP54	Yes	Tandem
NM_015107	CGAAGAAGGT--------------GGGGGGTGGG	-GAAGAAGGTCGGGGGTGGGGGGTGGGGGGTGGG	HP55	Yes	Tandem
NM_183227,NM_016277	TAGACTCCTC-----CCTGCAGTGC	TAGACTCCTCTTCTCCCTGCAGTGC	HP56	Yes	Inverted
NM_032606	GAAGAGGGAG--------------GGGAGAGTGG	GAGGAGGGAGAGGATACTGAGGAGGGGAGAGTGG	HP57	Yes	Tandem
NM_005896	AGAAGCGGAGGCACTGGGAGTCCGGTTTGG	AGAAGCCGAG----------TCCGGTTTGG	HP58	Yes	Tandem
NM_017638	GGCTCTCTCTCTCTTTTTTTTTTTTTTTTTTTTTTTTTGA	GGCTCTCTCT------------------TTTTTTTTGA	HP59	Yes	Tandem
NM_152665	GTCTGCAGTG------CCGGAGGTCT	GTCTGCAGTGAACGAGCCGGAGGTCT	HP60	No	-
NM_152665	GCAGCGCGTCTGGACCCCAAAGGAAGGATAGGGG	GCAGCGCGTC--------------AGGACAGGGG	HP61	Yes	Tandem
NM_001042465,NM_001042466,NM_002778	TCGCTGGCTTTCTCTGGCTTTCTCTGGGCG	TCGCCGGCTT----------TCTCTGGGCG	HP62	Yes	Tandem
NM_005472	CGGTCAGAGGGCGCCGTGTCCAGCGAGCA	CGGTCAGAGG---------CCAGCGAGCA	HP63	Yes	Inverted
NM_000270	GGATCGGAGC--------------------ACACCGGAGC	GGATCGGAGCGGATCGGAGCACACCGGAGCACACCGGAGC	HP64	Yes	Tandem
NM_001256653	GGTCCTGGAG--------------------CTTTATCCAA	GGTCCTGGAGGGACCTGGTGGGTCCCGGAGCTTTATCCAA	HP65	Yes	Tandem/Inverted
NM_000384	CTGGCGCTGCCTGCGCTGCTGCTGCTGCTGCT	CCGGCGCTGC------------TGCTGCTGCT	HP66	Yes	Tandem
NM_182496	CGCGCGGGTG----------------GCGGGTGCCC	CGCGCGGGTGCCGCCACCCTGAGGTGGCGGGTGCCG	HP67	Yes	Inverted
NM_182496	ATCCAAACTC------GCTGAGGTAA	ATCCAAACTCAAACTCGTTGAGGTAA	HP68	Yes	Tandem
NM_022893,NM_018014,NM_138559,NM_138553	CGCCGCCGCC-------CGCCCCGCAG	CGCCGCCGCCGCCCGCCCGCCCCGCAG	HP69	Yes	Tandem
NM_000947	GGCGGGTTGG--------------------TTGTTTGGGG	GGCGGGTTGAGAGTAGTGGGTATTGGTTGATTGGTTGGGG	HP70	Yes	Tandem
NM_032828	TTGTGGTGCCTTCCATGCCCTCAGGTCAC	TTGTGGCGCC---------CTCAGGTCAT	HP71	Yes	Tandem
NM_003193	GCCTGCTCCG---TGCGCTGGGA	GCCTGTTCCGAGCGGCTTTGAACCAGCCCAGAAGTGCGGGCCCGAAGG<br>GAATGCGTGTGTGCTGGGA	HP72	Yes	Tandem/Inverted
NM_002797	GCAGGTACGTCCTTTTAGAATTTAAGGTTAG	GCAGGTACGT-----------TTAAGATTAG	HP73	No	-
NM_080737	ACATCGCGGC------TTCTCTGGCG	ACATCTCGGCGTCGGCTTCTCTGGCG	HP74	Yes	Inverted
NM_022821	GGCACGCCCC------TGCCCCCGCC	GGCCCGCCCCAGCCTCTGCCCCCGCC	HP75	Yes	Tandem
NM_022821	CCAGCCCGGCCCAGCCCGGCCCAGCCCTGC	CCAGCCCGGC----------CCAGCTCTGC	HP76	Yes	Tandem
NM_000107	AAAAAAAAAAAAAAATCCATAAAGCCG	AAAAATAAAA------CATAAAGCCG	HP77	Yes	Tandem
NM_000107	GCACCGCCCCTTGGCACCACCCCCTCCCCGCGC	GCACCGCCCC-------------CTCCCCGCGC	HP78	Yes	Tandem
NM_006472	CCTCTCCTTCCTCTCCTTCCCCCCTCCCT	CCTCTCCTTC---------CCCCCTCCCT	HP79	Yes	Tandem
NM_016293	TGGCTCAGGGCTGGGGCCGGATGCGA	TGGCTCAGGG------CCGGATGCGA	HP80	No	-
NM_016293	GCCCACAGGGAGCCGAGGCCGAGGCCG	GCCCACAGGA-------GCCGAGGCCG	HP81	Yes	Tandem
NM_031486,NM_001007101	GTAAGAAGTACCTCTAGGCCTGGGAGGCATT	GTAAGAAGTA-----------GGGAGGCATT	HP82	Yes	Inverted
NM_173797	CCAAGTACGC---------CCAGCTCGGG	CCGAGTACGCTCAGCCGTCCCAGCTCGGG	HP83	Yes	Inverted
NM_005655,NM_001032282	GTAAAGAATG-------------------------TTTTTTGGTA	GTAAAGAATGGTTTGTTTGGTTTTTTTTTTTTTTTTTTTTTGGTA	HP84	Yes	Tandem
NM_005655,NM_001032282	CTGGACAGTT-------TTCTTTTAAT	CTGAACAGTTGTTTTTTTTTTTTTAAT	HP85	Yes	Tandem
NM_016593	AAGAAGCAAG-----GGACGGCAGG	AAGAAGCAAGTGCCAGGACGGCAAG	HP86	Yes	Inverted
NM_003576,NM_001032296	GCTTGGAGACCTTTTTTGCTTGGTGCTTCTGGAGGGTC	GCTTGGAGGC------------------CTGGAGGGTC	HP87	Yes	Tandem
NM_001252	ATAGGAAGATTGAATGTCTCCTGCCTGAAGGTCGGGCGGGGA	ATAGGAAGAT----------------------CGGGCGGGGA	HP88	Yes	Inverted

Table S2. Alterations of CpG sites in promoters

CpG sites on human promoter

	AA	AT	AC	AG	TA	TT	TC	TG	GA	GT	GC	GG	CA	CT	CC	CG	Deletion
hCmC	84	20	42	3126	178	91	119	6700	123	56	113	3425	6372	3302	3381	183120	3096
hCmN	3	1	1	83	9	4	1	264	1	0	1	81	235	90	88	3104	56
hNmC	0	0	1	26	1	4	2	64	1	0	1	34	56	29	41	1961	35
hNmN	26	2	8	701	137	34	36	4302	35	18	14	890	4268	708	821	28758	618
All promoter	113	23	52	3936	325	133	158	11330	160	74	129	4430	10931	4129	4331	216943	3805
rheMac8.hg38	30154	7471	8307	450395	104326	30802	25703	2430106	25559	8252	7834	388303	2435201	451675	387440	6055963	210511
rheMac3.hg19	28378	6921	7519	427603	99112	28896	23468	2329630	23671	7476	6271	359599	2333117	427900	359763	5393577	196609

CpG sites on mocaque promoter

	AA	AT	AC	AG	TA	TT	TC	TG	GA	GT	GC	GG	CA	CT	CC	CG	Deletion
hCmC_90.list	76	14	39	2796	132	83	119	6080	100	49	110	3515	5706	2944	3462	183120	2546
hCmN_90.list	0	0	0	38	2	0	0	91	0	1	1	52	109	38	56	3104	33
hNmC_90.list	2	1	1	49	3	1	4	127	2	1	1	72	138	41	62	1961	29
hNmN_90.list	37	7	13	789	114	36	34	4384	43	11	11	885	4199	810	927	28758	495
All promoter	115	22	53	3672	251	120	157	10682	145	62	123	4524	10152	3833	4507	216943	3103
rheMac8.hg38	24689	5689	6792	348489	105158	24704	24004	2309205	24174	6775	7726	361893	2312550	348115	362600	6055963	239848
rheMac3.hg19	22861	5175	6193	327098	99049	22762	21998	2205678	22232	6232	6285	335194	2209134	326763	334749	5393577	208860

Table S3. List of 439 indels in promoter alignments and ther repeat types

Promoter classification		TSS position		Promoter flagment with an indel		Protain-cording Gene				No. of CpG		ΔCpG
hg19	rhMac8	hg19	rhMac8	hg19	rhMac8	RefSeq ID	Alignment score	Indel length	Indel type	hg19	rhMac8	(hCpG - mCpG)
CpG	Non-CpG	chr17:67411138	chr16:62823302	CAGAGGCTTTTCGAGAACAAA	CAGAGGCTTT-CGAGAATAAA	NM_002758	98	1	Type-IIa	0	0	0
CpG	Non-CpG	chr17:67411138	chr16:62823302	ACTGAGTGTGTGTCTGACTTGT	ACTGAGTGTG--TCTGACTTGT	NM_002758	98	2	Type-I	0	0	0
CpG	Non-CpG	chr17:67411138	chr16:62823302	ACTCCGCCGGAGAACTACATT	ACTCCGCTGG-GAACTACATT	NM_002758	98	1	Type-I	0	0	0
CpG	Non-CpG	chr11:8893214	chr14:57607155	CCCCGAGGCCCGAGAACTGGG	CCCCGAGGCT-GAGAACTGGG	NM_139157, NM_005418, NM_213618	97	1	Type-IIa	1	0	1
CpG	Non-CpG	chr11:8893214	chr14:57607155	CCCTCACCCC-TGTCCCCCAC	CCCTCACCCCCTGTCCCCCAC	NM_139157, NM_005418, NM_213618	97	1	Type-IIa	0	0	0
CpG	Non-CpG	chr17:46622383	chr16:41342721	TCGGGGGGGGTGCAGGAGGGG	TCTGGGGGGG-GCAGGAGGGG	NM_002145	97	1	Type-IIa	0	0	0
CpG	Non-CpG	chr17:46622383	chr16:41342721	AGGAGGGGGG-TCCCTTCCGA	AGGAGGGGGGGTCCCTTCCGA	NM_002145	97	1	Type-IIa	0	0	0
CpG	Non-CpG	chr17:46622383	chr16:41342721	ACGCCCCCCCCAGCAGCCCCC	ACGCCCCCCC-AGCAGCCCCC	NM_002145	97	1	Type-IIa	0	0	0
CpG	Non-CpG	chr17:46622383	chr16:41342721	GAGGGGCAGC-TAGAAGAGGG	GAGGGGCACCCTACAAGAGGG	NM_002145	97	1	Type-IIa	0	0	0
CpG	Non-CpG	chrX:71792993	chrX:67341752	TGCCATAAAAACAATCGATCC	TGCCATAAAA-CAATCGATCC	NM_018486	97	1	Type-IIa	0	0	0
CpG	Non-CpG	chr10:61122888	chr9:73391181	ACAAAAAACA---CACACACAAA	ACAAACAACAACACACACACAAA	NM_001001971, NM_198215	96	3	Type-IIb	0	0	0
CpG	Non-CpG	chr10:61122888	chr9:73391181	AACGATGGATGAATCTCTGCGAGC	AACGATGGAT----CTCTGCGAGC	NM_001001971, NM_198215	96	4	Type-IV	0	0	0
CpG	Non-CpG	chr10:61122888	chr9:73391181	GCCTCGGGGGGTGGCTGGCAC	GCCTCGGGGG-TGGCTGGCAC	NM_001001971, NM_198215	96	1	Type-IIa	0	0	0
CpG	Non-CpG	chr11:134095648	chr14:127179680	CCTCACCCAGAAATCCTGGGGA	CCTCACTCAG--ATCCTGGGGA	NM_015261	96	2	Type-IIa	0	0	0
CpG	Non-CpG	chr11:134095648	chr14:127179680	TTTTGGCAGGGAGGACAGTAA	CTTTGGCAGG-AGGACAGTAA	NM_015261	96	1	Type-IIa	0	0	0
CpG	Non-CpG	chr11:134095648	chr14:127179680	CTCGCCGGAGGAGCTGGGCCCTG	CTCGCCGGAG---CTGGGCCCTG	NM_015261	96	3	Type-IIb	0	0	0
CpG	Non-CpG	chr12:56368185	chr11:54701366	CTGGAGCCCC-GCCCCAGGGC	CTGGAGCCCCCGCCCCAGGGC	NM_002868	96	1	Type-IIa	1	1	0
CpG	Non-CpG	chr12:56368185	chr11:54701366	CTAATGGGGGTCGCTGAGGAG	CTAATGGGGG-CGCTGAGGAG	NM_002868	96	1	Type-IV	0	0	0
CpG	Non-CpG	chr14:51027163	chr7:112569157	CACGGGGTCTTCTGGCCCTCCAC	CACGGGGTCT---GGCCCTCCGC	NM_181598, NM_015915	96	3	Type-IIb	0	0	0
CpG	Non-CpG	chr14:51027163	chr7:112569157	TTCTGCTTCTGTGGAGACAGGG	TTCTGCTTCT--GGAGACAGGG	NM_181598, NM_015915	96	2	Type-I	0	0	0
CpG	Non-CpG	chr14:52327630	chr7:113827105	GGGCTGGGCTGGGCTGCGCCGGAGC	GGGCTGGGCT-----GCGCCGGAGC	NM_053064	96	5	Type-IIc	0	0	0
CpG	Non-CpG	chr14:52327630	chr7:113827105	GCTCCGGAGA--GGGGAAAACC	GCTCCGGAGACAGGGGAAAACC	NM_053064	96	2	Type-IV	0	0	0
CpG	Non-CpG	chr1:177140569	chr1:190389699	GGGACAGGGG-ACTCCCCCAG	GGGACAGGGGGACTCCCCCAG	NM_021165	96	1	Type-IIa	0	0	0
CpG	Non-CpG	chr3:159558144	chr2:134913807	CGGATATTCA-GGGATTTTGC	CGGATATTCAAGGGATTTTGC	NM_014575	96	1	Type-IIa	0	0	0
CpG	Non-CpG	chr5:95769252	chr6:93399669	TCAAGTGGGG-CACTTCTCTT	TCAAATGGGGGCACTTCTCTT	NM_000439	96	1	Type-IIa	0	0	0
CpG	Non-CpG	chr5:95769252	chr6:93399669	TTTTTCCTCTTCCCTTCCCAC	TTTTTCCTCT-CCCTTCCCAC	NM_000439	96	1	Type-IIa	0	0	0
CpG	Non-CpG	chr5:146258648	chr6:144943057	GAGGGGAGGA-GGTTGGAAGA	GAGGGGAGGAAGGTTGGAAGA	NM_181676, NM_181678, NM_181677, NM_181674, NM_181675, NM_004576	96	1	Type-IIa	0	0	0
CpG	Non-CpG	chr10:104179246	chr9:98135420	CCACCCTCCC----AGGATGCAGC	CCACCCCCCCCCCAGGATGCAGC	NM_024326	95	4	Type-IIa	0	0	0
CpG	Non-CpG	chr10:104179246	chr9:98135420	GGGGTAAGAGGTTAGACGGGATGAACTAGG	GGGGTAAGAG----------ATGACCTAGG	NM_024326	95	10	Type-IV	1	0	1
CpG	Non-CpG	chr11:118955892	chr14:111941435	CCGTGGTCATGCCGCTGCACT	CCGTGGTCAT-CCACTGCACT	NM_000190, NM_001024382	95	1	Type-IV	0	0	0
CpG	Non-CpG	chr11:118955892	chr14:111941435	GAGGTCCACT--GTCGCAATGT	GAGGTCCTCTCTGTCGCAATGT	NM_000190, NM_001024382	95	2	Type-I	0	0	0
CpG	Non-CpG	chr11:118955892	chr14:111941435	TCCTCAGTCGTCTATTGGTCA	TCCTCAGTCG-CTATTGGTCA	NM_000190, NM_001024382	95	1	Type-III	0	0	0
CpG	Non-CpG	chr12:25055456	chr11:25831478	CCTCCTTTTTTGTGTTTGCCT	CCTGCTTTTT-GTGTTTGCCT	NM_005504	95	1	Type-IIa	0	0	0
CpG	Non-CpG	chr16:29828516	chr20:28212526	ATAGGGAGGGGAAAATGTGGG	ATAAGGAGGG-AAAATGTGGG	NM_024516	95	1	Type-IIa	0	0	0
CpG	Non-CpG	chr16:29828516	chr20:28212526	GAGGAGGAAC-GGGCAGGGGA	GAGGAGGAACCGGGCAGGGGA	NM_024516	95	1	Type-IIa	1	1	0
CpG	Non-CpG	chr17:7348713	chr16:7422127	CTCCACTCCA-GCTTTCTCAA	CTCCACTCCACGCTTTCTCAA	NM_000747	95	1	Type-IV	0	1	-1
CpG	Non-CpG	chr17:7348713	chr16:7422127	CTGGCGGCTGTCCTCCAGCAA	CTGGCGGCTG-CCCCAGCAA	NM_000747	95	1	Type-III	0	0	0
CpG	Non-CpG	chr19:36239798	chr19:31663533	GTCCCCGCCT-TCTCCCGCCT	GTCCCCGCCCGCCTCCCGCCT	NM_019104	95	1	Type-IV	0	1	-1
CpG	Non-CpG	chr19:49631884	chr19:44526990	GCATGAGGGGGCGGGGCCTCG	GCGTGAGGGG-CGGGGCCTCG	NM_003660	95	1	Type-IIa	0	0	0
CpG	Non-CpG	chr19:49631884	chr19:44526990	GGCCTCGGGGGGAAGAAGGCGG	GGCCTCGGGG--AAGAAGGCTG	NM_003660	95	2	Type-IIa	0	0	0
CpG	Non-CpG	chr19:49631884	chr19:44526990	CTAAGTGGGGGCGGGGCCTCG	CTAAGTGGGG-CGGGGCCTCG	NM_003660	95	1	Type-IIa	0	0	0
CpG	Non-CpG	chr19:49631884	chr19:44526990	CTATTTTTTTCCCCCATCTCGCC	CTATTTTTTT---CCATCTCGCC	NM_003660	95	3	Type-IIa	0	0	0
CpG	Non-CpG	chr19:49631884	chr19:44526990	CTGTCTTCTCCTCTTCCCTGCTC	CTGCCTTCTC---TTCCCTGCTC	NM_003660	95	3	Type-IIb	0	0	0
CpG	Non-CpG	chr19:54928231	chr19:49717625	GCTCCTCGCC-GCCCCCTCTC	GCTCCTCGCCCGCCCCCTCTC	NM_020659, NM_001005367	95	1	Type-IIa	1	1	0
CpG	Non-CpG	chr1:29213915	chr1:27860979	GAACGCGGTC-GGCCCGGTCC	GAACGCGGTCCGGCCCAGTCC	NM_004437, NM_203342, NM_203343	95	1	Type-IIa	1	1	0
CpG	Non-CpG	chr1:2005373	chr1:1109133	TATTGTCGGGGCCGGAGGCGA	TATTGTCGGG-CCGGAGGCGA	NM_001033582, NM_001033581, NM_002744	95	1	Type-IIa	0	0	0
CpG	Non-CpG	chr5:33892395	chr6:34580997	GCACCGAGCGGGCGGGCTGCGAGG	GCACCAAGCG----GGCTGCGAGG	NM_030955	95	4	Type-IIb	1	0	1
CpG	Non-CpG	chr5:33892395	chr6:34580997	CTCGCGCCTG-ACCATGCACC	CTCGCGCCCGGACCATGCACG	NM_030955	95	1	Type-IIa	0	0	0
CpG	Non-CpG	chr6:37225848	chr4:38205786	AATCCCCTCT--TCCTTCCCAT	AATCCCCTCTCTTCCTTCCCAT	NM_017772	95	2	Type-I	0	0	0
CpG	Non-CpG	chr6:37225848	chr4:38205786	TCTCGCGGGT-AGGAACTTTG	TCTTGCGGGTTAGGAACTTTG	NM_017772	95	1	Type-IIa	0	0	0
CpG	Non-CpG	chr11:101454989	chr14:94494260	CGGAGAGTGC-GGGGGATGAA	CGGAGAATGCCGGGGGATGAA	NM_004621	94	1	Type-IIa	1	1	0
CpG	Non-CpG	chr12:57024410	chr11:55421170	CCCCCTCCCC-AGCGGGCGTC	CCCCCTCCCCCAGCGGGCGTC	NM_013449	94	1	Type-IIa	0	0	0
CpG	Non-CpG	chr14:24804577	chr7:85747428	CACCCCAGCCCACCCCTTCGC	CACCCCAGCC-ACCCCTTCGC	NM_139247	94	1	Type-IIa	0	0	0
CpG	Non-CpG	chr14:24804577	chr7:85747428	TGGGAGGGGG-CCGCGGCGCC	TGGGAGGGGGGCCGCGGCGCC	NM_139247	94	1	Type-IIa	0	0	0
CpG	Non-CpG	chr14:75180173	chr7:137099251	GGGTCTGGGTTATTTGAGGCA	GGGTCTGGGT-ATTTGAGGCA	NM_015962	94	1	Type-IIa	0	0	0
CpG	Non-CpG	chr17:39942647	chr16:47615870	GCAAGGCGGGGAGGGATCCGG	GCAGGGCGGG-AGGGATCCGG	NM_002230, NM_021991	94	1	Type-IIa	0	0	0
CpG	Non-CpG	chr17:39942647	chr16:47615870	GCTGGTCTCTTTGGGGCCACCC	GCTGGTCTCT--GGGGCCACTC	NM_002230, NM_021991	94	2	Type-IIa	0	0	0
CpG	Non-CpG	chr17:39942647	chr16:47615870	ATGGAGTGAC--TGGGGGGTGC	ATGGAGTGACAGTGGTGGGTGC	NM_002230, NM_021991	94	2	Type-IV	0	0	0
CpG	Non-CpG	chr17:39942647	chr16:47615870	CAGCTGGTGT--CTGGCACCCG	CAGCTGGTGTGTCTGGCATCCG	NM_002230, NM_021991	94	2	Type-I	0	0	0
CpG	Non-CpG	chr18:52495730	chr18:44266025	TGTTGCATACTGATTAATGCA	TGTTGCATAC-AATTAATGCA	NM_004163	94	1	Type-IV	0	0	0
CpG	Non-CpG	chr18:52495730	chr18:44266025	CCCCAGTTCT---CACAGCGCCC	CCCCAGTTCTTCTCACAGCGCCC	NM_004163	94	3	Type-IIb	0	0	0
CpG	Non-CpG	chr18:52495730	chr18:44266025	GAAAACACAG---AACAAAAATG	GAAAACACAGGAAAAAAAAAAATG	NM_004163	94	3	Type-IIa	0	0	0
CpG	Non-CpG	chr19:49496866	chr19:44397594	CGGAGCCCCCCTTCCTGCGCC	CCGAGCCCCC-TTCCTGCGCC	NM_002103	94	1	Type-IIa	0	0	0
CpG	Non-CpG	chr22:30793307	chr10:72235677	ACGGGGCTGGGTGGGGGCCGA	ACGGGGCTGG-TGGGGGCCGA	NM_012429	94	1	Type-IIa	0	0	0
CpG	Non-CpG	chrX:16731092	chrUn_NW_014851788v1:1273	CAGCAGGGAGGAGAGGGGAAGGT	CAGCAGGGAG---AGGGGAGGGT	NM_175859, NM_019857	94	3	Type-IIb	0	0	0
CpG	Non-CpG	chrX:16731092	chrUn_NW_014851788v1:1273	TTGGACAGCC----CCGGCAGCGA	TTGGACAGCCAGCCCCGGCAGCGA	NM_175859, NM_019857	94	4	Type-IIc	0	0	0
CpG	Non-CpG	chr2:230933940	chr12:112516672	CATTTTTTTT-CCCTGCCAAA	CATTTTTTTTTCCCTGCCAAA	NM_152527	94	1	Type-IIa	0	0	0
CpG	Non-CpG	chr3:12329165	chr2:54123237	ACTCCGTCCCCACCCATGTTG	ACTCGGTCCC-ACCCATGTTT	NM_138712, NM_015869, NM_138711, NM_005037	94	1	Type-IIa	0	0	0
CpG	Non-CpG	chr3:12329165	chr2:54123237	CACACCTCGG-TCTCCCCAGA	CACACCTAGGCTCTCCCCAAA	NM_138712, NM_015869, NM_138711, NM_005037	94	1	Type-I	0	0	0
CpG	Non-CpG	chr3:13114917	chr2:53331740	GGAGGGAAAA-ACCCCAAGGT	GGAGGGGAAACACGCCAAGGT	NM_014869	94	1	Type-IIa	0	0	0
CpG	Non-CpG	chr3:13114917	chr2:53331740	GCTGAGCTGGGAGCTGGAGTGAGGGGC	GCTGAGCTGG-------AGTGAGGGGC	NM_014869	94	7	Type-IIc	0	0	0
CpG	Non-CpG	chr1:40724208	chr1:39569322	GGGGCTTTTCCTGTAGAAACT	GGGGCTTTTC-TGTAGAAACT	NM_005857	94	1	Type-IIa	0	0	0
CpG	Non-CpG	chr8:67974668	chr8:65203340	GTCCCTGTCT--TCCCTGAATG	GTCCCTGTCTCTTCCCTGAATG	NM_006837	94	2	Type-I	0	0	0
CpG	Non-CpG	chr8:86351387	chr8:83834425	CGGTTAGAAA-CCCTGCAGTT	CAGTTAGAAAGCCCTGCAGTT	NM_005181	94	1	Type-IV	0	0	0
CpG	Non-CpG	chr8:86351387	chr8:83834425	AGTGTGCGGG--GGAGCTACAT	AGTGTGCGGGAGGGAGCTACAT	NM_005181	94	2	Type-IIb	0	0	0
CpG	Non-CpG	chr8:86351387	chr8:83834425	CTTCTTTTTTTCCTTCATCTC	CTTCTTTTTC-CCTTCATCTC	NM_005181	94	1	Type-IIa	0	0	0
CpG	Non-CpG	chr1:156711180	chr1:131370281	TTCCTACCCCCGCCAAACTTC	TTCCTACCCC-GCCAAACTTC	NM_024540, NM_145729	93	1	Type-IIa	1	1	0
CpG	Non-CpG	chr16:67233922	chr20:51476105	GCGGGAGGGC--------AGGAGGGGCG	GCGGGAGGGCGGGTGGACAGGAGGGGCG	NM_024712	93	8	Type-IIa	0	1	-1
CpG	Non-CpG	chr17:38210961	chr16:45855167	CCCTTTGATACTTCTTTATTCATTTATT	CCCTTTGATA--------TTTATTTATT	NM_014815	93	8	Type-IIb	0	0	0
CpG	Non-CpG	chr19:12405951	chr19:12348961	ACTACGGGAG---TTGGGAGGAC	GCTACGGGAGGAGTTGGGAGGAC	NM_016264	93	3	Type-IIb	0	0	0
CpG	Non-CpG	chr19:12405951	chr19:12348961	GGACCTGAGCGCCCCCTGCGC	GGACCCGAGC-CCCCCTGTGC	NM_016264	93	1	Type-I	1	0	1
CpG	Non-CpG	chr19:49127929	chr19:43991330	CCCTCCCCCC-ACCCCTAGGT	CCCTCCCCCCCACCCCTAGGT	NM_020126	93	1	Type-IIa	0	0	0
CpG	Non-CpG	chr19:49127929	chr19:43991330	CTGAACCCAGCCAGGCCTGGGACT	CTGAACCCAG----GCCTGGGACT	NM_020126	93	4	Type-IIc	0	0	0
CpG	Non-CpG	chr19:49127929	chr19:43991330	TGGAAATAAGAGTGCGGATGG	TGGAAATAGG-GTGCGGATGG	NM_020126	93	1	Type-IIa	0	0	0
CpG	Non-CpG	chr20:1306720	chr10:34095820	TGAGAAACGG-GGGAGGAAGG	TGAGAAAGGGTGGGAGGAAGG	NM_015685, NM_080489	93	1	Type-IIa	0	0	0
CpG	Non-CpG	chr20:1306720	chr10:34095820	GGCTGCCTTA-GTACAGAATC	GGCTGCCTTAAGTACAGAATC	NM_015685, NM_080489	93	1	Type-IIa	0	0	0
CpG	Non-CpG	chr20:33451721	chr10:29269128	AGACCGACCCCGGCTGCCTGC	AGACCGACCC-GGCTGCCTGC	NM_178026	93	1	Type-IIa	1	1	0
CpG	Non-CpG	chr2:38303079	chr13:38535861	GAACCCGCAG-CCCGGCCTGG	GAACCCGCAGGCCCGCCCTGG	NM_173652	93	1	Type-IIa	0	0	0
CpG	Non-CpG	chr1:28100162	chr1:26654474	TCCCAGCTCCAGTCCCAGCTCCAGTCCCAGCG	TCCCAGCCCC------------AGTCCCAGCG	NM_177424	93	12	Type-IIc	0	0	0
CpG	Non-CpG	chr4:74486640	chr5:61075891	CCTAAGAAAG----TAAACACTCC	CCTAAGAAAGAAAGTAAACACTCC	NM_177532, NM_201431	93	4	Type-IIb	0	0	0
CpG	Non-CpG	chr4:74486640	chr5:61075891	CAGGTTTCTTGACACTTCCCT	CAGGTTTCTT-ACACTTCCCT	NM_177532, NM_201431	93	1	Type-IV	0	0	0
CpG	Non-CpG	chr6:39282527	chr4:40256510	TGCCCACAGT-TCAGAGCCTC	TGCCCACAGTCTCAGAGCCTC	NM_031460	93	1	Type-I	0	0	0
CpG	Non-CpG	chr9:35812467	chr15:48448012	ATCGCGGTCGCGGTCGCGGTGAGAGC	ATCGCGGTCG------CGGTGAGAGC	NM_001039592, NM_172312	93	6	Type-IIc	2	0	2
CpG	Non-CpG	chr10:17686449	chr9:17775387	GGGTGAGAGG-AGGAGCTGTC	GGGTGAGAGGGAGGAGCTGTC	NM_003473	92	1	Type-IIa	0	0	0
CpG	Non-CpG	chr10:17686449	chr9:17775387	GTCGAGAGGG-AGTCCCCGGG	GTCGAGGGGGGAGTCCCCGCG	NM_003473	92	1	Type-IIa	0	0	0
CpG	Non-CpG	chr1:118728101	chr1:119365606	CCCGACCTGC-GAGGCCAGCC	CCCGACCTGCCGAGGCCAGCC	NM_206996	92	1	Type-IIa	1	1	0
CpG	Non-CpG	chr1:151227521	chr1:125753240	AGATCCTGTTTTTTGTGTTACCGC	AGATCCTGTT----GTGTTACCGC	NM_002810, NM_153822	92	4	Type-IIa	0	0	0
CpG	Non-CpG	chr13:25338609	chr17:4495839	TCCTGGTGTCACCAGAAACTG	TCCTGGTGTC-CCGGAAACTG	NM_031277	92	1	Type-IIa	0	0	0
CpG	Non-CpG	chr14:102904806	chr7:164684565	GCCCCCGGGGTGCAGACATCT	GCCCCCGGGG-GCAGACATCT	NM_014844	92	1	Type-IIa	0	0	0
CpG	Non-CpG	chr14:102904806	chr7:164684565	TCACGCTGCCGTGCGTTCACCAGGTT	TCAGGCTGCC------TCACCAGGTT	NM_014844	92	6	Type-IIc	2	0	2
CpG	Non-CpG	chr14:102904806	chr7:164684565	GTTTGAGGGG-TTGTCTCCAA	GTTTAAGGGGGTTTTCTCCAA	NM_014844	92	1	Type-IIa	0	0	0
CpG	Non-CpG	chr14:102904806	chr7:164684565	GTGTAAATGGGTGTTCAGTTT	GTGTAAATGG-TGTTCAGTTT	NM_014844	92	1	Type-IIa	0	0	0
CpG	Non-CpG	chr17:18759981	chr16:18739208	ATAATCACTGGGCTCATGGGTCTT	ATAATCACTG----CATGGGCCTT	NM_002767	92	4	Type-IIc	0	0	0
CpG	Non-CpG	chr17:18759981	chr16:18739208	TCTGATGAAT-ATTTCATGCC	TCTGATGAATGATTTCATGCC	NM_002767	92	1	Type-III	0	0	0
CpG	Non-CpG	chr19:10628423	chr19:10552169	TGGGGGCGCT--------------------GGGCTGGGCC	TGGGGGCCCTTGGGGGCCCTTGGGGGCGCGGGGCTGGGCC	NM_030760	92	20	Type-IIc	0	2	-2
CpG	Non-CpG	chr19:18497268	chr19:18418874	TGTCTTTTTTTCCTCCAAAAA	TGTCTTTTTT-CCTCCAAACA	NM_004864	92	1	Type-IIa	0	0	0
CpG	Non-CpG	chr19:18497268	chr19:18418874	TGTCCTCATATCGAGGAAGAGGATACTGAGGCCCA	TGTCCTCATA---------------CTGAGGCCCA	NM_004864	92	15	Type-IV	1	0	1
CpG	Non-CpG	chr20:271379	chr10:33026299	AAAAAAAAAAAATTGGAAGAAA	AAAAAAAAAA-TTGGAAGAAA	NM_153269	92	1	Type-IIa	0	0	0
CpG	Non-CpG	chr20:271379	chr10:33026299	CTCGAGGTCG-CCCTCGGTCT	CTAGAGGTCGGCCCTCAGTCT	NM_153269	92	1	Type-IIa	0	0	0
CpG	Non-CpG	chr20:271379	chr10:33026299	TTCTGACCCCCGTCCTCTCCC	TTCTGACCCC-GCCCTCTCCC	NM_153269	92	1	Type-IIa	1	1	0
CpG	Non-CpG	chr1:24018596	chr1:22487956	GTGCTGATTC-GGGAGCTTTC	GTGCTGATTCTAGGAGCTTTC	NM_000975	92	1	Type-IIa	1	0	1
CpG	Non-CpG	chr3:50607253	chr2:91744818	ACACATCCCC-GCACTTTCCT	ACACATCCCCCGCACTTTCCT	NM_016173	92	1	Type-IIa	1	1	0
CpG	Non-CpG	chr3:50607253	chr2:91744818	GTGGGTGGGGCAGCCTTTCAG	GAGGGTGGGG-AGCCTCTCAG	NM_016173	92	1	Type-IV	0	0	0
CpG	Non-CpG	chr3:50607253	chr2:91744818	CACTGGTGCA-AGAGCCGCTT	TACTGGTGCACAGAGCCGCTT	NM_016173	92	1	Type-I	0	0	0
CpG	Non-CpG	chr4:152682437	chr5:151674182	CACTCTAACT--TATACACACG	CACTCAAACTCATATATACACG	NM_004564	92	2	Type-I	0	0	0
CpG	Non-CpG	chr4:152682437	chr5:151674182	TGTTTCATAC-ATTTCTGCCC	TGTTTCATACCATTTCTGCCC	NM_004564	92	1	Type-IIa	0	0	0
CpG	Non-CpG	chr4:152682437	chr5:151674182	CCACCTAACTGTGAGGTGGAAC	CCACCTAACT--GAGGTGGAAC	NM_004564	92	2	Type-I	0	0	0
CpG	Non-CpG	chr5:64065076	chr6:62931921	TGTAATACAAATAACATAAATAACATTAA	TGTAATACAA---------ATTACATTAA	NM_005869	92	9	Type-IIb	0	0	0
CpG	Non-CpG	chr6:28318001	chr4:28771277	AAATAAAAAT-TTAAAAGCCT	AAATAAAAAAATCAAAAGCCT	NM_024493	92	1	Type-IIa	0	0	0
CpG	Non-CpG	chr6:28318001	chr4:28771277	GTTTGCAGGAGTATGTGTGTGTGTGTGT	GTTTGCAGGG----------GTGTGTGTGT	NM_024493	92	10	Type-I	0	0	0
CpG	Non-CpG	chr7:56120408	chr3:55536549	CTACACCTGT-CCTCCATCCT	CTACACCTGTTCCTCCAGCCT	NM_001009186, NM_001762	92	1	Type-IIa	0	0	0
CpG	Non-CpG	chr7:56120408	chr3:55536549	GTAATAGTCCTGAGTGCCTGGA	GTAATAGTAC--AGTGCCTGGA	NM_001009186, NM_001762	92	2	Type-IV	0	0	0
CpG	Non-CpG	chr8:74206167	chr8:71540398	GGGAGGAACG-GAGCCTAATC	GGGAGGAACCCGAGCCTAATC	NM_000971	92	1	Type-IIa	0	1	-1
CpG	Non-CpG	chr8:74206167	chr8:71540398	TCAGGGAGTT-----CTTTGCGTCT	TCAGGGAGTTGTTAATTTTGCGTCT	NM_000971	92	5	Type-IIb	0	0	0
CpG	Non-CpG	chr8:74206167	chr8:71540398	TTGATTGTGAACGCTGTGTGA	TTGTTTGTGA-CGCTTTGTGA	NM_000971	92	1	Type-IIa	0	0	0
CpG	Non-CpG	chr11:3239904	chr14:3054463	AGCAGGAAGT------------TCAGCAGGGG	AGCAGGAAGTGCAGCAGGAAGTTCAGCAGGGG	NM_173590	91	12	Type-IV	0	0	0
CpG	Non-CpG	chr12:82153393	chr11:80666558	TGAAGCAAGCAAAGAGGCTTGC	TGAAGCAAGC--AGAGGCTTGC	NM_003625	91	2	Type-IIa	0	0	0
CpG	Non-CpG	chr12:82153393	chr11:80666558	ACGGGAGCGCGCGGAAACAGGC	GCGGGAGCGC--GGAAACAGGC	NM_003625	91	2	Type-I	2	1	1
CpG	Non-CpG	chr1:155881472	chr1:130485298	AAGTTGTTAA-----TTTAAGTGTC	AAGTTGTTAAATGTTTTTAAGTGTC	NM_006912	91	5	Type-IIa	0	0	0
CpG	Non-CpG	chr1:155881472	chr1:130485298	CACGTACTCCCTTCCCTTTTGTGT	CACGTACTCC----CCTTTTGTGT	NM_006912	91	4	Type-IIb	0	0	0
CpG	Non-CpG	chr1:155881472	chr1:130485298	AACTGGGTTTTAGGGACCTGG	AACTGGGTTT-AGGGACCTGG	NM_006912	91	1	Type-IIa	0	0	0
CpG	Non-CpG	chr1:155881472	chr1:130485298	GAAGGAAGACGAAGTGCGTGA	GAAGGAAGAC-AAGTGGGTGA	NM_006912	91	1	Type-IV	1	0	1
CpG	Non-CpG	chr1:155881472	chr1:130485298	GGCCGCATCCCTTTCTTCCCAAAA	GGTCGCATCC----CTTCCCAAAA	NM_006912	91	4	Type-IIb	0	0	0
CpG	Non-CpG	chr1:156675675	chr1:131334090	CAGGTGGGGG--GGGGCGGAGC	CAAGTGGGGGAGGGGGCGGAGC	NM_001878	91	2	Type-IIa	0	0	0
CpG	Non-CpG	chr1:156675675	chr1:131334090	GCAAGGGGGGGCTCTGGAGTC	GCAAGGGGGG-CTCTGGAGTC	NM_001878	91	1	Type-IIa	0	0	0
CpG	Non-CpG	chr16:31885401	chr20:29692111	ATATACTTCTTCTTAAACTTAATTAGCTA	ATGTACTTCT---------TAATTAGCTA	NM_003414	91	9	Type-IIb	0	0	0
CpG	Non-CpG	chr16:31885401	chr20:29692111	GGTGAGTGTGCGGGGTCGGGG	GGTGAGTGTG-GGGGTCGGGG	NM_003414	91	1	Type-IIa	1	0	1
CpG	Non-CpG	chr17:42977397	chr16:50784511	AAGGGTTAGTTCTATGTATTAAATGCCTGG	AAGGGTTAGT----------AAATGCCTGG	NM_213607	91	10	Type-IV	0	0	0
CpG	Non-CpG	chr19:7953695	chr19:8069388	AAAGGTGGCCGTAGAATGGCTTTGGGAGT	AAGGGTGGCC---------CTTTGGGAGT	NM_025061	91	9	Type-IV	1	0	1
CpG	Non-CpG	chr19:44174621	chr19:38948154	AGGAGAGAAGACGTGCAGGGACC	AGGAGAGAAG---TGCAGGGACC	NM_001005377, NM_002659, NM_001005376	91	3	Type-IV	1	0	1
CpG	Non-CpG	chr19:44174621	chr19:38948154	GGATGAGTTGGAGGAATGCGG	GGATGAGTTG-AGGAATGGGG	NM_001005377, NM_002659, NM_001005376	91	1	Type-IIb	0	0	0
CpG	Non-CpG	chr3:123711250	chr2:45234111	CTGAGGCCAGGCGCCAGGTGTCGCCTGAAGCCAGACAGCCGGTT	CTGAGGCCAG------------------------ACAGCCGGTT	NM_017578	91	24	Type-IV	2	0	2
CpG	Non-CpG	chr3:125688362	chr2:45234111	CTGAGGCCAGGCGCCAGGTGTCGCCTGAAGCCAGACAGCCGGTT	CTGAGGCCAG------------------------ACAGCCGGTT	NM_001012337	91	24	Type-IV	2	0	2
CpG	Non-CpG	chr4:119810385	chr5:117880364	TTGGCTCTCCCTTCCGTCCTCCTT	TTGGCTCTCA----CGTCCTCCTT	NM_133477	91	4	Type-IIb	0	0	0
CpG	Non-CpG	chr4:119810385	chr5:117880364	CTAGGAAGGAAGGAATGAAAGCAA	CTAGGAAGGA----ATGAAAGCAA	NM_133477	91	4	Type-IIb	0	0	0
CpG	Non-CpG	chr6:37467969	chr4:38463096	AGCATGCGCACATAGCGACTTG	AGCATGCGCA--TAGCGACTTG	NM_138493	91	2	Type-I	0	0	0
CpG	Non-CpG	chr11:4629672	chr14:62971349	GCCAGGAGCT--------------GCTCCCTGAC	GCCAGGAGCTTACTGTCCCCAGCTGCTCCCTGAC	NM_018073	90	14	Type-IV	0	0	0
CpG	Non-CpG	chr12:75724136	chr11:74194267	TCAGCCAGGCAGCGGAACTGA	TCAGCCAGAC-GCGGAACTGA	NM_032606	90	1	Type-I	0	1	-1
CpG	Non-CpG	chr12:75724136	chr11:74194267	TTACCGAGCTTGTTAGGTGAA	TTACTGAGCT-GTTAGGTGAA	NM_032606	90	1	Type-IIa	0	0	0
CpG	Non-CpG	chr19:12251452	chr19:12141976	TTGGCTGAAAAGAAAATATCCAA	TTGGCTGAAA---AAATATCCAA	NM_021143	90	3	Type-IIb	0	0	0
CpG	Non-CpG	chr22:24891634	chr10:66650896	GGCCCTGGAG-GCTGATTGGC	GGCCCTGGAGCGCTGATTGGC	NM_016327	90	1	Type-I	0	1	-1
CpG	Non-CpG	chr1:247267934	chr2:103221858	TAGCTATAAC-TTGTATACA-	TAGCTATAACCTTGTATACAA	NM_024804	90	1	Type-IIa	0	0	0
CpG	Non-CpG	chr1:247267934	chr2:103221858	-TTGTATACA-GGTTAAATGT	CTTGTATACAAGGTTAAATGC	NM_024804	90	1	Type-IIa	0	0	0
CpG	Non-CpG	chr1:247267934	chr2:103221858	TAGGCCAAAAGCGGTCATTCT	TAGGCCAAAA-CGGTCATTCT	NM_024804	90	1	Type-IIa	0	0	0
CpG	Non-CpG	chr1:247267934	chr2:103221858	GCCTGAGGGG-CGGGGCCTGA	GCCTGAGGGGGCGGGGCCTGA	NM_024804	90	1	Type-IIa	0	0	0
CpG	Non-CpG	chr3:38066311	chr2:104722437	CTCCCCTCCAAGGGCGACAGT	CTCCCCTACA-GGGCCACAGT	NM_006225	90	1	Type-IIa	0	0	0
CpG	Non-CpG	chr3:38066311	chr2:104722437	ATCTGCGGGGGAATGGGAAGG	ATCTGCGGGG-AATGGGCAGG	NM_006225	90	1	Type-IIa	0	0	0
CpG	Non-CpG	chr8:72274601	chr8:69619465	TTTACATAAT-------TTTTTTTTT	TTTACATAATAATTTTTTTTTTTTTTT	NM_172059, NM_172060, NM_172058, NM_000503	90	7	Type-IIa	0	0	0
CpG	Non-CpG	chr8:72274601	chr8:69619465	AAAAAAGAAA-----------------------------------GCCCCGAGGC	AAAAAAAAAAAAAAAAAAAAGAAGAAGAAAAAGAAGAAAAAAAAAAAGCCCTGAGGC	NM_172059, NM_172060, NM_172058, NM_000503	90	35	Type-IIa	0	0	0
CpG	Non-CpG	chr20:47836106	chr10:15203111	CCTCCCCGTT-CGACCTCTTA	TCTCCCCGTTTCGACCTCTTA	NM_001037328, NM_017454, NM_017452, NM_017453, NM_004602	89	1	Type-IIa	0	0	0
CpG	Non-CpG	chr20:47836106	chr10:15203111	GGGCGTGGTTGGTTTCGTGTGACG	GGGCGTAGTT----TCGTATGACG	NM_001037328, NM_017454, NM_017452, NM_017453, NM_004602	89	4	Type-IIb	0	0	0
CpG	Non-CpG	chr2:3642722	chr13:3477786	AAGCCGGCCTCAGGGAGTTCC	AAGCCGGCCT-GGGGAGTTCC	NM_199235, NM_024027	89	1	Type-IIa	0	0	0
CpG	Non-CpG	chr2:3642722	chr13:3477786	GTTCCAGGCG---------TTCCCACACT	GTTCCTGGCGCTGCCGGCGTTCCCACACT	NM_199235, NM_024027	89	9	Type-IV	0	2	-2
CpG	Non-CpG	chr2:3642722	chr13:3477786	CTGCGGCCGG----GCGGCCTGAT	CTGCGGCCGGCCGGGCGGCCTGAT	NM_199235, NM_024027	89	4	Type-IIb	0	1	-1
CpG	Non-CpG	chr2:44001488	chr13:44494550	AATAAAAGAA------------------AATTGCTTTT	AATAAAAGAACATTTCCAGCCCAGAAACAATTGCTTTT	NM_001012665, NM_015522, NM_016008	89	18	Type-IIa	0	0	0
CpG	Non-CpG	chr2:44001488	chr13:44494550	CAGCCAGGCCGTCGACGATGCCCA	CAGCCCGGCC----ACGATGCCCA	NM_001012665, NM_015522, NM_016008	89	4	Type-IV	2	0	2
CpG	Non-CpG	chr2:44001488	chr13:44494550	TGGGGGTGGC-TACTTGCCAC	AGGGGGTGGCCTGCTTGCCAC	NM_001012665, NM_015522, NM_016008	89	1	Type-IIa	0	0	0
CpG	Non-CpG	chr2:44001488	chr13:44494550	ACAGGAAAGAAAAGAAAGTTCCCTA	ACAGGAAAGA-----AAGTTCCCTA	NM_001012665, NM_015522, NM_016008	89	5	Type-IIb	0	0	0
CpG	Non-CpG	chr3:195621901	chr2:202592746	AAGGTCGGGCTGTTTTCCTGG	AAGGTCGGGC-ATTTTCCTGG	NM_001010938, NM_005781	89	1	Type-IV	0	0	0
CpG	Non-CpG	chr3:195621901	chr2:202592746	GGGCCGGGCCGGGCCGGGCGCGGTG	GGGCCAGGCC-----GGGCGCGGTG	NM_001010938, NM_005781	89	5	Type-IIb	2	1	1
CpG	Non-CpG	chr7:10980062	chr3:97676731	GTTTTCCCTTGTCCTTCTTGCGT	GTTTTTCCTT---CTTCTTGCGT	NM_002489	89	3	Type-IIb	0	0	0
CpG	Non-CpG	chr9:707303	chr15:83844771	AACAGTGGGGCTGATTGTGCC	AACAGTGGGG-TGATTGTGCC	NM_015158, NM_153186	89	1	Type-IV	0	0	0
CpG	Non-CpG	chr19:36024614	chr19:31453875	AGGATCGCTT-GAGCCCAGGA	AGGATCGCTTTGAGCCCAGGA	NM_014364	88	1	Type-IIa	0	0	0
CpG	Non-CpG	chr19:36024614	chr19:31453875	ACTGCACTCCAGCCTAGGAGA	ACTACACTCC-GCCTAGGAGA	NM_014364	88	1	Type-IV	0	1	-1
CpG	Non-CpG	chr19:36024614	chr19:31453875	CAGGACCCAC-AGCCCTGGCG	CCGGACCCACCAGCCCTGGCG	NM_014364	88	1	Type-IIb	0	0	0
CpG	Non-CpG	chr19:36024614	chr19:31453875	CAGCAGGTCC----------AGGCCAATGA	CGGCAGGTCCCGGGGCAGCCAGGCCAATGA	NM_014364	88	10	Type-IIa	0	1	-1
CpG	Non-CpG	chr6:28235221	chr4:28685280	AATGAGAAAACAAAAACAAAAACAGAAACAAAAAAA	AATGAGAAAG----------------AACTAAAAAA	NM_007151, NM_001023560, NM_152736	88	16	Type-IIb	0	0	0
CpG	Non-CpG	chr1:6051680	chr1:5113484	CATACAAAAA--GACACAAGTG	CACACAAAAAAAGACACAAGTG	NM_172130, NM_003636	88	2	Type-IIa	0	0	0
CpG	Non-CpG	chr1:6051680	chr1:5113484	GTCCACCAAAGCCACACATCG	GTCCACCAAA-CCACACATCG	NM_172130, NM_003636	88	1	Type-IIa	0	0	0
CpG	Non-CpG	chr7:37960542	chr3:70826414	GAAGCGGCAGAAGGCAGTGGCAGCAGGCAGTGG	GAAGCGGCAG-------------CAGGCAGTGG	NM_017549	88	13	Type-IIc	0	0	0
CpG	Non-CpG	chr11:721375	chr14:572077	GCAGGAGGAG---CCGCGGGCCG	GCAGGAGGAGGAGCCGCGGGCTG	NM_022772	86	3	Type-IIb	0	0	0
CpG	Non-CpG	chr4:78784281	chr5:56674305	AGAAAAAGAATAAGAAGGAAGGA	AGAAAAAGAG---GAAGAAAGGA	NM_020236	86	3	Type-IIb	0	0	0
CpG	Non-CpG	chr5:1202010	chr6:1104432	GT-GCAGGGG--GAGGGGTGGC	GTTGCAGGGGTGGAGGGGTGGC	NM_001003841	86	2	Type-IIa	0	0	0
CpG	Non-CpG	chr5:1202010	chr6:1104432	GAGGGGTGGCCAGCGCCCACGGAG	GAGGGGTGGC----GCGCACGGAG	NM_001003841	86	4	Type-IV	1	1	0
CpG	Non-CpG	chr5:1202010	chr6:1104432	AGGCATTAACAGTCCCTCCGC	AGGCGTTAAC-GTACCTCCGC	NM_001003841	86	1	Type-IV	0	1	-1
CpG	Non-CpG	chr5:1202010	chr6:1104432	CAGGTGCCTG---------GGGCGAGGTG	CAGGTGCGTGCGGCGGGTGGGGCGAGGTG	NM_001003841	86	9	Type-IIa	0	2	-2
CpG	Non-CpG	chr3:12838477	chr2:53614027	TGGGGGTGTGTGCAATCCGGCG	TGGGGGTGTG--CAAGCCGGCG	NM_012298	85	2	Type-I	0	0	0
CpG	Non-CpG	chr3:12838477	chr2:53614027	CCGGCCCCCT-TCTCTCCTTC	CCGGCCCCCCGCCTCTCCTTC	NM_012298	85	1	Type-IIb	0	1	-1
CpG	Non-CpG	chr19:11690038	chr19:11628205	TGGGCGGTGCAGGGTTCCGGG	TGGGCGGTGC-GGGTTCCGGG	NM_001611	84	1	Type-IV	0	1	-1
CpG	Non-CpG	chr19:11690038	chr19:11628205	TGGGGCCGCC-AGGAGTGCGA	TGGGGCCGCCCAGGAATGCGA	NM_001611	84	1	Type-IIa	0	0	0
CpG	Non-CpG	chr19:11690038	chr19:11628205	CCCGCCCCGC-GACGGCAGGT	CCCGCCCCGCCGATGGCAGGT	NM_001611	84	1	Type-IIb	1	1	0
CpG	Non-CpG	chrX:111923455	chrX:106673560	TCAGCCCCCCCTTCTCCCCTT	TCAGCCCCCC-TTCTCCCTTT	NM_178175	84	1	Type-IIa	0	0	0
CpG	Non-CpG	chr11:790423	chr14:636119	CCCACCGACCCCTCACCCACCGACCCGTCACCCACCGACCAAGGGGCACC	CCCACCGACC------------------------------AAGGGGCACC	NM_016564	83	30	Type-IIc	3	0	3
CpG	Non-CpG	chr11:790423	chr14:636119	CCCCCTCCCC------------GCCCTCCTGC	CCCCCTCCCCCTCCCCCTCCCCGCCCTCCTGC	NM_016564	83	12	Type-IIb	1	1	0
CpG	Non-CpG	chr11:790423	chr14:636119	CCGAGCTCTCCTCCGCGTTCGCA	CCGAGCTCTC---CGCGTTCGCA	NM_016564	83	3	Type-IIb	0	0	0
CpG	Non-CpG	chr11:790423	chr14:636119	GCGGGGCGGGCGGGGCGGGCGCGG	GCGGGGCGGG----GCAGGCGCGG	NM_016564	83	4	Type-IIb	1	0	1
CpG	Non-CpG	chr17:62039354	chr16:57580700	CTCACTGAGGCGGTGGAGCTTGGAGC	CTCACTGAGG------AGCTTGGAGC	NM_000334	83	6	Type-IV	1	0	1
CpG	Non-CpG	chr17:62039354	chr16:57580700	AGCTTGGAGCCGGCCGCGCTCAGAGCGCG	AGCTTGGAGC---------TCAGAGGGCG	NM_000334	83	9	Type-IIb	3	0	3
CpG	Non-CpG	chr17:62039354	chr16:57580700	GGTTACAGGGGAGGTGACAGT	GGTTACAGGG-AGGTGACAGT	NM_000334	83	1	Type-IIa	0	0	0
CpG	Non-CpG	chr17:62039354	chr16:57580700	AGGTGGAACAAAAGCTCTAGCTGGAGCCGGGGGCACGTG	AGGTGGAACC-------------------GGGGCACGCG	NM_000334	83	19	Type-IV	1	1	0
CpG	Non-CpG	chr7:64839064	chr19:22709858	GACGCCAGGA-CCCCCTGGAA	GACGCCAGGGTCCCCCTGGAA	NM_152626, NM_007139	83	1	Type-IV	0	0	0
CpG	Non-CpG	chr7:64839064	chr19:22709858	TGTGGCGGGCCTCGGGCCTTC	TGTGGCGGAC-TCGGGCCTCC	NM_152626, NM_007139	83	1	Type-IIa	0	0	0
CpG	Non-CpG	chr7:64839064	chr19:22709858	TCTCCTTGGCGCAGCTCGGCC	TCTCCTTGCC-CAGTTCGGCC	NM_152626, NM_007139	83	1	Type-IIb	1	0	1
CpG	Non-CpG	chr11:77013011	chr14:69486163	TAAACTTAAACAGTCAAGTTCTTTTTG	TAAACTTACA-------GTTCTTTTTG	NM_182833	82	7	Type-IV	0	0	0
CpG	Non-CpG	chr11:77013011	chr14:69486163	TAAGAACCAAACCAAACCAAA	TAAGAACCAA-CCAAACCAAA	NM_182833	82	1	Type-IIa	0	0	0
CpG	Non-CpG	chrY:16636999	chrY:4143421	CAGGCGTACAGTTCTGGAGCC	CAGGCGTCCA-TTCTGGAGCT	NM_014893	82	1	Type-IV	0	0	0
CpG	Non-CpG	chr2:170430622	chr12:56519901	CTATATCTTCTTCAACAGCTTAA	CTATATCTTC---AACAGCTTAA	NM_024622	82	3	Type-IIb	0	0	0
CpG	Non-CpG	chr2:170430622	chr12:56519901	GAATTCCCCC-AAAATGCGTG	GAATTCCCCCCAAAATTCGTG	NM_024622	82	1	Type-IIa	0	0	0
CpG	Non-CpG	chr7:76027141	chr3:41011180	CGCCCATCGC--------------CCCTAGAGCC	CGCCCACCGCTCACCGCCCAAAGCCCCTAGAACC	NM_007155	82	14	Type-IIa	0	1	-1
CpG	Non-CpG	chrX:101771964	chrX:96319066	ACCAAGCCCC-GATCCTAGAG	ACCAAGCCCCTGATCCTGGAG	NM_021992	81	1	Type-IV	1	0	1
CpG	Non-CpG	chrX:101771964	chrX:96319066	AGGGGAAGGGCGGGGTGGGGGGGGGGGGTGGCGAGCCCG	AGGGGAAGGG--------------------GGCGAGCCCG	NM_021992	81	20	Type-IIa	1	0	1
CpG	Non-CpG	chrX:119078035	chrX:113732456	GAAATATATA----------------CTTCCCTTCA	GAAATATATATTCAGTGAAATATATACTTCCCTTCA	NM_024528	81	16	Type-IV	0	0	0
CpG	Non-CpG	chrX:119078035	chrX:113732456	GGCAACAGAC-ATCTTCCTAG	GGCAACAGATTATCTTCCTAG	NM_024528	81	1	Type-IV	0	0	0
CpG	Non-CpG	chr5:156570178	chr6:155295526	AAGATTGTTT-CTGGTCAATA	AAGATTTTTTTCTAGTCAATA	NM_004270	81	1	Type-IIa	0	0	0
CpG	Non-CpG	chr5:156570178	chr6:155295526	TATGCCTTAATTCAAAAAGAAACGAAG	TATGCCTTAA-------AGAAACGAAG	NM_004270	81	7	Type-IV	0	0	0
CpG	Non-CpG	chr5:156570178	chr6:155295526	TGCAGAGGGG-AAGGCGGCTA	TGCAGAGGGGGAAGGCGGCTA	NM_004270	81	1	Type-IIa	0	0	0
CpG	Non-CpG	chr5:156570178	chr6:155295526	TCTGTGCTGG-GCTGGGAACT	TCTGTGCTGGTGCTGGGAACT	NM_004270	81	1	Type-IIb	0	0	0
CpG	Non-CpG	chr16:28857627	chr20:27351581	GCCTGACTCC-AAGCTTCGGT	GCCTGACTCCCAAGCTTCGGT	NM_003321	80	1	Type-IIa	0	0	0
CpG	Non-CpG	chr19:52408570	chr19:47134849	AGCATAGAAG---CGCGAGACTT	AGCATAAAAGGACCACGAGACTT	NM_023074	80	3	Type-I	0	0	0
CpG	Non-CpG	chr19:52408570	chr19:47134849	GTTTGTTCAGATAAACTCTAG	GTTTGTTCAG-TAAACTCTAG	NM_023074	80	1	Type-IV	0	0	0
CpG	Non-CpG	chr19:52408570	chr19:47134849	CATCCCGGTTTAATGACACAA	CATCCCGGTT-AATGATACAA	NM_023074	80	1	Type-IIa	0	0	0
CpG	Non-CpG	chr19:52408570	chr19:47134849	GGCCAGTGCC------GGGGTTTAAG	GGCCGGTGCCAATGCCGGGGTTTAAG	NM_023074	80	6	Type-IIa	1	1	0
CpG	Non-CpG	chr19:57106964	chr19:52591029	CTCGGCTGGG-------CTCGGCTGAA	CTCTGCTGGGGGCAGGGCTCGGCTGAA	NM_021216	80	7	Type-IIa	0	0	0
CpG	Non-CpG	chrX:15511756	chrX:14799540	CCCGCCTCCC-------GCCTCCTCTA	CCCGCCTCCCTCCTCCCGCCTCCTCTA	NM_003662, NM_001018109	80	7	Type-IIb	1	1	0
CpG	Non-CpG	chrX:15511756	chrX:14799540	CGCGTCACCC-CCGCCGCTAA	CGCGTCCCCCGCCGCCGCTAA	NM_003662, NM_001018109	80	1	Type-IIa	0	1	-1
CpG	Non-CpG	chr5:158526773	chr6:157273701	AAAAAAAAAAAATCTCTGGTTG	AAAAAAAAAA-TCTCTGGTTG	NM_024007	80	1	Type-IIa	0	0	0
CpG	Non-CpG	chr5:158526773	chr6:157273701	TAATTTTTTT--CAAGGGGGAG	TAATTTTTTTTTTCAAGGGGGAG	NM_024007	80	2	Type-IIa	0	0	0
Non-CpG	CpG	chrX:9431635	chrX:8531203	TCATGAGCTC----GTTCGGGTTT	TCATGAGCTCTTTCGCTCGGGTTT	NM_005647	97	4	Type-IIa	1	1	0
Non-CpG	CpG	chrX:9431635	chrX:8531203	CATGTAATACCTCAGCATCAG	CATGTAATAG-TCAGCATCAG	NM_005647	97	1	Type-IV	0	0	0

Non-CpG	CpG	chrX:9431635	chrX:8531203	GTATTTTTTT-ATTGATGTCC	GTATTTTTTTTATTGATGTCC	NM_005647	97	1	Type-IIa	0	0	0
Non-CpG	CpG	chrX:102941927	chrX:97432346	CGGGCTAAAAAAAAAAACCTAAGAGG	CGGGCTAAAA------ACCTAAGAGG	NM_012286	97	6	Type-IIa	0	0	0
Non-CpG	CpG	chr5:133860303	chr6:132238108	TGGATAATCT--GAGGGGCAGC	TGGATAATCTTTGAGGGGCAGC	NM_015288	97	2	Type-IIa	0	0	0
Non-CpG	CpG	chr5:133860303	chr6:132238108	AGGCCAGACCCACCCCCTCCC	AGGCCACACC-ACCCCCTCCC	NM_015288	97	1	Type-IIa	0	0	0
Non-CpG	CpG	chr5:133860303	chr6:132238108	GTTTTTAACC-TTTCGGGGTA	GTTTTTAACCCTTTCGGGGTA	NM_015288	97	1	Type-IIa	0	0	0
Non-CpG	CpG	chr11:77123297	chr14:69600616	GGGATCTCCGCCGGCTTTCGGAG	GGGATCTCCG---GCTTTCGGAG	NM_002576	96	3	Type-IIb	1	0	1
Non-CpG	CpG	chr12:72058389	chr11:70474180	TGGAGCTGCT----GTTGCAGCCT	TGGAGCTGCTCTTAGTTGCATCCT	NM_031435	96	4	Type-I	0	0	0
Non-CpG	CpG	chr12:72058389	chr11:70474180	GGGAAAACACCTCTTCCTTTTAA	GGGAAAACAC---TTCCTTTTAA	NM_031435	96	3	Type-IIb	0	0	0
Non-CpG	CpG	chr1:155658029	chr1:130256589	GGAGGCCGAG------AGCGATGAGA	GGAGGCCGAGGCCGAGAGCGACGAGA	NM_004632, NM_033657	96	6	Type-IIc	0	1	-1
Non-CpG	CpG	chrX:43515851	chrX:43707638	AGGGGTAGGGGAACCTACAGT	AGGGGTAGGG-AACCTGCAGT	NM_000240	96	1	Type-IIa	0	0	0
Non-CpG	CpG	chr1:52521689	chr1:51640719	TTTTCACCTGTTTGTTATACAGCG	TTTTCACCTG----TTATACAGCG	NM_015913	96	4	Type-IIb	0	0	0
Non-CpG	CpG	chr1:52521689	chr1:51640719	GCTGGACTGGGTGGCGGACGC	GCTGGACTGG-TGGCGAACGC	NM_015913	96	1	Type-IIa	0	0	0
Non-CpG	CpG	chr8:91095407	chr8:88620868	TCTCGCTCCAGCTAGCTTTCC	TCCCGCTCCA-CTAGCTTTCC	NM_004929	96	1	Type-IV	0	0	0
Non-CpG	CpG	chr10:24498390	chr9:24384863	GGACGGACGG----ACAGACCTAG	AGACGGACGGTCGGACAGACCTAG	NM_019590	95	4	Type-IIc	0	1	-1
Non-CpG	CpG	chr10:24498390	chr9:24384863	AAAAAAAAAA-GGCAATTTCT	AAAAAAAAAAAAGGCAAATTCT	NM_019590	95	1	Type-IIa	0	0	0
Non-CpG	CpG	chr12:56212125	chr11:54555121	CCATAGCCGG-ACGGGGATCT	CCATAGCCGGGACGGGGATCT	NM_014182	95	1	Type-IIa	0	0	0
Non-CpG	CpG	chr12:56212125	chr11:54555121	GGCAGGTAGGAGCTGCAAAGA	GGCAGGTAGG-GCTGCAAAGA	NM_014182	95	1	Type-IIa	0	0	0
Non-CpG	CpG	chr14:23306094	chr7:84164735	CTGGATCCCCCTACAGCCCCC	CTGGGTCCCC-TACAGCCCCC	NM_004995	95	1	Type-IIa	0	0	0
Non-CpG	CpG	chr14:23306094	chr7:84164735	AACTTAGAGG-----TGTTTTTTTT	AACTTAGCGGGTTGGTTTTTTTTTT	NM_004995	95	5	Type-IIa	0	0	0
Non-CpG	CpG	chr14:23306094	chr7:84164735	AGAGCGAGAGAGGGAACCAGAC	AGAGCGAGAG--GGATCCAGAC	NM_004995	95	2	Type-I	0	0	0
Non-CpG	CpG	chr14:23306094	chr7:84164735	CAATCCCCTTTAACTCCAAGC	CAATCCCCTT-AATTCCAAGC	NM_004995	95	1	Type-IIa	0	0	0
Non-CpG	CpG	chr14:23306094	chr7:84164735	AGTTCAGTGCCTACCGAAGAC	AGTTCAGTGC-TACCGAAGAC	NM_004995	95	1	Type-IIa	0	0	0
Non-CpG	CpG	chr14:81408369	chr7:143445198	GGTCCTTTAACAAAAAAAAAAAA	CGTCCTTTAA---AAAAAAAAAA	NM_152446	95	3	Type-IIa	0	0	0
Non-CpG	CpG	chr14:81408369	chr7:143445198	GATCAGGCTT-TTATCCGGGA	GATCAGGCTTCTTTTCCGGGA	NM_152446	95	1	Type-IIa	0	0	0
Non-CpG	CpG	chr16:19191891	chr20:18330557	CTATAAAACAACCAGACGGTT	CTATAAACCA-CCAGACGGTT	NM_016524	95	1	Type-IIa	0	0	0
Non-CpG	CpG	chr16:19191891	chr20:18330557	ACCACACTGT--CAAATTTGCA	ACCACACTGTTTCAAATTTGCA	NM_016524	95	2	Type-IIa	0	0	0
Non-CpG	CpG	chr4:129731674	chr5:128136288	CGGCGGCGGC------TGCAGGACGA	CGGCGGCGGCGGCGGCTGCAGGACGA	NM_024900, NM_199320	95	6	Type-IIb	0	2	-2
Non-CpG	CpG	chr4:129731674	chr5:128136288	GGCGGGGGCC---GGAGGAGGAG	GGCGGGGGCCAGAGGAGGAGGAG	NM_024900, NM_199320	95	3	Type-I	1	0	1
Non-CpG	CpG	chr8:120428986	chr8:118611991	CCTGTGCTGG---------GCGTGATCGG	CCTGTGCTGGTGGTGCTGGGCGTGATCCG	NM_002514	95	9	Type-IIb	0	0	0
Non-CpG	CpG	chr8:120428986	chr8:118611991	AAGATGCCCCCAAGTTACTTT	AGGATGCCCC-AAGTTACTTT	NM_002514	95	1	Type-IIa	0	0	0
Non-CpG	CpG	chr8:120428986	chr8:118611991	GTTTCTCCTT---GTCTCGCCTG	GTTTCTCCTTCTTGTCTCGCCTG	NM_002514	95	3	Type-IIb	0	0	0
Non-CpG	CpG	chr8:120886257	chr8:119071567	GGTAAAGAGA-GCTCACCAAA	GGTAAAGAGAAGCTCACCAAA	NM_022783	95	1	Type-IIa	0	0	0
Non-CpG	CpG	chr8:120886257	chr8:119071567	ACGGCCCTAAAACCATGGAGGA	ACGGCCCCAA--CCATGGAGGA	NM_022783	95	2	Type-IIa	0	0	0
Non-CpG	CpG	chr9:125694091	chr15:16163743	TCGGGGCCCCTGTTGTCCTCC	TCGGGGCCCC-GTTGTCCTCC	NM_020924	95	1	Type-IIa	0	1	-1
Non-CpG	CpG	chr12:100594894	chr11:100107878	CACACACACACACACGCAGCC	CACACACACA-GCACGCAGCC	NM_022496	94	1	Type-I	0	0	0
Non-CpG	CpG	chr12:100594894	chr11:100107878	GAAGCACAGC-------CCCTAACATT	GAAGCACAGCGGACACTCCCTAACAGT	NM_022496	94	7	Type-IIa	0	1	-1
Non-CpG	CpG	chr15:34517519	chr7:10145620	GTCTTGCCTGGTTTTTTTTTCC	GTCTTGCCTG-CTTTTTTTCC	NM_016454	94	1	Type-IIa	0	0	0
Non-CpG	CpG	chr15:34517519	chr7:10145620	AAATGACGTC-GTATTCACGC	AAATGACGTCTGTATTAACGC	NM_016454	94	1	Type-IV	1	0	1
Non-CpG	CpG	chr15:78556787	chr7:55662557	CCCCTAAAAAATGCAACAATC	CCCCTAAAAA-TGCAACAATC	NM_018602	94	1	Type-IIa	0	0	0
Non-CpG	CpG	chr16:72128123	chr20:56672669	GGAAGTGTCT--GATCAGAGCC	GGAAGTGTCTCTGATCAGAGCC	NM_014003	94	2	Type-I	0	0	0
Non-CpG	CpG	chr16:72128123	chr20:56672669	CAGGAATCGGGCGTGTTCCAG	CAGGAATCGG-CGTGTTCCAG	NM_014003	94	1	Type-IIa	0	0	0
Non-CpG	CpG	chr16:72128123	chr20:56672669	TAGAGGGGGC-GCGCAAAGTA	TAGAGGGGGCCGCGCAAGGTA	NM_014003	94	1	Type-IIa	1	1	0
Non-CpG	CpG	chr16:72128123	chr20:56672669	GTCCGGGCCCCAGTTTGGGGAG	GTCCGGGCCC--GTTTGGGGAG	NM_014003	94	2	Type-IIa	0	1	-1
Non-CpG	CpG	chr17:73453031	chr16:69159682	GCCAGAATTC-CCCCTATTCT	GCCAAAATTCACCCCCATTCT	NM_014738	94	1	Type-IIa	0	0	0
Non-CpG	CpG	chr17:73453031	chr16:69159682	TGAAGTGAGGAGGGGGTTGGGAG	TGAAGTGAGG---GGGCTGGGAG	NM_014738	94	3	Type-IIa	0	0	0
Non-CpG	CpG	chr19:12835046	chr19:12671075	GAACCCCCAAATCCAGCCCCA	GAACCCCCAA-TCCAGCCCCA	NM_013433	94	1	Type-IIa	0	0	0
Non-CpG	CpG	chr19:12835046	chr19:12671075	GGGTGGGGGAGAGGGAGGCCT	GGGTGGGGGA-AGGGAGGCCT	NM_013433	94	1	Type-I	0	0	0
Non-CpG	CpG	chr19:12835046	chr19:12671075	CTCTGCCTCTCTCGAGGGTCAA	CTGTGCCTCT--CGAGGGTCAA	NM_013433	94	2	Type-I	0	0	0
Non-CpG	CpG	chrX:133941567	chrX:128783005	AGGGCTGAGAGAGATGGAGAAGGA	AGGGCTGAGA----TGGAGAAGGA	NM_138819	94	4	Type-I	0	0	0
Non-CpG	CpG	chr2:18059414	chr13:17831282	GTCCAAGCACCTCTTCTGACTCG	GTCCAGGCAC---TTCTGACTCG	NM_002252	94	3	Type-I	0	0	0
Non-CpG	CpG	chr12:113573841	chr11:113165189	TTGGAGGCGGGATTGTGGGGA	T-GGAGGCGG-ATTGGGGGGA	NM_004658	93	1	Type-IIa	0	0	0
Non-CpG	CpG	chr12:113573841	chr11:113165189	AGGTCCCTGGGCCGCCCCTCC	AGGTCCCTGG-CCGCCCCTCC	NM_004658	93	1	Type-IIa	0	0	0
Non-CpG	CpG	chr21:38353564	chr3:9754101	GCAGTTATCAAGGTGGGTGGG	GCAGTTATCA-GATGGGTGGG	NM_000411	93	1	Type-IIa	0	0	0
Non-CpG	CpG	chr3:137894068	chr2:163792484	CAGGAAAACTTAAACTGAGCT	CAGGAAAACT-AAACTGAGTT	NM_016216	93	1	Type-IIa	0	0	0
Non-CpG	CpG	chr3:137894068	chr2:163792484	ATGTATATAC-AAAAAAATAT	ATGTATATACGAAAAAAATAT	NM_016216	93	1	Type-IV	0	1	-1
Non-CpG	CpG	chr3:137894068	chr2:163792484	AAAAAGAAAAAAGGAAAAAAAGAAAATAA	AAAAAGAAAA---------AAGAAAATAA	NM_016216	93	9	Type-IIb	0	0	0
Non-CpG	CpG	chr3:137894068	chr2:163792484	AGTCTACACCCAGGCGGTGGC	CGTCTACACC-AGGCTGTGGC	NM_016216	93	1	Type-IIa	0	0	0
Non-CpG	CpG	chr4:87515527	chr5:84939391	GCCTGCAGTGTGGTGAACTCCAA	GCCTGTAGTG---TGAACTCCAA	NM_138981, NM_138982, NM_138980, NM_002753	93	3	Type-IIb	0	0	0
Non-CpG	CpG	chr4:88312629	chr5:85751748	GAGAAAGTGGTGTAAAAGACA	GAGAAAGTGG-GTAAAAGATA	NM_016245	93	1	Type-I	0	0	0
Non-CpG	CpG	chr6:110299814	chr4:158001726	CCGAGTTCCC-AGGGAACTTT	CCGAGTTCCCCAGGGAACTTT	NM_005284	93	1	Type-IIa	0	0	0
Non-CpG	CpG	chr6:110299814	chr4:158001726	AGGATGTGGGGAGAGGAGGGGAGAAAGATA	AGGATGTGGG----------GAGAAGGATA	NM_005284	93	10	Type-IIb	0	0	0
Non-CpG	CpG	chr10:29811910	chr9:30867937	GAATGTTAAG-GTATTATTTT	GAATGTTAAAAGTATTGTTTT	NM_003174, NM_021738	92	1	Type-IIa	0	0	0
Non-CpG	CpG	chr10:29811910	chr9:30867937	TCCAGGAAGTTTGGTTTTCTCACAGAGTGTAGCGTGCT	TCCGGGAAGT------------------GTAGCGTGCT	NM_003174, NM_021738	92	18	Type-IV	0	0	0
Non-CpG	CpG	chr11:57336093	chr14:16078041	CCACAGCTTG-TAATGGACCA	CCATAGCTTGATAATGGACCA	NM_198183, NM_004223	92	1	Type-I	0	0	0
Non-CpG	CpG	chr11:57336093	chr14:16078041	GCTTCCCCCA-CCAACTGTTT	GCTTCCCCCAGCCAACTGTTT	NM_198183, NM_004223	92	1	Type-IV	0	0	0
Non-CpG	CpG	chr11:118492391	chr14:111479137	GAATGAGACC-TGTCCTTTGC	GAATGAGACCCTGTCCTTTGC	NM_015157	92	1	Type-IIa	0	0	0
Non-CpG	CpG	chr11:118492391	chr14:111479137	TCTTCACTCT--GGCCTCTGTA	TCTTCACTCTCTGGCCTCTGTA	NM_015157	92	2	Type-I	0	0	0
Non-CpG	CpG	chr11:118492391	chr14:111479137	CCCTCCCTCCCTGGCTCCCTT	CCCTCCCTCC-TGGCTCCCTT	NM_015157	92	1	Type-IIa	0	0	0
Non-CpG	CpG	chr11:118492391	chr14:111479137	TCCCTTCCCCCGCCGCTGGTG	TCCCTTCCCC-GCCGCTGGTG	NM_015157	92	1	Type-IIa	1	1	0
Non-CpG	CpG	chr11:118492391	chr14:111479137	CCCTCCCCGGGCATTGGCGGC	CCCTCCCCGG-CATTGGCGGC	NM_015157	92	1	Type-IIa	0	0	0
Non-CpG	CpG	chr11:118492391	chr14:111479137	GCCCCCTCAAAGGCTCCGGGCG	GCCCCCTCAA--GCCCCGGGCG	NM_015157	92	2	Type-IIa	0	0	0
Non-CpG	CpG	chr11:118492391	chr14:111479137	CTCCGGGCGGGCCCGGGACCTGG	CCCCGGGCGG---CGGGACCTGG	NM_015157	92	3	Type-IIb	0	0	0
Non-CpG	CpG	chr11:118492391	chr14:111479137	TTCGGCGGTG-AGTCCTCAGC	TTCGGCGGTGGAGTCCTCAGC	NM_015157	92	1	Type-IIa	0	0	0
Non-CpG	CpG	chr11:118492391	chr14:111479137	TCAGCCCCGGGCCAGCCACAG	TCAGCCCCGG-CCAGCCACAG	NM_015157	92	1	Type-IIa	0	0	0
Non-CpG	CpG	chr16:67195323	chr20:51437471	GGGGAAGGAG------------CCTCCCCTCC	GGGGAAGGAGGCGGGGAAAGAGCCTCCCCTCC	NM_018378	92	12	Type-IIa	0	1	-1
Non-CpG	CpG	chr19:21203763	chr19:20744578	CACATCTTCC-GTCAATCATG	CACATCTTCCAGTCAATCAGG	NM_025189	92	1	Type-IV	1	0	1
Non-CpG	CpG	chr19:21203763	chr19:20744578	CCGACATCCCCAGAGAGGGGAG	CCGACATTCC--GAGAGGGGAG	NM_025189	92	2	Type-IIa	0	1	-1
Non-CpG	CpG	chr19:21203763	chr19:20744578	CCCGAGTTCT--TGCCCAGCTG	CCCGAGTTCTCTTGCCCAGCTC	NM_025189	92	2	Type-I	0	0	0
Non-CpG	CpG	chrX:54069880	chrX:53459354	CCTGTGAAACAGGGGTCAGCTA	CTTGTGAAAC--GGGTCAGCTA	NM_015107	92	2	Type-IIa	0	1	-1
Non-CpG	CpG	chrX:54069880	chrX:53459354	CGAAGAAGGT--------------GGGGGGTGGG	-GAAGAAGGTCGGGGGTGGGGGGTGGGGGGTGGG	NM_015107	92	14	Type-IIa	0	1	-1
Non-CpG	CpG	chrX:54069880	chrX:53459354	CGGTAACTCT--ATTGGAGAAC	CGGTAACTCTCTATTGGGGAAC	NM_015107	92	2	Type-I	0	0	0
Non-CpG	CpG	chrX:100915135	chrX:95844703	AAAATTCCCCCAGATTCGGGC	AAAATTTCCC-AGACTCGGGC	NM_014782, NM_177949	92	1	Type-IIa	0	0	0
Non-CpG	CpG	chr2:55459729	chr13:56217743	AAAAGGAGGT----CGAGGGGGGC	AAAAGGAGGTGGGTCGAGGGGGGC	NM_152385	92	4	Type-IIa	0	0	0
Non-CpG	CpG	chr2:55459729	chr13:56217743	GGGGGGCCTC-TTGGGGAAGA	GGGGGGCCTCATTGGGGAAGA	NM_152385	92	1	Type-IV	0	0	0
Non-CpG	CpG	chr4:80994683	chr5:54384566	CAGCGGGGGGGACTTCAGCCC	CAGCGGGGGA-ACTTCAGCCC	NM_058172	92	1	Type-IIa	0	0	0
Non-CpG	CpG	chr6:57086524	chr4:58221323	TAGACTCCTC-----CCTGCAGTGC	TAGACTCCTCTTCTCCCTGCAGTGC	NM_183227, NM_016277	92	5	Type-IIb	0	0	0
Non-CpG	CpG	chr6:57086524	chr4:58221323	CAAATATAACCTGTACTTCTA	CAAATATAAC-TGTACTTCTA	NM_183227, NM_016277	92	1	Type-IIa	0	0	0
Non-CpG	CpG	chr7:91510307	chr3:102017803	ACAGTTCCCC-ACGGACATCT	ACAGTTCCCCCACGGCCATCT	NM_006980	92	1	Type-IIa	0	0	0
Non-CpG	CpG	chr7:143580246	chr3:175294724	CCAGTCACTG--GTACAGGGAA	CCAGTCACTGTGGTACAGGGAA	NM_014719	92	2	Type-I	0	0	0
Non-CpG	CpG	chr7:143580246	chr3:175294724	TGCGCCAGGCTCTGCCCGCTCC	TACGCCAGGC--TGCCCGCTCC	NM_014719	92	2	Type-I	0	0	0
Non-CpG	CpG	chr9:131217764	chr15:10412113	AGACCGGGCG----CGGAGGCTCA	AGGCCGGGCGGGCGCGGTGGCTCA	NM_002540, NM_153437	92	4	Type-IIb	0	1	-1
Non-CpG	CpG	chr12:49742076	chr11:47616357	GCACAAGGTG--TCTGGGGACC	ACACAAGGTGAGTCTGGGGACC	NM_024902	91	2	Type-IV	0	0	0
Non-CpG	CpG	chr12:75784981	chr11:74254956	AGGGAGGAGGGAAAAACAAAG	GGGGAGGAGG-AAAAACAAAG	NM_032606	91	1	Type-IIa	0	0	0
Non-CpG	CpG	chr12:75784981	chr11:74254956	GAAGAGGGAG--------------GGGAGAGTGG	GAGGAGGGAGAGGATACTGAGGAGGGGAGAGTGG	NM_032606	91	14	Type-IIb	0	0	0
Non-CpG	CpG	chr12:75784981	chr11:74254956	ATTCAATTAAATTTTTTTTCC	ATTCAATTAA-TTTTTTTTCT	NM_032606	91	1	Type-IIa	0	0	0
Non-CpG	CpG	chr16:67204745	chr20:51446859	CGGGGAAGAA-CCCTCACCCT	CGGGGAAGAAGCCCTCACCCT	NM_003946	91	1	Type-IV	0	0	0
Non-CpG	CpG	chrX:27826376	chrX:27362798	TCCAGAGCTTATTGTTTGGTGAT	TCCAGGGCTT---GTTTGGTGAT	NM_182506	91	3	Type-IV	0	0	0
Non-CpG	CpG	chrX:27826376	chrX:27362798	TCCGAGAGGA---CTTAATATTA	TCCGAGAGGAGGACTTAATATTA	NM_182506	91	3	Type-IIb	0	0	0
Non-CpG	CpG	chrX:27826376	chrX:27362798	GCCACAGAAA-GTCACGCCAC	GCCACAGAAAAGTCACGCCAC	NM_182506	91	1	Type-IIa	0	0	0
Non-CpG	CpG	chrX:27826376	chrX:27362798	CATTATTCTT-CTGGAGAGTC	CATTATTTTTTCTGGAGAGTC	NM_182506	91	1	Type-IIa	0	0	0
Non-CpG	CpG	chr2:39005637	chr13:39254985	AAGAAAAAAA---GATTATTTGA	AAGAAAAAAAAGAGATTATTTGA	NM_024775	91	3	Type-I	0	0	0
Non-CpG	CpG	chr2:39005637	chr13:39254985	TTTGTCAGGG--TTAAACGTTA	TTTGTCAGGTACTTAAACGTTA	NM_024775	91	2	Type-IIa	0	0	0
Non-CpG	CpG	chr2:39005637	chr13:39254985	ATTTTAAGGGGCAGTTGTCAT	ATTTTAAGGA-CAGTTGTCAT	NM_024775	91	1	Type-IIa	0	0	0
Non-CpG	CpG	chr2:209120139	chr12:90024930	AGAAGCGGAGGCACTGGGAGTCCGGTTTGG	AGAAGCCGAG----------TCCGGTTTGG	NM_005896	91	10	Type-IV	0	0	0
Non-CpG	CpG	chr1:28655835	chr1:27246053	GGCTCTCTCTCTCTTTTTTTTTTTTTTTTTTTTTTTTGA	GGCTCTCTCT------------------TTTTTTTTGA	NM_017638	91	18	Type-I	0	0	0
Non-CpG	CpG	chr1:67218440	chr1:66525663	GTCTGCAGTG------CCGGAGGTCT	GTCTGCAGTGAACGAGCCGGAGGTCT	NM_152665	91	6	Type-IV	0	1	-1
Non-CpG	CpG	chr1:67218440	chr1:66525663	GTAGGGTCGCCTCTCTCTGCA	GTAGGGTCGC-TCTCTCTGCA	NM_152665	91	1	Type-I	0	0	0
Non-CpG	CpG	chr1:67218440	chr1:66525663	GCAGCGCGTCTGGACCCCAAAGGAAGGATAGGGG	GCAGCGCGTC--------------AGGACAGGGG	NM_152665	91	14	Type-IIb	0	0	0
Non-CpG	CpG	chr10:73611306	chr9:60518569	AAAAGGTATT-TTACATATAT	AAAAGGTATTGTTACATATAT	NM_001042465, NM_001042466, NM_002778	90	1	Type-IIa	0	0	0
Non-CpG	CpG	chr10:73611306	chr9:60518569	CCATTGCCCCCTCCTATTGGCC	CCATTGTCCC--CCAATTGGCC	NM_001042465, NM_001042466, NM_002778	90	2	Type-IV	0	0	0
Non-CpG	CpG	chr10:73611306	chr9:60518569	ATCTGCGGGGGATCAGCTGAC	ATCTGCGGGG-ATCAGCTGAC	NM_001042465, NM_001042466, NM_002778	90	1	Type-IIa	0	0	0
Non-CpG	CpG	chr10:73611306	chr9:60518569	AGGGGGCGCGCGCACTGCGCAG	AGGGGGCGCG--CACTGCGCAG	NM_001042465, NM_001042466, NM_002778	90	2	Type-I	1	0	1
Non-CpG	CpG	chr10:73611306	chr9:60518569	TCGCTGGCTTTCTCTGGCTTTCTCTGGGCG	TCGCCGGCTT----------TCTCTGGGCG	NM_001042465, NM_001042466, NM_002778	90	10	Type-IIc	0	0	0
Non-CpG	CpG	chr11:66278406	chr14:7915181	AGGACTCTTA-AGAGGTCAGA	AGGACTCTTATAGAGGTCAAA	NM_024649	90	1	Type-IIa	0	0	0
Non-CpG	CpG	chr11:74178894	chr14:66552647	GAGGGAAAAG----GGAGCCGAGC	GAGGGAAAAGAAAGGGAGCCGAGC	NM_005472	90	4	Type-IIa	0	0	0
Non-CpG	CpG	chr11:74178894	chr14:66552647	CGGTCAGAGGGCGCCGTGTCCAGCGAGCA	CGGTCAGAGG---------CCAGCGAGCA	NM_005472	90	9	Type-IV	2	0	2
Non-CpG	CpG	chr19:16178648	chr19:15997790	TTTTTTTTTT----CTTCCTTTAA	TTTTTTTTTTTTTTCTTCCTTTAA	NM_003290	90	4	Type-IIa	0	0	0
Non-CpG	CpG	chr16:67185195	chr20:51427239	TTCTTCCCAATCCCCAGAAAG	TTCTTCTCAA-CCCCGGAAAG	NM_033309	89	1	Type-IV	0	0	0
Non-CpG	CpG	chr16:67185195	chr20:51427239	CCCAGGGCCCGACATTGAGGG	CCCAGGGCCC-ACGTTGAGGG	NM_033309	89	1	Type-IV	1	0	1
Non-CpG	CpG	chr16:67185195	chr20:51427239	GATGGCAGGG-TGGAAGGGAT	GATGGCAGGGGTGGAAAGGAT	NM_033309	89	1	Type-IIa	0	0	0
Non-CpG	CpG	chr16:67185195	chr20:51427239	CGAGGCTCCCAGTCTCCCTGC	CGAGACTCCC-GTCTCCCTGC	NM_033309	89	1	Type-IV	0	1	-1
Non-CpG	CpG	chr1:214162237	chr1:152524420	CCAATTATCATATTCATCACC	CCAATTATCA-ATTC-TCACC	NM_002763	89	1	Type-I	0	0	0
Non-CpG	CpG	chr1:214162237	chr1:152524420	TATCATATTCATCACCCGCAA	TATCA-ATTC-TCACCCGCAA	NM_002763	89	1	Type-III	0	0	0
Non-CpG	CpG	chr1:214162237	chr1:152524420	AAAAAAGAGAGAGAGAGAGAT	AAAAGAGAGA-AGAGAGAGAG	NM_002763	89	1	Type-I	0	0	0
Non-CpG	CpG	chr1:214162237	chr1:152524420	AGAGAGAGAGAGGCTCGGTCC	AGAGAGAGAG-GGCTCGGTCC	NM_002763	89	1	Type-I	0	0	0
Non-CpG	CpG	chr1:214162237	chr1:152524420	TCGTGAGTCC-CTCCCCTTTT	TCGTGAGTCCGCTCCGCTTTT	NM_002763	89	1	Type-IIa	0	1	-1
Non-CpG	CpG	chr1:214162237	chr1:152524420	AGCCTCCGTGGAACTCAGCGC	AGCCTCCGTG-AACTCAGCGC	NM_002763	89	1	Type-IIa	0	0	0
Non-CpG	CpG	chr14:20937865	chr7:81686491	GGATCGGAGC--------------------ACACCGGAGC	GGATCGGAGCGGATCGGAGCACACCGGAGCACACCGGAGC	NM_000270	88	20	Type-IV	0	3	-3
Non-CpG	CpG	chr14:20937865	chr7:81686491	GGTGCTGTGA--CCCGGGAACC	GGTGCTGTGAGACCCGGGAACC	NM_000270	88	2	Type-I	0	0	0
Non-CpG	CpG	chr15:32934211	chr7:8486481	TGAGACTGAG----GAGGTGAATG	TGAGATTGAGAAAGGAGGTGAATG	NM_003020	88	4	Type-IIa	0	0	0
Non-CpG	CpG	chr15:32934211	chr7:8486481	TGTGCGGCTCCTGGTGAGAGGC	TGTGCGGCTC--GGTGAGAGGC	NM_003020	88	2	Type-IIa	0	1	-1
Non-CpG	CpG	chr15:32934211	chr7:8486481	TATTGCCAGTTGTGGGAGGGC	TATCGCCAGT-GGGGGAGGGC	NM_003020	88	1	Type-IIa	0	0	0
Non-CpG	CpG	chr15:32934211	chr7:8486481	GATGCGTAGGGGATTAGAATGT	GATGGGTAGG--ATTAGAATGT	NM_003020	88	2	Type-IIa	0	0	0
Non-CpG	CpG	chr19:22035167	chrUn_NW_014922128v1:24711	AGCTCACTTG---ATTAGCTGTA	AGCTCACTTGTTAATTAGGTGTA	NM_001256653	88	3	Type-IIa	0	0	0
Non-CpG	CpG	chr19:22035167	chrUn_NW_014922128v1:24711	TGGGCATGTG-CCCCGCCTAC	TGGGCACGTGACCCCGCCCAC	NM_001256653	88	1	Type-IIa	0	0	0
Non-CpG	CpG	chr19:22035167	chrUn_NW_014922128v1:24711	GGTCCTGGAG--------------------CTTTATCCAA	GGTCCTGGAGGGACCTGGTGGGTCCCGGAGCTTTATCCAA	NM_001256653	88	20	Type-IV	0	1	-1
Non-CpG	CpG	chr19:22035167	chrUn_NW_014922128v1:24711	ACGCGCAGTTGGAGAGGGGGCC	ACGCGCAGTT--AGAGAGGGCC	NM_001256653	88	2	Type-IV	0	0	0
Non-CpG	CpG	chr19:22035167	chrUn_NW_014922128v1:24711	GTCCGCTCCT-GAGTCCTGGT	GTCCGCTCCTTGAGTCCTGGT	NM_001256653	88	1	Type-IIa	0	0	0
Non-CpG	CpG	chr2:21267245	chr13:21267023	CCCCCTCCCC-GAGGCTCTTC	CCCCCTCCCTTGAAGCTCTTC	NM_000384	88	1	Type-IIb	1	0	1
Non-CpG	CpG	chr2:21267245	chr13:21267023	CTGGCGCTGCCTGCGCTGCTGCTGCTGCT	CCGGCGCTGC------------TGCTGCTGCT	NM_000384	88	12	Type-III	1	0	1
Non-CpG	CpG	chr1:72748484	chr1:72169400	GGAGGAGGGG-AGGCAAAAAC	GGAGGAGGGGGAGGCAAAAAC	NM_173808	88	1	Type-IIa	0	0	0
Non-CpG	CpG	chr1:72748484	chr1:72169400	AAAAAAAAAA-TCCTGTGGCG	AAAAAAAAAAATCCTGTGGCG	NM_173808	88	1	Type-IIa	0	0	0
Non-CpG	CpG	chr12:96336686	chr11:95791168	CGCGCGGGTG----------------GCGGGTGCCC	CGCGCGGGTGCCGCCACCCTGAGGTGGCGGGTGCCG	NM_182496	87	16	Type-IV	0	1	-1
Non-CpG	CpG	chr12:96336686	chr11:95791168	ATCCAAACTC------GCTGAGGTAA	ATCCAAACTCAAACTCGTTGAGGTAA	NM_182496	87	6	Type-IIc	1	1	0
Non-CpG	CpG	chr2:60780826	chr13:61337716	TTAAAAAAAA-GCCATGACGG	TTAAAAAAAAAGCCATGACGG	NM_022893, NM_018014, NM_138559, NM_138553	87	1	Type-IIa	0	0	0
Non-CpG	CpG	chr2:60780826	chr13:61337716	CGCCGCCGCC-------CGCCCCGCAG	CGCCGCCGCCGCCCGCCCGCCCCGCAG	NM_022893, NM_018014, NM_138559, NM_138553	87	7	Type-IIb	0	2	-2
Non-CpG	CpG	chr6:57182728	chr4:58300039	AACCGTTAAA-GCTACCGACT	AACCGTTAAAAGCTACCTACT	NM_000947	87	1	Type-IIa	0	0	0
Non-CpG	CpG	chr6:57182728	chr4:58300039	TTGGAAAAAA--AATCCCCAAA	TTGGAAAAAAGAAATCCCCAAA	NM_000947	87	2	Type-IIa	0	0	0
Non-CpG	CpG	chr6:57182728	chr4:58300039	GGCGGGTTGG--------------------TTGTTTGGGG	GGCGGGTTGAGAGTAGTGGGTATTGGTTGATTGGTTGGGG	NM_000947	87	20	Type-IV	0	0	0
Non-CpG	CpG	chr12:6960732	chr11:7160098	GGGCCTATGGGAACTACGTCG	GGGCCTATGG-ATCTACGTCG	NM_031299	86	1	Type-IIa	0	0	0
Non-CpG	CpG	chr16:67217767	chr20:51459981	TGTCCTCTCC-GGGGCTTCTC	TGTCCTCTCCCGGGGCTTCTT	NM_001040715	86	1	Type-IIa	1	1	0
Non-CpG	CpG	chr16:67217767	chr20:51459981	GGGGCTTCTC-TCCGCACAGG	GGGGCTTCTTATCCGCACAGG	NM_001040715	86	1	Type-IIb	0	0	0
Non-CpG	CpG	chr16:67217767	chr20:51459981	AAGAATGGTTAAGAGTTTGCTC	AAGAATGGTT--GAGTTTGCTC	NM_001040715	86	2	Type-IIc	0	0	0
Non-CpG	CpG	chr19:58341979	chr19:51442803	TCTGCTGCACAGAGGCGACTC	TCCGCTGCAC-GAGGCGACTC	NM_032828	86	1	Type-I	0	1	-1
Non-CpG	CpG	chr19:58341979	chr19:51442803	TTGTGGTGCCTTCCATGCCCTCAGGTCAC	TTGTGGCGCC---------CTCAGGTCAT	NM_032828	86	9	Type-IV	0	0	0
Non-CpG	CpG	chr1:235531070	chr1:211651319	CGCGCTTTCCCTGGGCTGCTC	CGCGCGTTCC-TGGGCGGCTC	NM_003193	86	1	Type-IIa	0	0	0
Non-CpG	CpG	chr1:235531070	chr1:211651319	GCCTGCTCCG---TGCGCTGGGA	GCCTGTTCCGAGCGGCTTTGAACCAGCCCAGAAGTGCGGGCCCGAAGGGAATGCGTGT<br>GTGCTGGGA	NM_003193	86	47	Type-IV	0	4	-4
Non-CpG	CpG	chr7:1122144	chr3:34999617	CATGGGCCGT-TGGTCTGAGT	CATGGGCCGTCTGGCCTGAGT	NM_001039966, NM_001505	86	1	Type-IIa	0	0	0
Non-CpG	CpG	chr12:48152921	chr11:45974443	GGGGATACTCTGGGCTCAGGCG	GGGGATGCTC--GGCTCAGGCG	NM_006105	85	2	Type-I	0	1	-1
Non-CpG	CpG	chr12:48152921	chr11:45974443	GTCCACTCTG-GTACCCCCGG	GTCCACTCTACGTATCCCCGG	NM_006105	85	1	Type-IIa	0	1	-1
Non-CpG	CpG	chr14:23504399	chr7:84381960	GCAGGTACGTCCTTTTAGAATTTAAGGTTAG	GCAGGTACGT-----------TTAAGATTAG	NM_002797	85	11	Type-IIc	0	0	0
Non-CpG	CpG	chr19:36524031	chr19:31968229	CTCCTCCCCCGCCGCCGGTAGG	CTCCTCCCCT--CGCCGGTAAG	NM_015526	85	2	Type-IIb	1	0	1
Non-CpG	CpG	chr19:36524031	chr19:31968229	TAAACAAACCCGGGGAGGGGG	TAAACAAACC-GGGGAGGGGG	NM_015526	85	1	Type-IIa	1	1	0
Non-CpG	CpG	chrX:99986818	chrX:94888944	CCGCATCCCC-AGGCTTGGAC	CCGCACCCCCCAGGCTTGGAC	NM_080737	85	1	Type-IIa	0	0	0
Non-CpG	CpG	chrX:99986818	chrX:94888944	ACATCGCGGC------TTCTCTGGCG	ACATCTCGGCGTCGGCTTCTCTGGCG	NM_080737	85	6	Type-I	0	2	-2
Non-CpG	CpG	chrX:99986818	chrX:94888944	TGTGGAGCTGCTTTATATTGC	TGTGGAGCTG-TTTATCTTGC	NM_080737	85	1	Type-III	0	0	0
Non-CpG	CpG	chr1:43833982	chr1:42856738	GGCACGCCCC------TGCCCCCGCC	GGCCCGCCCCCAGCCTCTGCCCCCGCC	NM_022821	85	6	Type-IIb	0	0	0
Non-CpG	CpG	chr1:43833982	chr1:42856738	CCAGCCCGGCCCAGCCCGGCCCAGCCCTGC	CCAGCCCGGC----------CCAGCTCTGC	NM_022821	85	10	Type-IIb	1	0	1
Non-CpG	CpG	chr7:33169467	chr3:74955018	ACGGCGTGACCTGCGCAGTGG	GCGGCGTGAC-TGCGCAGTGG	NM_001033605, NM_198428, NM_001033604, NM_014451	85	1	Type-IIa	0	0	0
Non-CpG	CpG	chr7:33169467	chr3:74955018	TCTCTGGGGG-TGACGGGGGG	TCTCTGGGGGGTGACGGGGGG	NM_001033605, NM_198428, NM_001033604, NM_014451	85	1	Type-IIa	0	0	0
Non-CpG	CpG	chr11:47236845	chr14:18813805	AAAAAAAAAAAAAATCCATAAAGCCG	AAAAATAAAA------CATAAAGCCG	NM_000107	84	6	Type-IIa	0	0	0
Non-CpG	CpG	chr11:47236845	chr14:18813805	GCACCGCCCCTTGGCACCACCCCCTCCCCGCGC	GCACCGCCCC-------------CTCCCCGCGC	NM_000107	84	13	Type-IV	0	0	0
Non-CpG	CpG	chr11:47236845	chr14:18813805	GTTGGCTTAGCTCGGCTACCT	GTTGGCTTAG-TCGGCTACCT	NM_000107	84	1	Type-IV	0	0	0
Non-CpG	CpG	chr1:145438790	chr1:121630908	CGGGAGAAGACGCTCAACAGCC	CGGGTGAAGA--CTCAACAGCC	NM_006472	83	2	Type-IV	1	0	1
Non-CpG	CpG	chr1:145438790	chr1:121630908	CCTCTCCTTCCTCTCCTTCCCCCCTCCCT	CCTCTCCTTC---------CCCCCTCCCT	NM_006472	83	9	Type-IIb	0	0	0
Non-CpG	CpG	chr1:145438790	chr1:121630908	AAAACCTAGTAGTTAATATTCAT	AAAACCTAGT---TAAAATTCAT	NM_006472	83	3	Type-III	0	0	0
Non-CpG	CpG	chr11:89868143	chr14:82569691	TCGTGATTCTCTGCAGAGATAA	TCGTGATTCT--GCAGAGATAA	NM_005467	83	2	Type-I	0	0	0
Non-CpG	CpG	chr12:51718238	chr11:49645179	TGGCTCAGGGCTGGGGCCGGATGCGA	TGGCTCAGGG------CCGGATGCGA	NM_016293	83	6	Type-IIb	0	0	0
Non-CpG	CpG	chr12:51718238	chr11:49645179	GCCCACAGGGAGCCGAGGCCGAGGCCG	GCCCACAGGA-------GCCGAGGCCG	NM_016293	83	7	Type-IIc	1	0	1
Non-CpG	CpG	chr5:137514618	chr6:135890531	CCTAAGAGCC-TAAAGGAAAT	CCTAAGAACCCTAAAGGAAAT	NM_006696, NM_183359, NM_139199	83	1	Type-IIa	0	0	0
Non-CpG	CpG	chr9:95640620	chr15:44786220	GGCAACCTTCAGGCCATGAAG	GGCAACCTTC-GGCCACGAAG	NM_031486, NM_001007101	83	1	Type-IV	0	1	-1
Non-CpG	CpG	chr9:95640620	chr15:44786220	GTAAGAAGTACCTCTAGGCCTGGGAGGCATT	GTAAGAAGTA-----------GGGAGGCATT	NM_031486, NM_001007101	83	11	Type-IV	0	0	0
Non-CpG	CpG	chr2:208030984	chr12:88925058	CGCCCTCCTC-GAGATCCCAC	CGCCCTCCTCCGAGATCCCAC	NM_003709	82	1	Type-IIb	1	1	0
Non-CpG	CpG	chr2:208030984	chr12:88925058	GAAGGGGGGGGAAGAGAAGCGA	GAAGGGGGGG--AGAGAAGCGA	NM_003709	82	2	Type-IIa	0	0	0
Non-CpG	CpG	chr5:78908564	chr6:76324547	CCAAGTACGC---------CCAGCTCGGG	CCGAGTACGCTCAGCCGTCCCAGCTCGGG	NM_173797	82	9	Type-IV	0	1	-1
Non-CpG	CpG	chr8:103666191	chr8:101372503	AGGGCGGCCCCATCCGTCGGT	AGGGCGGCCC-ATCCGTCGGT	NM_005655, NM_001032282	82	1	Type-IIa	0	0	0
Non-CpG	CpG	chr8:103666191	chr8:101372503	GATCTCCCCCGCCGTCCGCCC	GATCTCCCCC-CCGTCCACCC	NM_005655, NM_001032282	82	1	Type-IIa	1	0	1
Non-CpG	CpG	chr8:103666191	chr8:101372503	GCTCGTTTTCTTAGGATTTCAA	GCTCGTTGTC--AGGATTTCAA	NM_005655, NM_001032282	82	2	Type-IIa	0	0	0
Non-CpG	CpG	chr8:103666191	chr8:101372503	GTAAAGAATG-------------------------TTTTTTGGTA	GTAAAGAATGGTTTGTTTGGTTTTTTTTTTTTTTTTTTTTTTGGTA	NM_005655, NM_001032282	82	25	Type-IIa	0	0	0
Non-CpG	CpG	chr8:103666191	chr8:101372503	CTGGACAGTT-------TTCTTTTAAT	CTGAACAGTTGTTTTTTTTTTTTTAAT	NM_005655, NM_001032282	82	7	Type-IIa	0	0	0
Non-CpG	CpG	chr1:203097730	chr1:163510187	AAGTGGGTGGGCGCAGGGCAG	AAGTGGGTGG-CGCAGGGCAG	NM_000674, NM_001048230	81	1	Type-IIa	0	0	0
Non-CpG	CpG	chr19:57631759	chr19:52165880	TTCCAGAGCCATGGGAGCCGG	TTCCAGAGCC-CGGGAGCCGG	NM_020903	81	1	Type-IIa	0	0	0
Non-CpG	CpG	chr19:57631759	chr19:52165880	ACCCTCCCCCCACCCATCTCTA	ACCCTCCCCC--CCCATCTCTA	NM_020903	81	2	Type-IIa	0	0	0
Non-CpG	CpG	chrY:25365980	chrY:10630374	GTGGGGGACGGCCGTCATCCC	GTGTGGGACG-CCGTCGTCCC	NM_020363	81	1	Type-IIa	0	0	0
Non-CpG	CpG	chr6:46620755	chr4:47744343	CGCGGGTCCCTCTGCGGCTCCG	CGCGGGGCCC--TGCGGCTCCG	NM_016593	81	2	Type-I	0	0	0
Non-CpG	CpG	chr6:46620755	chr4:47744343	AAGAAGCAAG-----GGACGGCAGG	AAGAAGCAAGTGCCAGGACGGCAAG	NM_016593	81	5	Type-IIa	0	0	0
Non-CpG	CpG	chr9:88694536	chr15:102346658	AGATGATAAA-ACTAGTATTG	AGATGATAAATACTAGCATTG	NM_016548, NM_177937	81	1	Type-IIa	0	0	0
Non-CpG	CpG	chr9:88694536	chr15:102346658	GTTTTCTTTATTGTTATGTTTAT	GTTTTGTTTA---TTATGTTTAT	NM_016548, NM_177937	81	3	Type-IIb	0	0	0
Non-CpG	CpG	chr9:88694536	chr15:102346658	TTTTGTTATGATGGGGTCCCAGG	TTTTGTTATG---GGGTCCGAGG	NM_016548, NM_177937	81	3	Type-III	0	0	0
Non-CpG	CpG	chr13:99195709	chr17:80245190	GCTTGGAGACCTTTTTTGCTTGGTGCTTCTGGAGGGTC	GCTTGGAGGC------------------CTGGAGGGTC	NM_003576, NM_001032296	80	18	Type-IV	0	0	0
Non-CpG	CpG	chr13:99195709	chr17:80245190	TCTCTTTTTTTTGGGGAAAAAA	TCTCTTTTTT-GGGGAAGAAA	NM_003576, NM_001032296	80	1	Type-IIa	0	0	0
Non-CpG	CpG	chr13:99195709	chr17:80245190	CTCTGTGGGGACACTTGGATA	CTCCGTGGGG-CACTTGGGTA	NM_003576, NM_001032296	80	1	Type-IIa	0	0	0
Non-CpG	CpG	chr13:99195709	chr17:80245190	CAGGTCCACGCTAGCTGGCTGCTG	CCGGTCCACG----CTGGCGGCTG	NM_003576, NM_001032296	80	4	Type-III	0	0	0
Non-CpG	CpG	chr14:76127489	chr7:138062733	TGTTTGCGGCAGCGGCTGCTGCT	TGTTTGCGGC---GGCTGCTGCT	NM_007176	80	3	Type-IV	1	1	0
Non-CpG	CpG	chr14:76127489	chr7:138062733	TGAGAGGATCCGGAGAGTCTG	TGAGAGGATC-GGAGAATCTG	NM_007176	80	1	Type-IIa	1	1	0
Non-CpG	CpG	chr17:4939021	chr16:4992036	GCAGCACGGAGACGCCCCTCC	GCAGAACGGA-ACGCCCCTCC	NM_017986	80	1	Type-I	0	0	0
Non-CpG	CpG	chr17:4939021	chr16:4992036	GCCCCTCCGGGCAGAGCCCGC	GCCCCTCCGG-CAGAGCCCGC	NM_017986	80	1	Type-IIa	0	0	0
Non-CpG	CpG	chr17:4939021	chr16:4992036	GTTTTGTTTTGTTTTGTTTTTGA	GTTTTGTTTT---TTGTTTTTGA	NM_017986	80	3	Type-IIb	0	0	0
Non-CpG	CpG	chr19:6591425	chr19:6795794	ATAGGAAGATTGAATGTCTCCTGCCTGAAGGTCGGGCGGGGA	ATAGGAAGAT----------------------CGGGCGGGGA	NM_001252	80	22	Type-IV	0	0	0
Non-CpG	CpG	chr19:6591425	chr19:6795794	CCACTGCCTG---CATCCTGGCA	CCACTGCCTGGCCCATCC-GGCA	NM_001252	80	3	Type-IIa	0	0	0

Gap type	Sequence example
Type-I	CGCGCGCG
Type-IIa	CCCCCCCC
Type-IIb	CCGGCCGG
Type-IIc	CCGTCCGT
Type-III	CGTCGT
Type-IV	Unclassfifed
